# Identification of protein biomarkers and signaling pathways associated with prostate cancer radioresistance using label-free LC-MS/MS proteomic approach

**DOI:** 10.1038/srep41834

**Published:** 2017-02-22

**Authors:** Lei Chang, Jie Ni, Julia Beretov, Valerie C. Wasinger, Jingli Hao, Joseph Bucci, David Malouf, David Gillatt, Peter H. Graham, Yong Li

**Affiliations:** 1 Cancer Care Centre, St George Hospital, Kogarah, NSW 2217, Australia; 2 St George and Sutherland Clinical School, Faculty of Medicine, UNSW, Kensington, NSW 2052, Australia; 3 Department of Obstetrics and Gynecology, The First Affiliated Hospital of Zhengzhou University, Zhengzhou, Henan 450052, China; 4 SEALS, Anatomical Pathology, St George Hospital, Kogarah, NSW 2217, Australia; 5 Bioanalytical Mass Spectrometry Facility, Mark Wainwright Analytical Centre, UNSW, Kensington, NSW 2052, Australia; 6 School of Medical Science, UNSW, Kensington, NSW 2052, Australia; 7 Department of Urology, St George Hospital, Kogarah, NSW 2217, Australia; 8 Australian School of Advanced Medicine, Macquarie University, NSW 2109, Australia

## Abstract

Identifying biomarkers and signaling pathways are important for the management of prostate cancer (CaP) radioresistance. In this study, we identified differential proteins and signaling pathways from parental CaP cell lines and CaP radioresistant (RR) sublines using a label-free LC-MS/MS proteomics approach. A total of 309 signaling pathway proteins were identified to be significantly altered between CaP and CaP-RR cells (*p* ≤ 0.05, fold differences >1.5, ≥80% power). Among these proteins, nineteen are common among three paired CaP cell lines and associated with metastasis, progression and radioresistance. The PI3K/Akt, VEGF and glucose metabolism pathways were identified as the main pathways associated with CaP radioresistance. In addition, the identified potential protein markers were further validated in CaP-RR cell lines and subcutaneous (s.c) animal xenografts by western blotting and immunohistochemistry, respectively and protein aldolase A (ALDOA) was selected for a radiosensitivity study. We found the depletion of ALDOA combined with radiotherapy effectively reduced colony formation, induced more apoptosis and increased radiosensitivity in CaP-RR cells. Our findings indicate that CaP radioresistance is caused by multifactorial traits and downregulation of ALDOA increases radiosensitivity in CaP-RR cells, suggesting that controlling these identified proteins or signaling pathways in combination with radiotherapy may hold promise to overcome CaP radioresistance.

Prostate cancer (CaP) is a significant medical problem in developed countries and accounts for an estimated 180,890 new cases and 26,120 deaths in the United States in 2015^1^. Because of widespread prostate-specific antigen (PSA) screening, about 90 percent of patients are diagnosed with localized CaP^2^. Radiotherapy (RT) is a standard treatment option for both organ-confined and regionally advanced CaP. Despite more and more effective advances in radiation delivery procedures, depending on the stage of disease, about 50% CaP patients undergoing RT suffer from relapse (recurrence) within 5 years of treatment^3^. One reason for these failures following RT is due to the radioresistance of a subpopulation of CaP clones within the tumor. Radioresistance is a major challenge for the current CaP radiotherapy. While a number of markers have been identified as prognostic or predictors of recurrence following RT in CaP^4^,^5^, the main limitation for current biomarkers is that they failed to reach clinical application. Therefore, it is critical to investigate CaP-radioresistant (RR) biomarkers and signaling pathways to identify therapeutic targets and develop novel adjuvant treatments to overcome radioresistance.

The development of proteomic techniques has sparked new searches for novel protein markers for many diseases including CaP. Proteomic technology offers a platform for the quantification and identification of novel RR proteins for developing new therapeutic targets to overcome radioresistance. Advances in proteomics, especially in mass spectrometry (MS) have rapidly changed our knowledge of biomarker proteins which have simultaneously led to the identification and quantification of thousands of unique proteins and peptides in a complex biological fluid or cell lysate^6^.

Label-free liquid chromatography/tandem mass spectrometry (LC-MS/MS) is a proteomic technology that combines chromatographic techniques with MS to enhance separation of the very complex biological samples by fractionating peptides into discretely eluting ions that the scan rate of mass spectrometers can manage. This allows reproducible, in-depth coverage of 1000’s of peptides within very complex samples. It is multidimensional and highly sensitive^7^. LC-MS/MS has been used to identify biomarkers from CaP cell lines^8^ as well as serum^9^ and tissue samples^10^ from CaP patients for diagnosis and monitoring progression. This technology has also been applied to lung^11^ and brain^12^ cancer cell lines for RR markers.

Using a low dose fractionated radiation treatment, we recently developed three CaP-RR cell lines with increased colony formation, invasion ability, sphere formation and enhanced epithelial-mesenchymal transition (EMT) and cancer stem cell (CSC) phenotypes and the activation of the PI3K/Akt/mTOR signaling pathway^13^. These CaP-RR cells, representative of the source of CaP metastasis and recurrence after RT, may provide a very good model to mimic a clinical RR condition and to investigate biomarkers and signaling pathway nets for developing novel approaches for CaP treatment.

In this study, we employed an LC-MS/MS technique to identify the protein difference between CaP and CaP-RR cells and the main signaling pathways involved in CaP radioresistance. In addition, we validated the potential signaling pathway proteins identified in CaP-RR cell lines and CaP-RR subcutaneous (s.c) xenograft tumors. Furthermore, we used small interfering RNA (siRNA) silencing to explore functions of one of identified proteins, Fructose-bisphosphate aldolase A (ALDOA), for radiosensitivity using CaP-RR cells. Our results indicate a panel of proteins and signaling pathways are involved in CaP radioresistance and targeting these proteins or pathways holds promise to improve CaP radiosensitivity.

## Results

### The difference of proliferation between CaP and CaP-RR Cells

**Figure 1:**
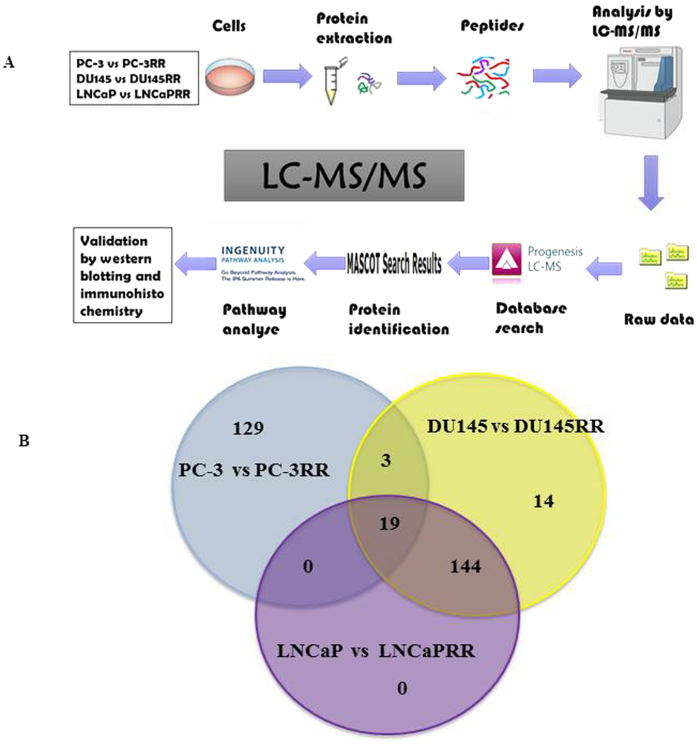
A flow chart of LC-MS/MS analysis and identified potential proteins from CaP and CaP-RR cells. (**A**) A schematic diagram is shown for the brief procedure of LC-MS/MS for protein preparation, data analysis and validation of identified proteins. (**B**) A venn diagram shows the proteins identified from CaP (PC-3, DU145 and LNCaP) and CaP-RR (PC-3RR, DU145RR and LNCaPRR) cells by LC-MS/MS, and the overlapped proteins among three pairs of cells (PC-3 and PC-3RR, DU145 and DU145RR, LNCaP and LNCaPRR).

To evaluate the difference between CaP-RR and CaP-control cells in proliferation, we examined their proliferation rate. Our results showed that the growth of CaP-RR cells was significantly reduced than that in CaP-control cells (*P* < 0.05) (Fig. 1S).

### Altered proteomic content between CaP and CaP-RR Cells

**Figure 2:**
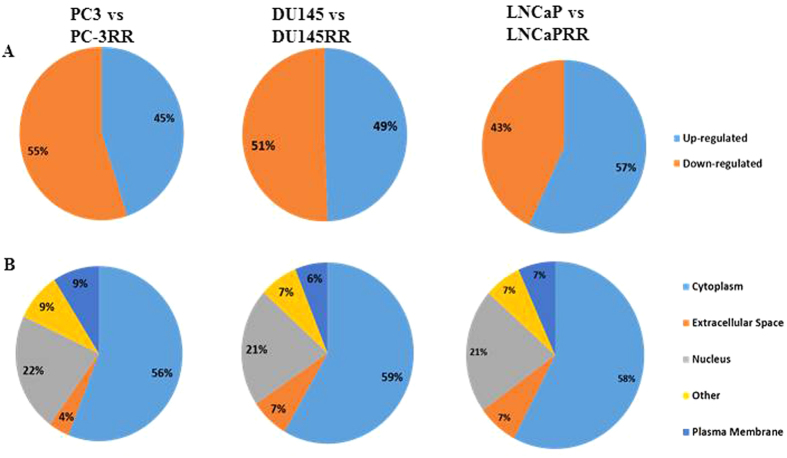
IPA classification of the identified proteins for expression and subcellular distribution between CaP and CaP-RR cell lines. (**A**) Expression profiles of the identified proteins between CaP (PC-3, DU145 and LNCaP) and CaP-RR (PC-3RR, DU145RR and LNCaPRR) cell lines. (**B**) Subcellular distribution of the identified proteins between CaP (PC-3, DU145 and LNCaP) and CaP-RR (PC-3RR, DU145RR and LNCaPRR) cell lines.

The differentially expressed proteins between CaP and CaP-RR cell lines were identified and quantitated by Progenesis QI software. The entire process of LC-MS/MS is briefly summarized and shown in Fig. 1A. Principal component analysis (PCA) analysis revealed that 90–98% differences could be identified between CaP and CaP-RR cell lines (Fig. 2S). For proteomic protein profile analysis, a total of 299 significant proteins abundance changes (*p* < *0.05*) were identified between PC-3 and PC-3RR cells (Supplemental Table S1), 391 proteins between DU145 and DU145RR cells (Supplemental Table S2) and 360 proteins between LNCaP and LNCaPRR cells (Supplemental Table S3). This represents ~6% protein alteration from the total proteome identified. Across all cell types, a total of 309 signaling pathway proteins identified were mapped to be statistically significant between CaP (PC-3, DU145 and LNCaP) and CaP-RR (PC-3RR, DU145RR and LNCaPRR) cells (*p* ≤ 0.05, fold differences >1.5, ≥80% power). Nineteen proteins were found to be overlapped among three paired cell lines such as ALDOA, Alpha-2-HS-glycoprotein precursor (AHSG), Vimentin, Tyrosine 3-monooxygenase/tryptophan 5-monooxygenase activation protein, epsilon (YWHAE), and Peroxiredoxin 6 (PRDX6) (Fig. 1B and Table 1). Nine biological replicates and three technical replicates were acquired.

### Important signaling pathways associated with CaP radioresistance

**Figure 3:**
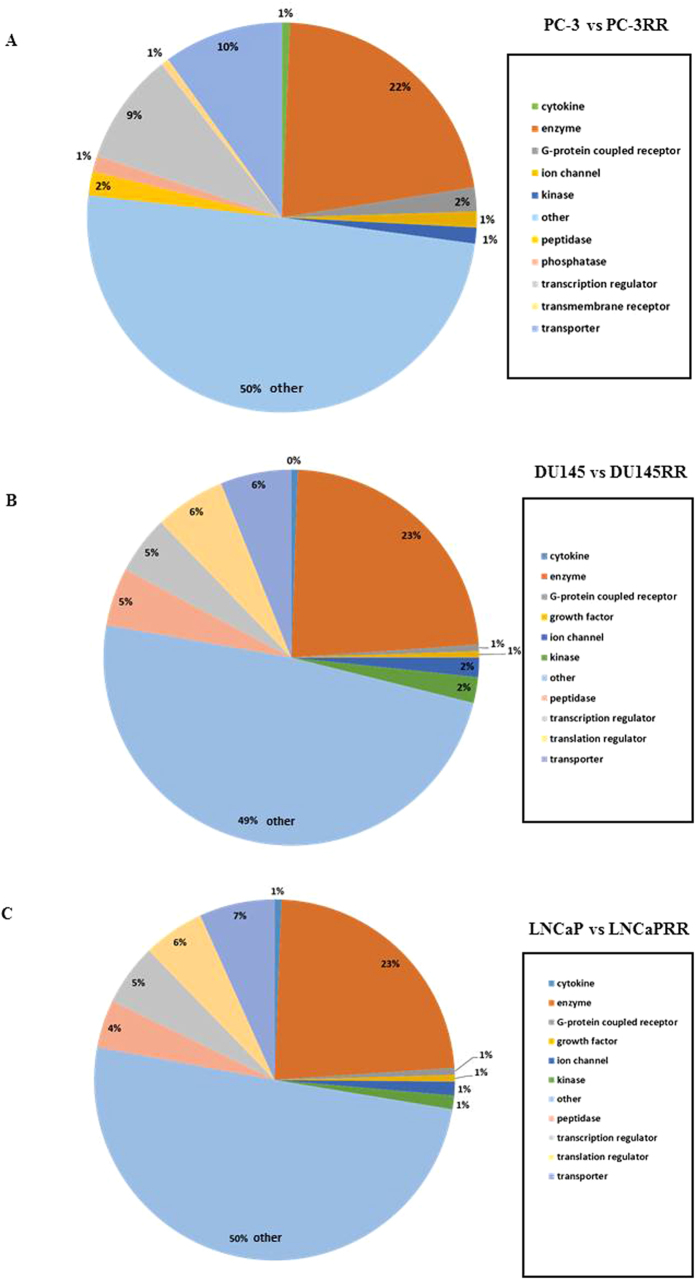
IPA classification of the identified proteins for functional categories between CaP and CaP-RR cell lines. (**A**) Functional categories of the identified proteins between PC-3 and PC-3RR. (**B**) Functional categories of the identified proteins between DU145 and DU145RR. (**C**) Functional categories of the identified proteins between LNCaP and LNCaPRR.

Pathways altered in CaP-RR cells in different paired cell lines were assessed using Ingenuity software (IPA). Of 299 profile proteins identified from PC-3 and PC-3RR paired cell lines, 151 proteins were mapped to over-represented pathways, which included 68 up-regulated proteins (45%) and 83 down-regulated proteins (55%) (Fig. 2A). The main protein locations of these 151 proteins identified included 85 cytoplasm (56%) and 33 nucleus (22%), 14 plasma membrane (9%) and 6 extracellular space (4%) (Fig. 2B). The cellular molecular function analysis revealed that there were 33 enzymes (22%), 15 transporters (10%), 14 transcription regulator (9%) and 7 kinase/peptidase/phosphatases (4%) (Fig. 3A). Similarly, there were 180 mapped proteins out of 391 proteins in DU145 and DU145RR paired cell lines including 89 up-regulated proteins (49%) and 91 down-regulated proteins (51%) (Fig. 2A). The main protein locations of those 180 mapped proteins were 105 cytoplasm (59%), 33 nucleus (21%), 13 extracellular space (7%) and 11 plasma membrane (6%) (Fig. 2B), and the cellular molecular functions were comprised of 42 regular enzymes (23%), 13 kinase/peptidase (7%), 11 transporters (6%) and 9 transcription regulators (5%) (Fig. 3B). Meanwhile, of 360 profile proteins identified from LNCaP and LNCaPRR paired cell lines, 163 proteins were mapped and contained 93 up-regulated proteins (57%) and 70 down-regulated proteins (43%) (Fig. 2A). The main protein locations of 163 mapped proteins were 94 cytoplasm (58%), 35 nucleus (21%), 11 plasma membrane (7%) and 12 extracellular space (7%) (Fig. 2B), and the cellular molecular functions comprised 42 regular enzymes (23%), 11 transporters (7%), 8 kinase/peptidase (5%), and 8 transcription regulators (5%) (Fig. 3C).

**Figure 4:**
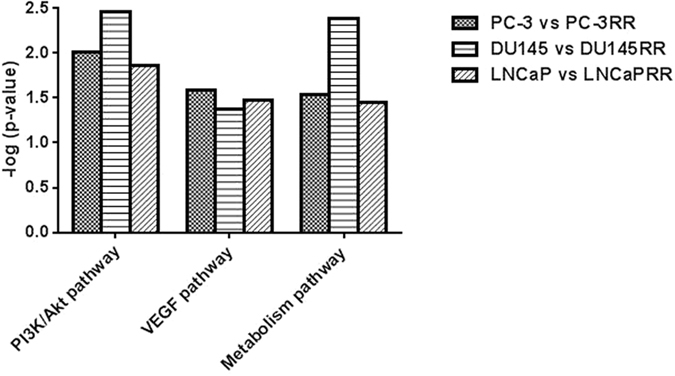
Identifying the top three common potential pathways associated with CaP radioresistance. The *p* value [presented in −log (p-value)] and ratio of the identified top three common potential pathways are displayed by IPA core analysis between CaP (PC-3, DU145 and LNCaP) cells and CaP-RR (PC-3RR, DU145RR and LNCaPRR) cells. The identified top three signaling pathways are PI3K/Akt, VEGF and glucose metabolism pathways.

**Figure 5:**
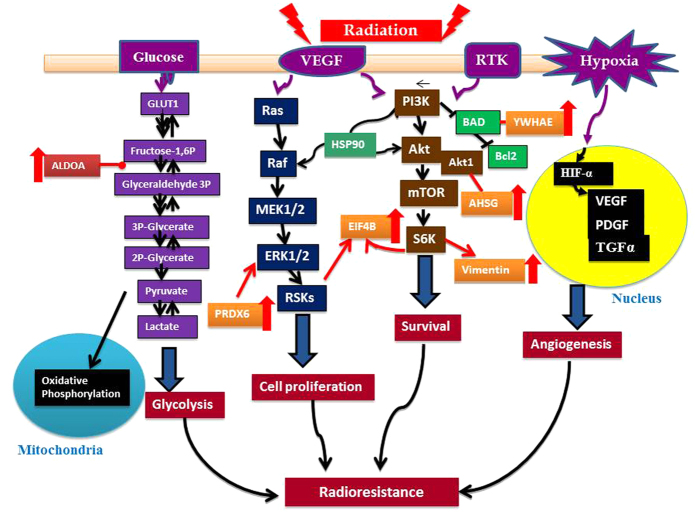
A schematic diagram showing the important signaling pathways and proteins involved CaP radioresistance. After radiotherapy, several signaling pathways associated with CaP radioresistace are activated. The PI3K/Akt/mTOR, VEGF and glucose metabolism pathways are the main pathways involved in CaP radioresistance. ALDOA, PRDX6, EIF4B, YWHAE, AHSG and Vimentin are the common proteins from three paired CaP cell lines (PC-3, DU145 and LNCaP vs PC-3RR, DU145RR and LNCaPRR) identified by LC-MS/MS.

**Figure 6:**
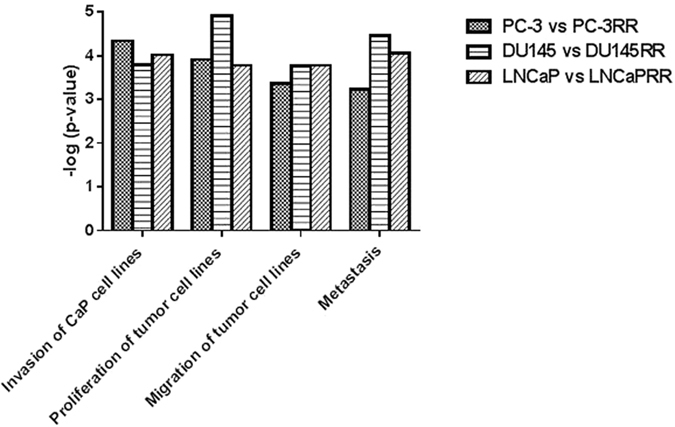
Disease and function analysis of the common important differentially expressed proteins associated with CaP radioresistance in paired CaP cell lines. The *p*-values [presented in −log (p-value)] of differentially expressed proteins in PC-3 vs PC-3RR, DU145 vs DU145RR and LNCaP vs LNCaPRR cell lines are displayed in the graph, respectively. Four selected common diseases or functions of interest are invasion of CaP cell lines, proliferation of tumor cell lines, migration of tumor cell lines and metastasis.

Ingenuity canonical pathway analysis revealed that many important cancer-related signaling pathways were involved in each paired cell lines (Supplemental Tables S4–6). We displayed the *p*-value and ratio of our interested top three common pathways associated with CaP radioresistance including PI3K/Akt/mTOR, vascular endothelial growth factor (VEGF) and glucose metabolism (Fig. 4). The diagram demonstrating the association of these three pathways with radioresistance is shown in Fig. 5. The identified common proteins from three paired CaP cell lines are also involved in the regulation of CaP radioresistance (Fig. 5). The alterations of “disease and function” pathways in CaP-RR cells are summarized in Supplemental Tables S7–9. Through disease and function analysis, we demonstrated the common important differentially-expressed proteins associated with CaP radioresistance in three paired CaP cell lines including invasion of CaP cell lines, proliferation of tumor cell lines, migration of tumor cell lines and metastasis (Fig. 6). The details of these selected common alterations of “disease and function” in three paired CaP cell lines are summarized in Table 2. The findings suggest the common features associated with CaP radioresistance include cancer proliferation, invasion, migration and metastasis.

### Validation of key signaling pathway proteins in CaP cell lines and animal xenografts

**Figure 7:**
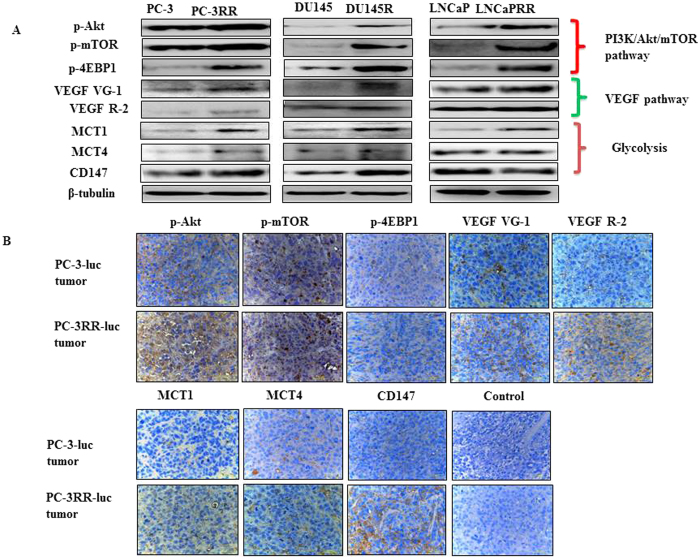
Validation of key pathway proteins from top three pathways identified in CaP-RR cell lines and PC-3RR-luc s.c xenograft tumors. (**A**) The expression of p-Akt, p-mTOR, p-4EBP1, VEGF VG-1, VEGF R-2, MCT1, MCT4 and CD147 pathway associated proteins) was increased in CaP-RR (PC-3RR, DU145RR and LNCaPRR) cells compared with CaP (PC-3, DU145 and LNCaP) cells. β-tubulin was used as a loading control. (**B**) Representative images showing increased expression of p-Akt, p-mTOR, p-4EBP1, VEGF VG-1, VEGF R-2, MCT1, MCT4 and CD147 pathway associated proteins) in PC-3-luc and PC-3RR-luc s.c. xenografts using immunohistochemistry. Brown indicates positive staining while blue indicates nuclear staining. Magnification x 40 in all images. All data were from three independent experiments (n = 3).

To confirm the link between the main proteins from PI3K/Akt/mTOR, VEGF and metabolism (glycolysis) signaling pathways identified and radioresistance, the expressions of representative proteins from each pathway were examined in CaP and CaP-RR cell lines by western blotting, and in PC-3-luc and PC-3RR-luc s.c animal xenografts using immunohistochemistry (IHC), respectively. The expression of p-Akt, p-mTOR, p-4EBP1, VEGFVG-1, VEGFR-2, MCT1, MCT4 and CD147 was found to be significantly increased in CaP-RR cells compared to CaP (control) cells (Fig. 7A). In addition, the increased expression of p-Akt, p-mTOR, p-4EBP1, VEGFVG-1, VEGFR-2, MCT1, MCT4 and CD147 was also found in PC-3-RR-luc s.c animal xenografts compared to PC-3-luc control s.c animal xenografts using IHC (Fig. 7B). The IHC staining results are summarized in Supplemental Table S10. These findings indicate the identified signaling pathways play important roles in CaP radioresistance.

### Verification of potential marker ALDOA in CaP cell lines and animal xenografts

**Figure 8:**
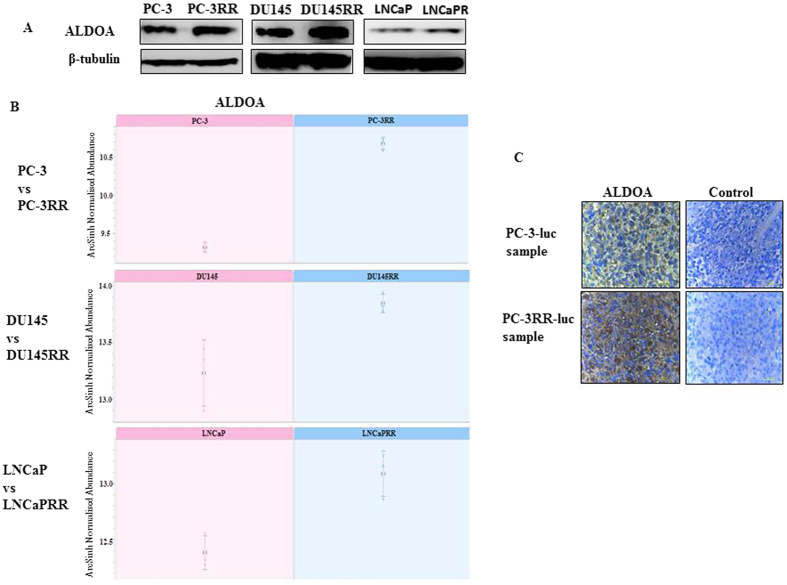
Validation of ALDOA in CaP-RR cell lines and PC-3RR-luc s.c xenograft tumors. (**A**) The expression of ALDOA (potential marker) was increased in CaP-RR (PC-3RR, DU145RR and LNCaPRR) cells compared with CaP (PC-3, DU145 and LNCaP) cells. β-tubulin was used as a loading control. (**B**) The comparison of ALDOA protein in CaP (PC-3, DU145 and LNCaP) and CaP-RR (PC-3RR, DU145RR and LNCaPRR) cells using LC-MS/MS. (**C**) The expression of ALDOA in PC-3-luc and PC-3RR-luc s.c xenografts using immunohistochemistry. Brown indicates positive staining while blue indicates nuclear staining. Magnification x 40 in all images. Representative results are shown. All data were from three independent experiments (n = 3).

ALDOA protein was identified to be associated with radioresistance in three paired CaP cell lines (Table 1) (*p* < 0.05) as well as in the glycolysis signaling pathway (Fig. 5 and Supplemental Tables S4–6). Therefore, we chose ALDOA for further validation in CaP-RR cells as well as in PC-3RR-luc animal xenografts to find whether it is suitable as a therapeutic target to improve CaP radiosensitivity. As shown in Fig. 8A, the increased expression of ALDOA was found in CaP-RR cells compared to CaP cells, which is consistent with our proteomic analysis results (Fig. 8B). To examine the relevance of this finding in the animal study, we detected the expression of ALDOA in PC-3-luc and PC-3-RR-luc s.c tumor xenografts and found the expression of ALDOA was increased in PC-3RR-luc s.c tumor xenografts compared with that in PC-3-luc xenografts (Fig. 8C). The immunostaining results on two s.c animal xenograft tumors are summarized in Supplemental Table S11. All data from proteomics analysis, western blotting and IHC support that ALDOA is associated with CaP radioresistance.

### Combination therapy with suppression of ALDOA and RT increases radiosensitivity

**Figure 9:**
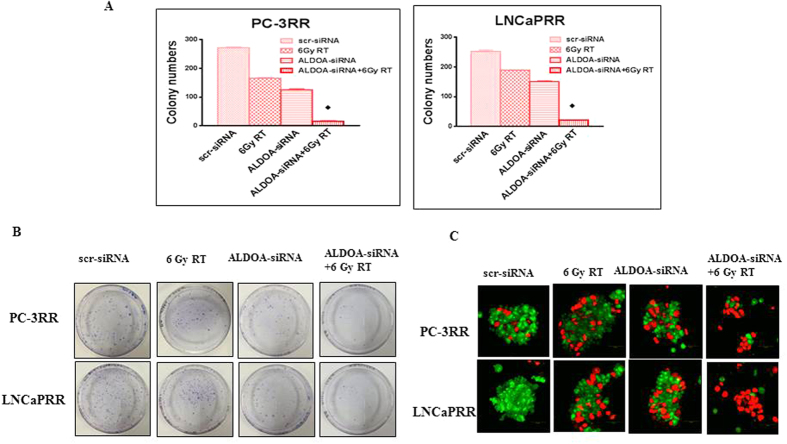
The effect of ALDOA suppression on radiosensitivity of CaP-RR cells. (**A**) Colony formation was significantly reduced in combination treatment with ALDOA-siRNA and RT (6 Gy) compared with 6 Gy RT, ALDOA-siRNA, or scr-siRNA control in CaP-RR (PC-3RR and LNCaPRR) cells (*p < 0.05). (**B**) Typical images of colony growth for the different treatments are shown. (**C**) Combination of ALDOA-siRNA with RT (6 Gy) induced more apoptotic cells in CaP-RR cells compared with 6 Gy RT, ALDOA-siRNA or scr-siRNA control. Red indicates apoptotic cells while green indicates non-apoptotic cells. Magnification x 40 in all images. Representative results are shown. All data were from three independent experiments (Mean ± SD, n = 3).

To investigate whether knock-down (KD) of ALDOA sensitized CaP-RR cells, colony assay was performed in PC-3RR and LNCaPRR cells with the treatment of either scramble (scr)-siRNA, 6 Gy RT, ALDOA-siRNA or combination with ALDOA-siRNA and 6 Gy RT. The number of colonies with a combination treatment of ALDOA-siRNA and 6 Gy RT consistently showed a significant reduction in two CaP-RR cell lines compared to the treatment with ALDOA-siRNA or 6 Gy RT alone as well as scr-siRNA (*p* < *0.05*) (Fig. 9A). Although ALDOA-siRNA or 6 Gy RT alone treatment caused the reduction of colony assay in PC-3RR and LNCaPRR cells compared with the scr-siRNA control cells, no significant difference was found between these two single treatments (*p* > *0.05*). The typical images for colony formation from different treatments are shown in Fig. 9B. In addition, combination of ALDOA-siRNA and 6 Gy RT induced more apoptotic cells, when compared to ALDOA-siRNA alone or 6 Gy RT alone as well as scr-siRNA control (Fig. 9C). The immunofluorescence staining results for AO/EB in PC-3RR and LNCaPRR cells are summarized in Supplemental Table S12. These findings indicate that suppression of ALDOA could increase radiosensitivity in PC-3RR and LNCaPRR cells.

## Discussion

Radioresistance is a main challenge for the current CaP radiotherapy. Identifying protein changes and signaling pathways related to CaP radioresistance is critically important to find therapeutic targets, predict radiation response, develop novel treatment approaches and overcome recurrence after RT. The establishment of RR cancer cell lines is an important step in providing an *in vitro* model for understanding the mechanism of radioresistance and identifying new therapeutic targets.

In the current study, comparison of three established CaP-RR cell lines to CaP parental cell lines identified 19 common protein differences related to 3 significant signaling pathways involved in CaP radioresistance using a label-free LC-MS/MS proteomic technique. In addition, the identified main pathway proteins were further validated in CaP-RR cell lines and PC-3RR-luc tumor xenografts by western blot and IHC, respectively. Furthermore, one selected potential glycolysis marker, ALDOA, was functionally verified in CaP-RR cells for increasing radiosensitivity.

In this study, we established three novel CaP-RR (PC-3RR, DU145RR and LNCaPRR) cell lines derived from clones that had survived after irradiation which represent androgen-responsive (LNCaP) and androgen-nonresponsive (PC-3 and DU145) stages during CaP progression and examined the newly established cell lines with respect to proliferation, invasion and migration, and colony formation after a range of ionizing radiation exposure. We demonstrated that reduced cell proliferation (Fig. 1S), increased invasion and migration and increased colony formation ability in three CaP-RR cell lines compared to untreated CaP-control cell lines^13^, indicating the reduced cell growth and increased progression and radiation resistance in the newly established sublines. In addition, the two cell populations (CaP-RR vs CaP-control cells) were significantly separated by PCA (Fig. 2S). These data confirmed that the CaP-RR cells are radioresistant and obviously different from CaP-control cells, which is suitable for proteomics analysis.

After comparing three paired CaP and CaP-RR cell lines, we identified protein difference varying from 299 to 391. To investigate the association of identified protein profiles with signaling pathways, we found 151/299, 180/391, 163/360 proteins were mapped with pathway proteins in paired PC3/PC-3RR, DU145/DU145RR and LNCaP/LNCaPRR cell lines, respectively, indicating the link of the identified proteins with signaling pathways in CaP radioresistance. These mapped proteins were found to be up-regulated or down-regulated, with different locations in CaP cells including cytoplasm, nucleus, plasma membrane, extracellular space. Our results indicate that the proteins differentially expressed in CaP and CaP-RR cells are associated with signaling pathways which demonstrate multiple functions in CaP radioresistance, suggesting that it is important to investigate these functions in the future studies.

In this study, 19 proteins overlapped among three paired CaP cell lines, which were involved in different functions including glycolysis, EMT, signal transduction and redox. ALDOA was reported to affect the glycolysis pathway in PC-3 cells^14^ and functions as an oncogene in the highly metastatic pancreatic cancer^15^. AHSG is a tumor antigen found in glioblastoma, breast cancer and pancreatic cancer^16^. As glycolytic proteins, ALDOA and AHSG were both up-regulated in CaP-RR cell lines analyzed by LC-MS/MS, indicating glycolysis is involved in CaP radioresistance. Recent studies demonstrated that EMT affects therapeutic resistance^17^. Vimentin is a symbol of the acquisition of mesenchymal characteristics. In this study, 2-, 6- and 7-fold changes of Vimentin were found to be increased in CaP-RR (PC-3RR, DU145RR, LNCaPRR) cells compared with CaP (PC-3, DU145 and LNCaP) cells, respectively, indicating that EMT is correlated with CaP radioresistance. This result is also in line with our previous report^13^. YWHAE gene belongs to the 14-3-3 family that is involved in metabolism, protein trafficking, signal transduction, evasion of apoptosis, cell cycle regulation, cell death and mitogenesis^18^,^19^. PRDX6 is located in the cytosol and functions as antioxidant and regulator of hydrogen peroxide-mediated signaling^20^. Li *et al*. demonstrated that both YWHAE and PRDX6 were found to be up-regulated in highly metastatic breast cancer cells by western blotting and immunofluorescence^17^,^21^. In addition, overexpression of PRDX6 was reported to be significantly correlated with the presence of lymph node metastasis in breast cancer^22^. In this study, high levels of YWHAE and PRDX6 expression were identified in CaP-RR cell lines compared with CaP cells, indicating both markers are associated with CaP radioresistance. All in all, our findings suggest that the identified common proteins from three paired cell lines are associated with CaP metastasis, progression, signaling pathways and radioresistance, and that combination of three paired CaP cell lines may be more likely to reflect the different *in vivo* scenarios.

Accumulating evidence from human CaP tissues and preclinical studies demonstrates that the important signaling pathways play a critical role in CaP progression, metastasis and chemo-/radio-resistance via the activation of the pathway proteins or mutation, deletion, epigenetically silence of some pathway genes^13^,^23^. The PI3K/Akt/mTOR pathway regulates cell growth and proliferation and is often dysregulated in cancer due to mutation, amplification, deletion, methylation and post-translational modifications. This pathway is an intracellular signaling pathway important for apoptosis, malignant transformation, tumor progression, metastasis and radioresistance^13^,^24^. Using two-dimensional difference in gel electrophoresis (2DDIGE)-MS proteomics study, Skvortsova *et al*. found the PI3K/Akt/mTOR signaling pathway was an important intracellular signaling pathway regulating CaP radioresistance using three CaP-RR cell lines, LNCaP-IRR, PC3-IRR, and DU145-IRR derived from the parental LNCaP, PC-3, and DU145 CaP cells by repetitive exposure to ionizing radiation^25^, demonstrating the importance of this signaling pathweay in CaP radioresistance. In the current study, we also found the PI3K/Akt/mTOR signaling pathway is one of main pathways associated with CaP radioresistance. This finding is consistent with our previous results in CaP-RR cell lines with western blotting analysis^13^. In addition, we also demonstrated that combination of dual PI3K/mTOR inhibitors with RT could overcome CaP radioresistance *in vitro*^26^. Our results here further confirme that this pathway could be used as therapeutic targets for CaP radiotherapy.

VEGF signaling pathway which is stimulated by upstream activators including environmental cues, growth factors, oncogenes, cytokines and hormones is a critically important growth factor pathway stimulating vasculogenesis and angiogenesis. Overexpression of VEGF contributes to the growth and metastasis of solid tumors and inhibition of VEGF pathway offers potential clinical treatment for patients^27^. It was reported that radioresistance is linked with VEGF-VEGFR-2 (KDR) interplay in glioblastoma cells^28^. Miyasaka *et al*. demonstrated that the PI3K/mTOR pathway inhibition could overcome radioresistance via suppression of the HIF1-α/VEGF pathway in endometrial cancer and targeting the PI3K/mTOR or HIF-1α pathways could improve radiosensitivity^29^. The radiation-enhanced VEGF secretion with an increased angiogenic potential of the tumor may be a factor in radioresistance^30^. Blocking radiation-induced VEGF pathway in CaP could interfere with tumor growth^23^. Similar evidence was also shown in head and neck squamous cell carcinoma (HNSCC)^31^,^32^. Our current study firstly demonstrated that VEGF pathway is involved in CaP radioresistance, suggesting that targeting this pathway is promising for CaP radiotherapy.

Glycolysis pathway, located in the cytoplasm of eukaryotic cells, is the metabolic pathway which is responsible for the production of adenosine triphosphate (ATP) through the degradation of glucose. Tumor process can be caused by energy metabolism resulted from the increased glycolytic pathway^33^. Meng *et al*. recently reported that targeting pyruvate kinase M2 (a key regulator of glycolysis) contributes to radiosensitivity of non-small cell lung cancer cells *in vitro* and *in vivo*^34^. Shimura *et al*. found that AKT-mediated enhanced aerobic glycolysis causes acquired radioresistance by human tumor cells^35^. Lactate dehydrogenase 5 isoenzyme, a marker of tumor anaerobic metabolism, is significantly linked to highly proliferating CaP and with biochemical failure and local relapse following RT^36^. Meijer *et al*. demonstrated that targeting HIF-1 and tumor glucose metabolism at several levels reduced the antioxidant capacity of tumors, affected the tumor microenvironment, and sensitized various solid tumors to irradiation^37^. Bing *et al*. also found that glycolysis might impede radiation treatment in RR cancer cell lines^38^. In our study, glycolysis pathway was found to be activated in CaP-RR cells and further confirmed in CaP-RR xenograft tumor tissues (see below discussion). These results indicate that glycolysis pathway is involved in radioresistance in CaP and targeting this pathway is likely to have broad therapeutic applications for cancer radioresistance.

To verify the potential biomarkers identified from CaP-RR cell lines, we further validate the selected pathway proteins from the top three signaling pathways associated with CaP radioresistance in three CaP-RR cell lines (PC-3RR, DU145RR and LNCaPRR) and PC-3RR-luc s.c xenograft tumors. The reasons for choosing the CaP-RR s.c animal model are that mouse model can be conducted under stringent genetic and environmental control, which has proven beneficial when investigating the fundamentals of cancer biology and biomarker verification^39^,^40^ and that mouse model can mimic human cancers to an ever greater extent. In this study, we found significant increase of key pathway proteins in CaP-RR cells and PC-3RR-luc xenograft tumors, further confirming that these three signaling pathways play important roles in CaP radioresistance. These findings indicate targeting these signaling pathways combined with RT could improve CaP radiotherapy.

Tumor cell metabolic pathway is an attractive target to eliminate RR cells and improve RT efficacy^35^. Glycolysis is a main catabolic pathway of glucose metabolism, accompanied by ATP synthesis and most cancer cells exhibit increased glycolysis process. Although more than 30 enzymes are involved in glycolysis, the metabolic alterations in CaP radioresistance remain unknown. ALDOA is a glycolytic enzyme and catalyzes the reversible reaction of fructose-1, 6-bisphosphate to glyceraldehydes-3-phosphate and dihydroxyacetone phosphate which is an important part of glycolysis process^41^. High level expression of ALDOA was found in metastatic lung squamous cell carcinoma (LSCC) and the depletion of its expression could reduce the capabilities of cell motility and tumorigenesis^41^. Up-regulated ALDOA expression was also observed in oral squamous cell carcinomas by quantitative real-time polymerase chain reaction (qRT-PCR)^42^. Chen *et al*. showed that the expression of ALDOA was significantly higher in patients with worse survival time than those with better survival time in human osteosarcoma^43^. A five-year survival analysis also showed there is a statistically significant difference between two patient populations of better and worse prognosis^44^. In addition, over-expression of ALDOA was found in PC-3 docetaxel resistant cell line using the label-free LC-MS method^45^. In the current study, we firstly found overexpression of ALDOA in three CaP-RR cell lines compared with CaP cell lines by LC-MS/MS analysis (see Fig. 8B) and confirmed the high level expression of ALDOA in CaP-RR cell lines and PC-3RR-luc xenograft tumors, respectively. To further investigate the value of the ALDOA for future clinical trials, ALDOA was selected for functional studies as a proof of concept. We found that KD of ALDOA combined with 6 Gy RT resulted in significant reduction in colony formation capability and inducing more apoptosis, suggesting that ALDOA is a potential therapeutic target for clinical treatment of CaP radioresisitance. More work investigating the role of ALDOA in CaP radioresistance using a gene transfection or knockout model will be considered in our future study. As many interesting potential proteins were identified in the current study, we will expand our proof of concept experiments to multiple RR proteins in our following studies. In addition, the mechanisms of these RR proteins in CaP metastasis and recurrence after RT will be deeply investigated. Furthermore, these RR proteins will be validated in human CaP-RR tissue samples to further confirm their clinical significance.

In conclusion, we have identified a number of differentially expressed proteins, three significant signaling pathways associated with CaP radioresistance using label-free LC-MS/MS. In addition, the key pathway proteins identified were validated in CaP-RR cell lines and PC-3RR-luc tumor xenografts. Furthermore, targeting ALDOA combined with radiation could increase radiosensitivity in CaP-RR cells. Our findings indicate that controlling the identified proteins or signaling pathways in addition to RT might greatly improve CaP radiosensitivity, overcome radioresistance and become part of a novel therapeutic treatment regimen.

## Materials and Methods

### Antibodies

Antibodies were obtained from different sources. The detailed information and conditions for all antibodies are listed in Table 3. All experimental protocols in the study were approved by the St George and Sutherland Clinical School, UNSW and carried out in accordance with UNSW guidelines and regulations.

### Cell line and cell culture

Non-irradiated and matched parental CaP (control) cell lines (PC-3, DU145 and LNCaP) were obtained from American Type Culture Collection (ATCC) (Rockville, MD, USA). Three CaP-RR cell lines (PC-3RR, DU145RR and LNCaPRR) were developed and confirmed for radioresistance in our previous study^13^. PC-3-luc and PC-3RR-luc cell lines were established using the published method^44^. All cell lines used in this study were cultured in RPMI-1640 medium supplemented with 10% heated-inactivated fetal bovine serum (FBS), 50 U/mL of penicillin and 50 μg/mL of streptomycin. All cell lines were maintained in a humidified incubator at 37 °C and 5% CO_2_. The cells with 80–90% confluence were rinsed twice with Dulbecco’s phosphate-buffered saline (DPBS) (pH7.2), detached with 0.25% trypsin/0.05% EDTA at 37 °C, collected by centrifuge and resuspended in medium and followed by experiments.

### Proliferation assay

CaP and CaP-RR cell lines were plated in triplicate at 2 × 10^4^cells/well in 6-well plates with 3 mL medium and treated according to the same protocol as described in cell line and cell culture (see above). The cell numbers of each well were counted by a hemocytometer in the following consecutive 7 days. Each experiment was repeated three times (n = 3).

### Protein extraction

2 × 10^6^ CaP and CaP-RR cells were harvested into cold PBS by a scraper, following three times wash by PBS. All cells were lysed in lysis buffer (50 mM Tris-HCL (PH 8.0), 150 mM sodium chloride (NaCl), 0.1% SDS, 10 mM sodium fluoride (NaF), 1 mM sodium orthovanadate (Na3VO4), 0.5% sodium deoxycholate, 1% Triton X-100 and 1/12 (v/v) protease inhibitor cocktail) (Sigma-Aldrich Pty Ltd, Castle Hills, NSW, Australia). The lysates were shaken by vortex for 15 min and centrifuged at 13000 rpm for 15 min at 4 °C. The supernatants were collected and ready to precipitate.

### Acetone precipitation

According to our published paper^46^, the protein samples from different cell lines were added to 4 times sample volume of ice-cold (−20 °C) acetone in acetone-compatible tubes, mixed, and incubated for 60 min at −20 °C and then centrifuged (11,000× g) at 4 °C for 10 min. The supernatants were decanted and residual acetone was evaporated from the uncapped tube at room temperature for 30 min. The protein concentrations were determined by a BCA assay kit (Thermo Scientific, USA).

### Protein clean up and digestion

Each protein sample (100 μg) was digested with trypsin (12.5ng/μL trypsin proteomic grade, Sigma-Aldrich, St Louis, MO, USA) in an enzyme:protein ratio of 1:100 (w/w) and incubated at 37 °C overnight (o/n). Five microliter (μL) formic acid (Fluka) was mixed with each sample and centrifuged at 11,000× g for 10 min. The supernatants were dried (Speed Vac Pluc) for 30 min and the pellets were solubilised in 20 μL 0.5% formic acid and loaded into a C18 Stage Tip prepared as the manufacturer’s instructions. The tips were washed with 0.5% formic acid before being eluted using 10 μL 80% ACN and 0.1% formic and dried in a speedVac for 10 min. Samples were resuspended in 10 μL 0.1% formic acid ready for LC-MS/MS.

### LC-MS/MS analysis

LC-MS/MS analysis was carried out for CaP and CaP-RR samples. Digested peptides were reconstituted in 10 μL 0.1% formic acid and separated by nano-LC using an Ultimate 3000 HPLC and autosampler (Dionex, Amsterdam, Netherlands). The sample (0.2 μL) was loaded onto a micro C18 pre-column (300 μm × 5 mm, Dionex, Scoresby, VIC, Australia) with Buffer A (98% H_2_O, 2% CH_3_CN, 0.1% TFA) at 10 μL/min. After washing, the pre-column was switched (Valco 10 port valve, Dionex) into line with a fritless nano column (75 μm i.d. × 15 cm) containing reverse phase C18 media (3 μm, 200 Å Magic, Michrom Bioresoures). Peptides were eluted using a linear gradient of Buffer A to Buffer B (98% CH_3_CH, 2% H_2_O, 0.1% formic acid) at 0.25 μL/min over 60 min. High voltage (2000 V) was applied to low volume tee (Upchurch Scientific, Oak Harbor, WA, USA) and the column tip positioned 0.5 cm from the heated capillary (*T* = 280 °C) of an Orbitrap Velos (Thermo Electron, Bremen, Germany) mass spectrometer. Positive ions were generated by electrospray and the Orbitrap was operated in a data-dependent acquisition (DDA) mode. A survey scan 350–1750 *m*/*z* was acquired in the Orbitrap (Resolution = 30000 at 400 *m*/*z*, with an accumulation target value of 1000000 ions) with lockmass enabled. Up to the 10 most abundant ions (>5000 counts) with charge states +2 to +4 were sequentially isolated and fragmented within the linear ion trap using collisionally induced dissociation with an activation *q* = 0.25 and activation time of 30 ms at a target value of 30000 ions. The *m/z* ratios selected for MS/MS were dynamically excluded for 30 s^46^.

### Progenesis analysis

MS peak intensities were analyzed using Progenesis QI data analysis software v4 (Waters). Ion feature matching was achieved by aligning consistent ion m/z and retention times, normalized against total intensity (sample specific log-scale abundance ratio scaling factor), and compared between groups by one-way analysis of variance (ANOVA, *p* ≤ 0.05 for statistical significance). The *p*-value uses the mean difference, the variance, and also the sample size. Type I errors were controlled by False Discovery Rate (FDR) with *q* value significance set at 0.01^47^,^48^. This highlights that 1% of all significant tests will result in a false positive result. Results are reported as mean ± SD (normalized ion intensity score).

### Protein dataset

Peak lists of proteins were generated using Mascot Daemon/extract_msn (Matrix Science, Thermo, London, UK) using the default parameters, and submitted to Mascot 2.1 (Matrix Science). All MS/MS spectra of differentiating peptides were searched against human non-redundant NCBInr database using the Mascot search program (Matrix Science, London, UK, www.matrixscience.com) for protein identification with the following criteria: (1) species, Homo sapiens; (2) allowed one missed cleavage; (3) variable modifications, Oxidation (M), Phospho (ST) and Phospho (Y); (4) peptide tolerance, ±6 ppm; (5) MS/MS tolerance, ± 0.6 Da; (6) peptide +2, +3 and +4; and (7) enzyme specificity, none. The results were imported into Progenesis LC-MS software and peptides were considered to be confidently identified when matches had a high ion score >20 and peptides were assigned to a protein.

### Ingenuity pathways analysis

IPA (Ingenuity Systems http://www.ingenuity.com) is a web-based software application tool which is designed to extract biological information from large protein lists, gain a high-level overview of the general biology and construct possible protein networks that are associated with proteomics data. IPA Canonical Pathways Analysis tool was used to identify the signaling and metabolic pathways associated with the database.

### Western blotting analysis

Protein expression levels in CaP and CaP-RR cells were determined by western blotting as previously described^13^. After loading proteins, different primary antibodies were incubated at 4 °C (Table 3), followed by an incubation with horseradish peroxidase (HRP)-conjugated secondary antibodies (goat anti-mouse, goat anti-rabbit appropriate antibodies for the host species of primary antibody) (1:2000 dilution). Immunoreactive bands were detected using enhanced chemiluminescence (ECL) substrate (Pierce Chemical Co, Rockford, USA), and imaged using the ImageQuant LAS4000 system (GE Health care, USA). To confirm equal loading of protein lysates, membranes were stripped (Restore Western Blot Stripping Buffer, Pierce) and re-probed using a mouse anti-β-tubulin monoclonal antibody (MAb), then processed as above.

### Subcutaneous (s.c) CaP xenograft animal models

Seven weeks male NOD/SCID mice (Animal Resources Centre, Western Australia) were housed under specific pathogen-free conditions in facilities approved by the University of New South Wales (UNSW) Animal Care and Ethics Committee (ACEC) and manipulations were performed in laminar flow cabinets. All animal studies were performed in accordance with the ACEC guidelines and regulations. Mice were kept at least 1 week before experimental manipulation. All mice remained healthy and active during the experiment. The ACEC specifically approved this study (approval ID: 13/118B). The s.c CaP model was established following our published method^49^. Briefly, cultured PC-3-luc and PC-3RR-luc cells (2 × 10^6^/injection) in 100 μL DPBS were implanted subcutaneously in the right rear flank region of the mouse (n = 10 mice/per group). Tumor progression was documented once weekly by bioluminescence imaging (BLI) for up to 8 weeks using the published method^50^. At the end of experiment (8 weeks post cell inoculation), fresh CaP xenografts were removed and formalin fixed for histological examination and IHC.

### Irradiation

When tumor volumes reached to approximately 70 mm^3^, mice were anaesthetized with a mixture of 45 mg/kg Ketamine (Hospira Australia Pty Ltd, VIC Australia) and 4.5 mg/kg Xylazine (Troy Laboratories Pty Ltd, NSW Australia) and restricted in a lead container. After being appropriately shielded using a lead cover, the lower body of mouse was exposed to 6 Gy fractionated radiation (2 Gy/per day for 3 every other days) by RT instrument (X-RAD 320 biological irradiator, CT, USA) at Biological Resources Imaging Laboratory, UNSW.

### Immunohistochemistry

Standard immunoperoxidase procedures were used for CaP animal xenograft tissues using our published method^51^. Negative controls were treated identically with primary antibody omitted.

### siRNA transfection

ALDOA gene was knocked down by 30 μM ALDOA-siRNA or scr-siRNA (Applied Biosystems Pty a break here Ltd Australia, Melbourne, VIC, Australia) in PC-3RR (androgen non-responsive) and LNCaPRR (androgen responsive) cells respectively, using LipofactAMINE 2000 (Invitrogen, VIC, Australia) following our published method^24^. The optimized incubation time for each cell line with ALDOA-siRNA or scr-siRNA was 72 h, which was determined by western blotting.

### Clonogenic assay

The clonogenic assay was performed as our previously published method^13^. Briefly, PC-3RR and LNCaPRR cells were treated with scr-siRNA, 6 Gy RT, ALDOA-siRNA, or combination with ALDOA-siRNA and 6 Gy RT. 1500 cells from different treated groups were seeded in 10 cm dishes and cultured for 14 days until the colonies were large enough to be observed. The positive colonies, defined as groups of >50 cells, were scored manually using Olympus INT-2 inverted microscope (Tokyo, Japan). The average numbers of colonies were plotted (mean ± SD, n = 3).

### Detection of apoptosis

PC-3RR and LNCaPRR CaP cells were treated with scr-siRNA, 6 Gy RT, ALDOA-siRNA or combination of ALDOA-siRNA and 6 Gy RT, followed by staining with the DNA-binding agents AO/EB (Sigma-Aldrich Pty Ltd) using our previously published method^13^. The results were examined with a confocal microscope (FV300/FV500 Olympus). Apoptotic cells were characterized by morphology including nuclear condensation and fragmentation.

### Assessment of immunostaining

Staining intensity (0–3) in CaP cell lines and animal xenografts were assessed using a confocal microscope (FV300/FV500 Olympus) or a light microscopy (Leica, Germany), respectively. The criteria used for assessment were as previously reported^51^, where: 0 (negative, <25%); 1 (weak, 25–50%); 2(moderate, 50–70%); 3 (strong, >75%) of the tumor cells stained. Evaluation of tissue staining was performed independently by three experienced observers (LC, JH and YL). All specimens were scored blind and an average of grades was taken finally.

### Statistical analysis

All experiments were performed at least three times (n = 3). As control cell lines, three samples of each cell line were employed. All numerical data were expressed as the average of the values obtained, and the SD was calculated. Data from different groups were compared using the two-tailed t-test. ANOVA was used in progenesis analysis. All *p* values were 2-sided. The statistical analysis of immunostaining intensity in animal xenografts was performed as described in our previous publication^52^. *p* < 0.05 was considered significant. All numerical statistical analyses were performed using the GraphPad Prism 6.00 package (GraphPad, San Diego CA, USA).

## Additional Information

**How to cite this article:** Chang, L. *et al*. Identification of protein biomarkers and signaling pathways associated with prostate cancer radioresistance using label-free LC-MS/MS proteomic approach. *Sci. Rep.* **7**, 41834; doi: 10.1038/srep41834 (2017).

**Publisher's note:** Springer Nature remains neutral with regard to jurisdictional claims in published maps and institutional affiliations.

## Supplementary Material

Supplementary Information

## Figures and Tables

**Table 1:** Nineteen proteins overlapped among three paired cell lines and involved in CaP metastasis, progression, signaling pathways and radioresistance.

Gene Symbol	Protein Name
YWHAE	14-3-3 protein epsilon
YWHAG	14-3-3 protein gamma
AHNAK	AHNAK nucleoprotein
ALDOA	aldolase A, fructose-bisphosphate
AHSG	alpha-2-HS-glycoprotein precursor
ATP5H	ATP synthase subunit d, mitochondrial
CANX	calnexin
EIF4B	eukaryotic translation initiation factor 4B
FABP5	Fatty acid-binding protein, epidermal
HSPD1	heat shock 60 kDa protein 1 (chaperonin)
HINT1	histidine triad nucleotide binding protein 1
HIST1H2BC	histone cluster 1, H2bc-like
EIF4H	KIAA0038 (eukaryotic translation initiation factor 4H)
LRRC59	leucine rich repeat containing 59
MCCC1	methylcrotonoyl-CoA carboxylase subunit alpha, mitochondrial
PRDX6	peroxiredoxin 6
PABPC1L	polyadenylate-binding protein 1-like
RPS21	ribosomal protein S21
VIM	Vimentin

**Table 2:** The selected important common alterations of “disease and function” associated with CaP radioresistance in three paired CaP cell lines.

The paired CaP cell lines	Invasion of prostate cancer cell lines		Proliferation of tumor cell lines		Migration of tumor cell lines		Metastasis	
	*p-value*	Molecules number	*p-value*	Molecules number	*p-value*	Molecules number	*p-value*	Molecules number
PC-3 vs PC-3RR	4.62E-05	6	1.23E-04	28	4.41E-04	15	5.82E-04	16
DU145 vs DU145RR	1.60E-04	6	1.20E-05	35	1.69E-04	18	3.38E-05	21
LNCaP vs LNCaPRR	9.60E-05	6	1.65E-04	17	1.65E-04	17	8.67E-05	19

Notes: The cutting point for the pathway selection is E-04. The invasion of CaP lines, proliferation of tumor cell lines, migration of tumor cell lines and metastasis are closely associated with CaP radioresistance.

**Table 3:** Antibodies used for WB and IHC.

Antibody	Source	Type	Dilution for WB and IHC	Incubation time (min)	Temperature
Rabbit anti-human p-Akt	Abcam	PAb	1:1000 (WB) 1:100 (IHC)	O/N	4 °C
Rabbit anti-human p-mTOR	Abcam	MAb	1:1000 (WB) 1:50 (IHC)	O/N	4 °C
Rabbit anti-human p-4EBP1	Cell Signaling	MAb	1:1000 (WB) 1:1200 (IHC)	O/N	4 °C
Rabbit anti-human MCT1	Santa Cruz Biotechnology	PAb	1:1000 (WB) 1:50 (IHC)	O/N	4 °C
Rabbit anti-human MCT4	Santa Cruz Biotechnology	PAb	1:1000 (WB) 1:50 (IHC)	O/N	4 °C
Rabbit anti-human CD147	Invitrogen	PAb	1:1000 (WB) 1:25 (IHC)	O/N	4 °C
Mouse anti-human VEGF [VG-1]	Abcam	MAb	1:1000 (WB) 1:50 (IHC)	O/N	4 °C
Rabbit anti-human VEGF Receptor 2	Abcam	PAb	1:1000 (WB) 1:250 (IHC)	O/N	4 °C
Rabbit anti-human ALDOA	Abcam	PAb	1:500 (WB) 1: 50 (IHC)	O/N	4 °C
Mouse anti-human β-tubulin	Sigma–Aldrich	MAb	1:5000 (WB)	O/N	4 °C
Goat anti-rabbit lgG-HRP	Santa Cruz Biotechnology	lgG	1:5000 (WB)	45	rt
Goat anti-mouse lgG-HRP	Santa Cruz Biotechnology	lgG	1:5000 (WB)	45	rt
Goat anti-rabbit immunoglobulins/HRP	Dako Pty.Ltd	PAb	1:100 (IHC)	45	rt
Rabbit anti-mouse immunoglobulins/HRP	Dako Pty.Ltd	PAb	1:100 (IHC)	45	rt

Abbreviations: IHC: immunohistochemistry; MAb: monoclonal antibody; O/N: overnight; PAb: polyclonal antibody; rt: room temperature; WB: western blotting.
