# Supplementary material for: Identification of protein biomarkers and signaling pathways associated with prostate cancer radioresistance using label-free LC-MS/MS proteomic approach
Source: Sci Rep. 2017 Feb 22;7:41834. doi: 10.1038/srep41834 (PMC5320484; doi:10.1038/srep41834)
Supplement: Supplementary Information [file srep41834-s1.pdf]

Supplementary Information

Identification of protein biomarkers and signaling pathways associated with prostate cancer radioresistance using label-free LC-MS/MS proteomic approach

Lei Chang[1,2,3], Jie Ni[1,2], Julia Beretov[1,2,4], Valerie C. Wasinger[5,6], Jingli Hao[1,2], Joseph Bucci[1,2], David Malouf[7], David Gillatt[7,8], Peter H, Graham[1,2], Yong Li[1,2]

[1]Cancer Care Centre, St George Hospital, Kogarah, NSW 2217, Australia

[2]St George and Sutherland Clinical School, Faculty of Medicine, UNSW, Kensington, NSW 2052, Australia

[3]Department of Obstetrics and Gynecology, The First Affiliated Hospital of Zhengzhou University, Zhengzhou, Henan 450052, China

[4]SEALS, Anatomical Pathology, St George Hospital, Kogarah, NSW2217, Australia

[5]Bioanalytical Mass Spectrometry facility, Mark Wainwright Analytical Centre, UNSW, Kensington, NSW 2052, Australia

[6]School of Medical Science, UNSW, Kensington, NSW 2052, Australia

[7]Department of Urology, St George Hospital, Kogarah, NSW 2217, Australia

[8]Australian School of Advanced Medicine, Macquarie University, NSW 2109, Australia

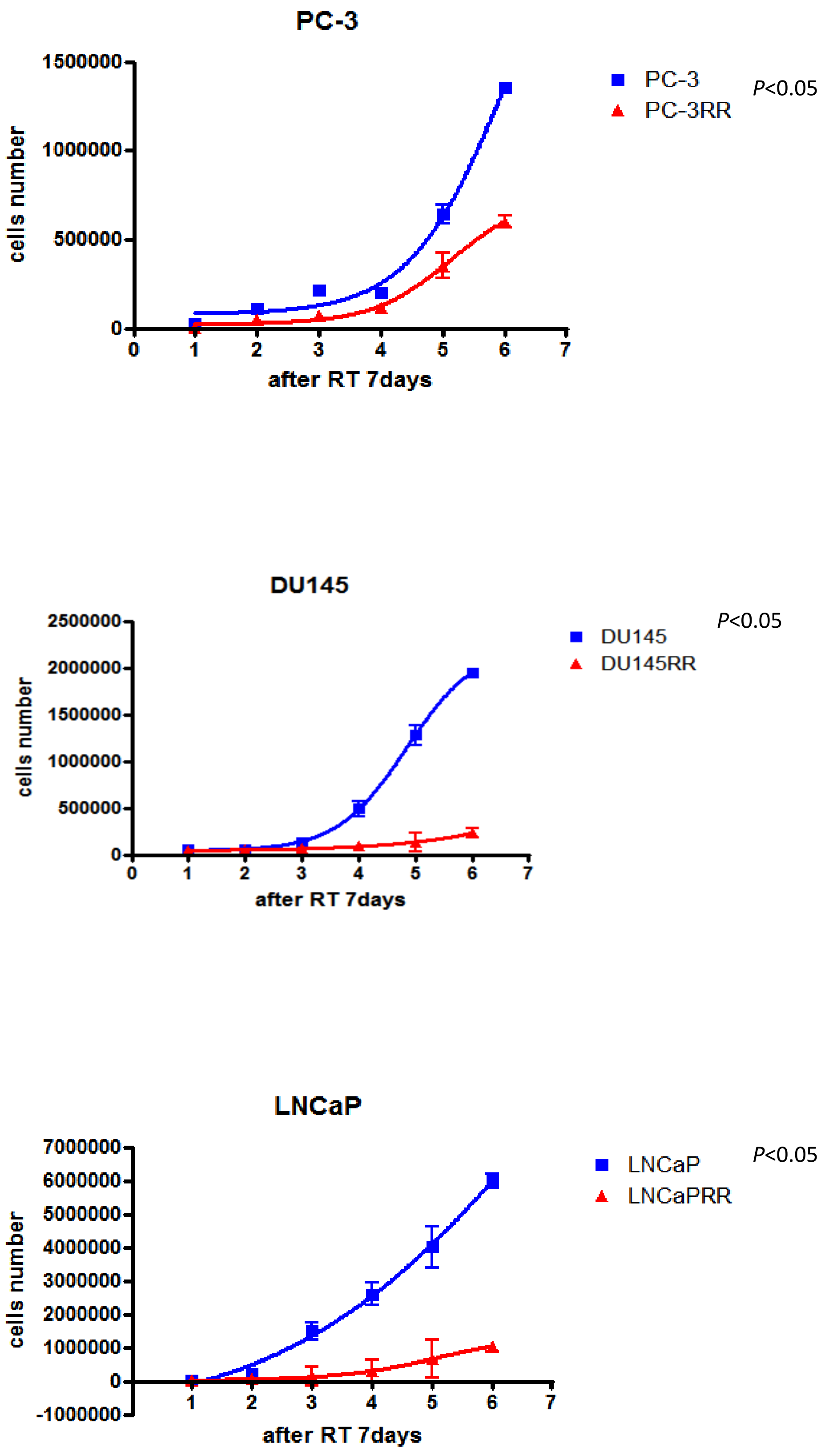

Fig.S1. Proliferationin CaP-RR and CaP-control cells. Proliferation rate in CaP-RR cells was significantly reduced compared with that in CaP-control cells after RT in the first 7 days (P<0.05).

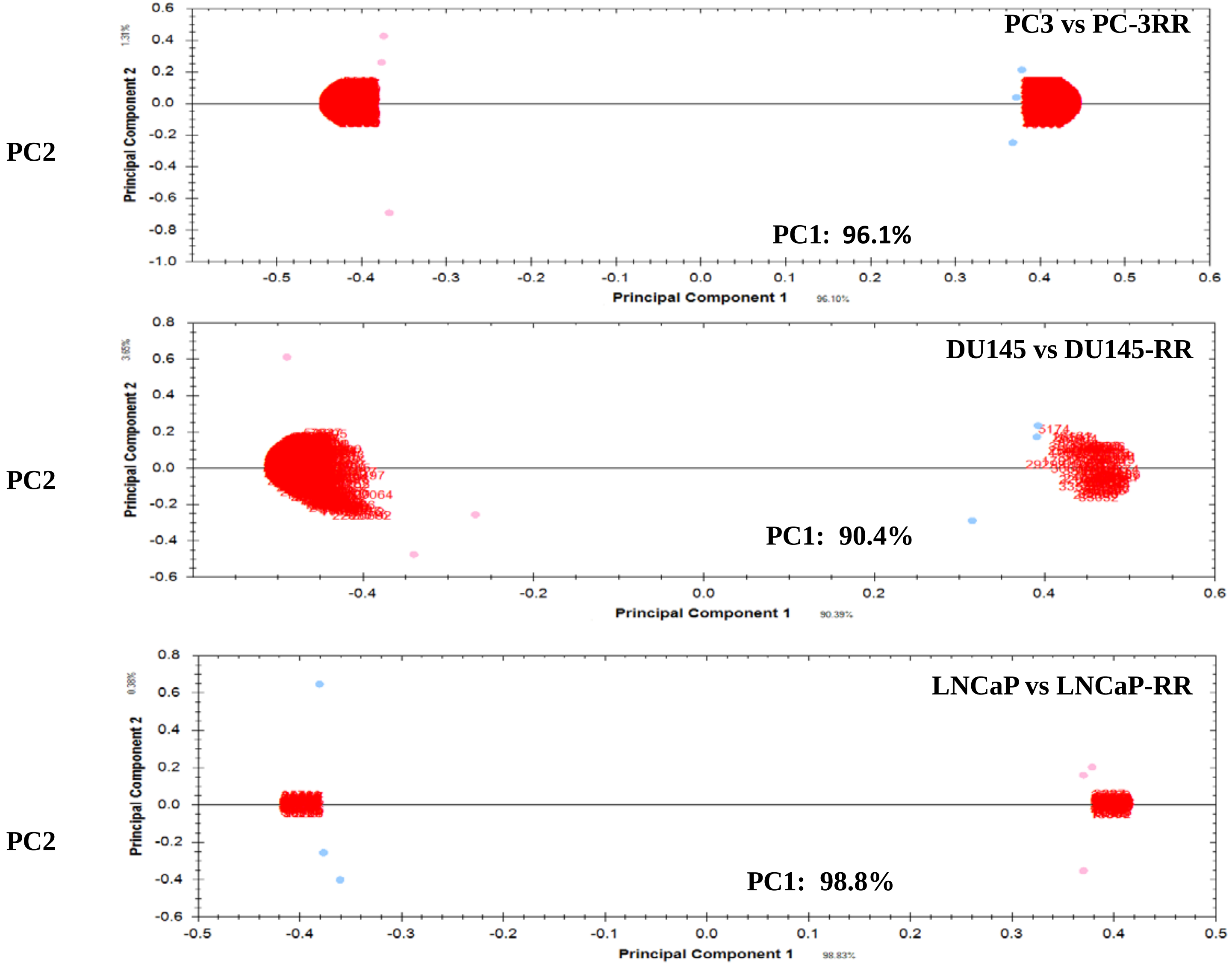

Figure S2. **Identification of protein difference between CaP and CaP-RR cells**. A typical graph is shown the separation of CaP cells (PC-3, DU145 and LNCaP with gray dots) and CaP-RR cells (PC-3RR, DU145RR and LNCaPRR with blue dots) from each other in the Progenesis LC-MS principal component analysis. 96% difference was found for the grouping of PC-3 and PC-3RR; 90% difference was found for the grouping of DU145 and DU145RR; 98% difference was found for the grouping of LNCaP and LNCaPRR.

Table S1. Identified differentially expressed proteins between PC-3 and PC-3RR cell lines

Accession	Peptides	Score	Anova (p)*	Fold	Entrez Gene Name	Average Normalised Abundances	
						PC3	PC3RR
gi|281337712	2	43.95	2.66E-03	1.54	hypothetical protein PANDA_004043	1.98E+04	3.05E+04
gi|61743961	22 (2)	806.68	2.99E-03	1.59	AHNAK nucleoprotein isoform 1	1.18E+04	1.87E+04
gi|395855382	1	42.45	3.97E-03	1.66	methylcrotonoyl-CoA carboxylase subunit alpha, mitochondrial	4076.87	6775.68
gi|354474959	2 (1)	53.38	8.87E-04	1.72	testis-expressed sequence 15 protein-like	1921.36	3306.03
gi|1407651	2 (1)	54.36	1.39E-03	1.75	Lasp-1, partial	4566.75	7986.56
gi|27370132	2 (1)	45.2	5.37E-04	1.78	tetratricopeptide repeat protein 12	1.81E+04	3.21E+04
gi|395858241	1	34.33	1.51E-03	1.81	nuclear autoantigenic sperm protein	3.83E+05	6.92E+05
gi|55291	2	71.67	1.29E-03	2	vimentin	4179.68	8339.64
gi|904132	2	87.2	3.85E-04	2.01	S-adenosyl-L-homocysteine hydrolase	7875.77	1.58E+04
gi|123246553	3	128.1	3.70E-03	2.15	Type 1 glutamine amidotransferase (GATase1)-like domain found in Human DJ-1	6301.94	1.35E+04
gi|6754706	1	50.31	1.46E-04	2.17	MARCKS-related protein	2492.07	5409.39
gi|29801	1	29.46	2.15E-03	2.2	CD44E (epithelial form)	813.12	1787.41
gi|24181958	2 (1)	45.26	1.86E-03	2.25	AMP-activated protein kinase gamma 3 subunit short form	2217.87	4985.32
gi|355557675	1	26.43	5.26E-04	2.3	hypothetical protein EGK_00382, partial	8014.76	1.84E+04
gi|395541072	2	46.38	3.36E-04	2.3	rap guanine nucleotide exchange factor 3	1.06E+04	2.44E+04
gi|119606405	2 (1)	53.75	7.23E-04	2.51	hCG2041963 [Homo sapiens]	1286.89	3229.33
gi|395515688	1	38.9	2.91E-03	2.55	uncharacterized protein LOC100931991	1403.79	3580.54
gi|73969393	2 (1)	47.05	4.38E-03	2.58	NADH dehydrogenase [ubiquinone] 1 alpha subcomplex subunit 6	533.8	1379.24
gi|390475864	1	27.26	4.92E-05	2.62	riboflavin transporter 3	4892.2	1.28E+04
gi|332254193	13 (4)	638.94	4.88E-05	2.68	heat shock protein HSP 90-alpha-like isoform 1	4.80E+04	1.28E+05

gi|55451	3	184.1	1.38E-04	2.69	Y box-binbing protein	2.56E+04	6.90E+04
gi|348569172	2 (1)	41.98	3.96E-04	2.72	cGMP-inhibited 3',5'-cyclic phosphodiesterase A	1.29E+04	3.51E+04
gi|344271638	1	31.2	7.48E-04	2.75	sperm-associated antigen 8-like	1.25E+04	3.45E+04
gi|355750061	2 (1)	51.39	8.36E-04	2.81	Monogenic audiogenic seizure susceptibility protein 1-like protein, partial [Macaca fascicularis]	624.18	1754.51
gi|27806751	5	183.23	5.54E-06	2.86	alpha-2-HS-glycoprotein precursor	1.18E+04	3.38E+04
gi|4506669	7 (2)	407.86	3.89E-03	2.86	60S acidic ribosomal protein P1 isoform 1	8357.28	2.39E+04
gi|6755763	4 (3)	278.21	6.45E-05	2.91	THO complex subunit 4	1.39E+04	4.06E+04
gi|395511400	4	280.73	6.03E-04	2.95	tubulin alpha-1C chain-like	1.91E+04	5.63E+04
gi|291403224	2 (1)	48.8	1.64E-04	3.02	cancer susceptibility candidate 5	1.38E+04	4.16E+04
gi|332858657	2 (1)	43.4	3.23E-03	3.08	nuclear receptor coactivator 3-like	4309.42	1.33E+04
gi|334311748	1	31.6	1.76E-03	3.09	Wilms tumor protein 1-interacting protein-like	4718.54	1.46E+04
gi|355565120	17 (10)	743.04	6.94E-06	3.1	hypothetical protein EGK_04718	8.10E+04	2.61E+04
gi|5802974	1	94.77	4.50E-04	3.1	thioredoxin-dependent peroxide reductase, mitochondrial isoform a precursor	1891.49	5857.41
gi|4826898	11 (9)	440.14	1.11E-05	3.14	profilin-1	1.25E+05	4.00E+04
gi|4758304	6	461.44	9.83E-05	3.16	protein disulfide-isomerase A4 precursor	6.58E+04	2.08E+04
gi|109139213	1	97.02	6.95E-05	3.24	60S ribosomal protein L23a-like, partial	1.82E+04	5.88E+04
gi|351704878	2	50.09	5.23E-05	3.25	Neuroblastoma-amplified sequence, partial	3264.69	1.06E+04
gi|119590497	3	99.2	4.94E-05	3.26	fumarate hydratase	4241.51	1.38E+04
gi|31645	27 (25)	1220.22	8.40E-05	3.3	glyceraldehyde-3-phosphate dehydrogenase	2.47E+05	7.48E+04
gi|395818357	2 (1)	127.17	3.88E-03	3.31	uncharacterized protein LOC100953720	5737.26	1.90E+04
gi|31092	1	31.44	1.48E-03	3.38	eukaryotic translation elongation factor 1 alpha 1	6418.92	2.17E+04
gi|351695030	3	131.87	2.71E-04	3.46	Heterogeneous nuclear ribonucleoprotein D0, partial	1.32E+04	3813.56
gi|355709594	1	35.3	1.45E-05	3.47	protocadherin gamma subfamily B, 6	7990.97	2301.95
gi|58865712	1	29.93	1.85E-04	3.48	ribosomal RNA processing protein 1 homolog A	5715.39	1641.51
gi|2145149	2 (1)	51.11	4.13E-05	3.61	treacle	3244.56	1.17E+04
gi|4504523	29 (22)	1361.96	1.28E-05	3.61	10 kDa heat shock protein, mitochondrial	4.61E+05	1.28E+05

gi|395853404	7	310.81	2.46E-05	3.62	calnexin	2.22E+04	8.05E+04
gi|190569769	2 (1)	46.16	2.00E-05	3.63	olfactory receptor	2.20E+04	7.98E+04
gi|74185237	1	37.71	1.78E-04	3.67	ribosomal protein L3	8326.77	2271.37
gi|344241128	1	29.69	1.44E-04	3.74	HAUS augmin-like complex subunit 2	3.30E+04	8824.05
gi|392353643	4 (1)	176.28	3.06E-03	3.82	tRNA wybutosine-synthesizing protein 5-like	3.11E+04	8129.41
gi|4504445	8	442.13	1.42E-04	3.82	heterogeneous nuclear ribonucleoprotein A1 isoform a	1.96E+04	7.49E+04
gi|33877030	2 (1)	89.18	2.33E-03	3.83	HNRPCL1 protein, partial	1457.43	380.76
gi|345306000	1	59.9	1.21E-05	3.85	DNA-binding protein Ikaros-like	5441.79	1411.95
gi|28614	3	153.07	1.90E-05	3.89	aldolase A	5590.72	2.18E+04
gi|194388892	2	135.17	4.18E-05	3.93	eukaryotic translation initiation factor 4B	1.21E+04	3089.65
gi|354471781	3 (2)	108.51	1.38E-04	3.94	eukaryotic translation initiation factor 3 subunit J-like	2755.64	1.09E+04
gi|4506699	6 (4)	222.27	1.40E-05	3.99	40S ribosomal protein S21	3.35E+04	8389.22
gi|11596261	1	55.42	2.81E-03	4.02	ribophorin I/EVI-1 chimeric fusion protein variant	606.88	2440.79
gi|194221900	1	38.48	3.30E-05	4.06	uncharacterized protein C13orf18 homolog	914.24	3715.91
gi|860986	24 (21)	1021.76	2.48E-06	4.09	protein disulfide isomerase	1.35E+05	5.52E+05
gi|7657532	2	117.04	2.13E-04	4.1	protein S100-A6	1.71E+04	4178.07
gi|157822331	2 (1)	45.91	2.52E-03	4.14	fibronectin type III domain containing 3a	2278.44	550.87
gi|13129092	2	137.73	7.08E-05	4.15	transmembrane protein 109	2094.95	8684.6
gi|13507708	2 (1)	44.97	7.71E-06	4.15	vomeronasal 1 receptor 63	1.20E+04	2893.4
gi|1706754	1	48.29	2.53E-04	4.17	Fatty acid-binding protein, epidermal	1799.46	431.14
gi|89574131	1	71.75	2.45E-04	4.19	mitochondrial malate dehydrogenase 2, NAD	2423.24	1.01E+04
gi|238427	2 (1)	91.97	1.01E-03	4.21	Porin 31HM	449.51	1891.34
gi|4506715	1	48.45	2.44E-05	4.23	40S ribosomal protein S28	1.96E+04	4642.93
gi|114051794	2 (1)	43.29	2.37E-05	4.24	transketolase-like protein 1	2.21E+04	5207.49
gi|194378142	14 (4)	699.58	3.15E-06	4.26	heat shock protein 90kDa alpha (cytosolic), class B member 1	9.01E+04	3.84E+05
gi|7657369	1	64.91	5.21E-07	4.26	NADH dehydrogenase [ubiquinone] 1 alpha subcomplex	4182.55	981.06

					subunit 8		
gi|4502565	1	60.8	2.85E-05	4.27	calpain small subunit 1	5495.11	1286.31
gi|194389558	2 (1)	41.84	8.93E-04	4.29	polypyrimidine tract binding protein 2	1.20E+04	2805
gi|354469079	1	33.42	9.32E-05	4.34	coiled-coil domain-containing protein 57-like	1.38E+05	3.18E+04
gi|395517892	3 (1)	70.62	1.11E-03	4.34	uncharacterized protein LOC100915231, partial	584.62	2535.18
gi|116271891	1	29.41	3.45E-04	4.36	glucose-6-phosphate dehydrogenase	3.83E+04	8788.25
gi|108793529	2 (1)	51.95	1.31E-03	4.39	NADH dehydrogenase subunit 5 (mitochondrion)	1775.74	404.77
gi|149055934	2	73.98	2.05E-04	4.44	U1 small nuclear ribonucleoprotein polypeptide A, isoform CRA_d	9226.25	2079.44
gi|351699686	2 (1)	55.76	1.58E-05	4.48	Tetratricopeptide repeat protein 7B, partial	1975.73	8852.33
gi|395849840	1	40.06	5.52E-05	4.51	protein SFI1 homolog	3007.39	1.36E+04
gi|6724088	1	46.88	2.66E-04	4.57	Bcl-2-binding protein BIS	8348.15	1826.16
gi|4506903	1	47.63	8.66E-05	4.58	serine/arginine-rich splicing factor 9	3855.82	841.39
gi|71153782	1	146.29	3.09E-04	4.59	14-3-3 protein gamma	1.49E+04	3237.33
gi|350593272	1	31.44	1.83E-04	4.61	hypothetical protein LOC100518451	9856.09	2139.19
gi|397518404	2 (1)	53.92	6.25E-04	4.63	chondroitin sulfate synthase 3-like	2223.71	480.49
gi|118466037	1	31.54	2.89E-05	4.66	prolipoprotein diacylglyceryl transferase	3027.16	1.41E+04
gi|395541814	2 (1)	47.31	9.30E-05	4.69	protein transport protein Sec24D	4956.63	2.32E+04
gi|351715819	1	24.78	6.63E-05	4.73	Epithelial chloride channel protein, partial	2.40E+04	5077.59
gi|436226	5 (4)	326.4	3.81E-05	4.77	KIAA0038	2.61E+04	5475.67
gi|291402681	3 (2)	76.55	4.13E-04	4.79	kinesin family member 14	1.04E+05	2.17E+04
gi|190198	2	109.94	2.53E-04	4.81	interleukin 27	3833.63	1.84E+04
gi|913159	2 (1)	93.07	1.96E-04	4.83	neuropolypeptide h3	3367.51	697.73
gi|126283564	1	30.06	3.83E-05	4.85	protein FAM177A1-like	3.32E+04	6853.02
gi|296471982	1	67.97	5.80E-06	4.87	tropomyosin 4-like	5640.42	2.74E+04
gi|10047341	1	34.24	8.67E-04	4.93	ectopic P-granules autophagy protein 5 homolog	1096.9	5403.48
gi|74137988	1	35.9	8.83E-05	4.95	clathrin, heavy chain (Hc)	4.55E+04	9197.4
gi|388453823	15	1140.98	4.56E-06	4.97	protein disulfide-isomerase A6 precursor	1.33E+05	6.59E+05

gi|21624607	1	33.68	1.81E-04	4.98	coactosin-like protein	1334.47	268.14
gi|177216	2	72.73	1.80E-03	5.01	4F2 heavy chain antigen	1420.97	7125.01
gi|4502111	1	81.9	6.08E-04	5.07	annexin A7 isoform 1	1969.33	9977.22
gi|169731526	4	139.99	7.11E-05	5.08	over-expressed breast tumor protein ()	2.77E+04	5458.53
gi|1313962	1	55.08	2.27E-04	5.13	mitochondrial ribosomal protein L12	6915.43	3.55E+04
gi|4506901	3	163.39	1.13E-05	5.21	serine/arginine-rich splicing factor	2.69E+04	5162.33
gi|13276231	1	36.9	1.09E-04	5.26	FYVE and coiled-coil domain containing 1	2092.06	1.10E+04
gi|73949314	1	45.11	9.04E-05	5.29	anion exchange protein 4	600.06	3172.58
gi|291387011	2 (1)	42.42	1.43E-04	5.3	glucokinase regulatory protein	4405.63	2.34E+04
gi|358439492	1	32.26	1.29E-04	5.31	vomeronasal 1 receptor ornAnaV1R3158	716.24	3804.22
gi|291395141	3 (1)	74	1.77E-04	5.38	dynein, axonemal, heavy chain 5-like	1711.83	318.23
gi|295842330	2 (1)	87.11	3.73E-04	5.44	splicing factor 1 isoform 6	1724.71	317.21
gi|344238180	6 (3)	292.42	1.52E-05	5.51	Histone H2A type 1	1.00E+04	5.53E+04
gi|384948758	4 (2)	142.82	1.09E-06	5.55	protein SET isoform 2	2226.52	1.24E+04
gi|380816100	19 (16)	1224.01	1.66E-05	5.7	endoplasmin precursor	1.20E+05	6.85E+05
gi|3387922	1	54.05	9.10E-04	5.91	14.3.3 protein	5222.27	883.08
gi|4506455	1	95.88	1.70E-04	6.01	reticulocalbin-1 precursor	6072.46	1010.73
gi|194227772	2 (1)	54.37	2.33E-04	6.02	dystrophin [Equus caballus]	6873.95	1140.94
gi|61743954	37 (10)	1591.27	8.54E-07	6.06	neuroblast differentiation-associated protein AHNAK isoform 1	1.13E+05	1.87E+04
gi|452977	5	260.95	1.68E-05	6.07	fatty acid binding protein FABP-1	8411.38	5.11E+04
gi|51479152	2 (1)	84.33	9.34E-04	6.08	ATP synthase subunit d, mitochondrial isoform b	6677.89	1098.28
gi|335284315	17 (15)	830.08	5.09E-06	6.09	RNA-binding protein FUS-like isoform 2	1.16E+05	1.91E+04
gi|332212293	2 (1)	57.23	1.65E-03	6.13	kinesin-like protein KIF20B isoform 1	4560.64	2.79E+04
gi|332246147	1	32	5.92E-05	6.13	HLA class II histocompatibility antigen, DO alpha chain-like	4373.54	2.68E+04
gi|205854	1	32.83	5.89E-05	6.16	pR-ET2 encoded oncodevelopmental protein (putative); putative, partial	8583.89	1393.26

gi|332248170	1	30.62	3.72E-07	6.17	solute carrier family 26 member 9-like	8302.96	1345.56
gi|57997566	2	43.67	2.43E-04	6.21	hypothetical protein	6681.74	4.15E+04
gi|351705379	1	35	4.76E-05	6.27	Elastin	3476.88	2.18E+04
gi|387016	1	63.75	5.71E-04	6.31	phosphoglycerate mutase, partial	1.12E+04	1772.88
gi|344309898	7	263.3	1.63E-06	6.44	hypothetical protein LOC100654961	2.43E+04	1.57E+05
gi|396431	1	62.08	2.11E-04	6.54	rab GDI alpha	1531.05	233.94
gi|7705877	6 (5)	300.97	1.77E-05	6.67	hematological and neurological expressed 1 protein isoform 1	1.79E+04	1.20E+05
gi|4758638	7	479.01	1.48E-06	6.7	peroxiredoxin-6	4.40E+04	2.95E+05
gi|7705477	1	62.78	5.10E-05	6.71	tRNA methyltransferase 112 homolog	804.23	5399.52
gi|179074	3 (2)	83.73	2.77E-04	6.75	serine/arginine-rich splicing factor 1	1736.54	257.14
gi|297304419	1	29.88	6.96E-05	6.85	t-complex protein 11 homolog isoform 1	6312.28	921.9
gi|62913985	5	353.97	1.78E-06	6.88	NPM1 protein, partial	1.66E+04	1.14E+05
gi|27754771	2 (1)	52	5.96E-06	6.95	protocadherin-1 isoform 1 precursor	8901.39	6.19E+04
gi|10438672	1	54.08	5.89E-05	6.96	collagen beta(1-O)galactosyltransferase 1	1097.28	7637.98
gi|56403715	1	163.81	2.77E-04	6.97	hypothetical protein	4.64E+04	6648.02
gi|344243167	1	34.61	6.53E-04	7.01	Uncharacterized protein C10orf12	2031.93	1.42E+04
gi|194373909	11	521.9	2.67E-06	7.23	prolyl 4-hydroxylase, beta polypeptide	4.64E+04	3.35E+05
gi|517177	1	35.2	4.19E-04	7.29	YAP65	6645.65	911.1
gi|15150811	2	123.28	2.05E-05	7.32	28S ribosomal protein S36, mitochondrial	2070.93	1.52E+04
gi|395510548	2 (1)	44.13	1.02E-04	7.41	leucyl-cystinyl aminopeptidase	370.37	2743.78
gi|344284198	2 (1)	54.5	6.09E-04	7.48	uveal autoantigen with coiled-coil domains and ankyrin repeats-like	7113.39	5.32E+04
gi|194390706	12 (1)	478.45	2.56E-05	7.61	AHNAK nucleoprotein	9.89E+04	1.30E+04
gi|221042312	44 (37)	2523.4	3.52E-06	7.64	heat shock 60kDa protein 1 (chaperonin)	1.23E+05	9.37E+05
gi|157276209	1	40.04	6.59E-05	7.7	apolipoprotein B, partial	1.87E+04	2426.09
gi|6272557	1	58.24	4.59E-04	7.88	ERO1L	387.19	3049.44
gi|4220566	3 (1)	70.52	1.66E-04	7.94	NADH dehydrogenase subunit 5	1.34E+04	1691.81

gi|440306	2 (1)	79.78	1.45E-04	8.27	enhancer protein	2925.3	353.69
gi|16518999	2 (1)	44.49	1.46E-05	8.4	cadherin EGF LAG seven-pass G-type receptor	6012.37	5.05E+04
gi|89574023	8	485.6	3.14E-08	8.44	mitochondrial ATP synthase, H+ transporting F1 complex beta subunit	2.11E+04	1.78E+05
gi|899229	1	55.93	3.02E-05	8.5	thrombospondin-1	1302.52	1.11E+04
gi|291383053	2 (1)	49.89	1.78E-06	8.57	hCG2040047-like	3606.85	3.09E+04
gi|13928824	4 (3)	388.95	1.11E-04	11.6	14-3-3 protein epsilon	4.44E+04	515008
gi|157833780	3 (1)	148.81	8.06E-05	8.74	Chain A, Human Annexin V With Proline Substitution By Thioproline	1.05E+04	1205.98
gi|119621875	14	791.33	6.34E-06	8.75	hCG23783, isoform CRA_a	4.40E+04	3.85E+05
gi|6005717	1	32.62	2.49E-05	8.76	ATP synthase subunit e, mitochondrial	3059.24	2.68E+04
gi|11493524	2	91.32	3.39E-06	8.79	PRO1633	1243.68	1.09E+04
gi|73959921	1	53.66	2.89E-05	8.93	macrophage colony-stimulating factor 1	1861.04	1.66E+04
gi|602278	1	56.85	2.21E-05	8.98	similar to Pacific ray VAT1 protein, Swiss-Prot Accession Number P19333, partial	6879.55	766.32
gi|181969	2	114.73	3.45E-05	9.06	elongation factor 2, partial	2186.9	1.98E+04
gi|291402415	1	35.34	3.18E-06	9.22	putative deoxyribonuclease TATDN3-like isoform 1	1181.72	128.1
gi|179531	1	30.5	1.96E-03	9.25	IgE-binding protein	4462.14	482.35
gi|1905874	1	33.56	3.09E-04	9.32	carboxyl terminal LIM domain protein	2373.46	254.56
gi|332251910	1	56.04	1.67E-03	9.32	60S ribosomal protein L9-like	2585.63	277.52
gi|392334312	2 (1)	56.8	8.85E-04	9.43	40S ribosomal protein S3a-like	496.3	4680.61
gi|148665383	5	230.04	3.04E-05	9.86	transferrin receptor, isoform CRA_b	7773.64	7.66E+04
gi|149716956	2 (1)	71.29	1.39E-04	10.08	hypoxia up-regulated protein 1	222.94	2246.89
gi|164543	1	70.58	1.72E-03	10.25	malate dehydrogenase (EC 1.1.1.37), partial	2038.06	198.89
gi|395517453	1	34.19	4.57E-03	10.25	EH domain-containing protein 1-like	1380.01	134.6
gi|348560297	2 (1)	56.88	5.37E-06	10.28	smith-Magenis syndrome chromosomal region candidate gene 8 protein-like	2140.48	2.20E+04
gi|2055427	1	32.58	8.68E-06	10.46	KSRP	1.30E+04	1237.69

gi|395819066	2 (1)	67.3	3.49E-06	10.48	palmitoyltransferase ZDHHC21	1.39E+04	1328.83
gi|355698116	2 (1)	87.33	3.84E-04	10.55	Lysine-rich CEACAM1 co-isolated protein	1006.72	1.06E+04
gi|19354126	1	29.28	4.62E-07	11.16	Rusc2 protein, partial	1090.21	1.22E+04
gi|4885413	12 (10)	432.71	3.56E-05	11.43	histidine triad nucleotide-binding protein 1	5.82E+04	5091.41
gi|55250278	7	432.29	1.34E-05	11.49	Hnrpa3 protein	4.45E+04	3868.62
gi|13650039	1	80.92	6.63E-04	11.76	polypeptide GalNAc transferase-T2	711.03	8360.5
gi|2780748	5 (4)	191.33	2.12E-04	11.76	hnRNP JKTBP	1.12E+04	952.77
gi|19527026	2 (1)	95.87	1.40E-05	11.82	leucine-rich repeat-containing protein 59	3382.14	286.17
gi|12018310	1	29.44	7.04E-04	11.94	frizzled class receptor 4	2502.25	209.5
gi|380809052	1	35.16	2.19E-04	12.12	male-specific lethal 3 homolog isoform a	1.07E+04	1.30E+05
gi|345324422	1	35.33	9.77E-04	12.34	ATP-binding cassette sub-family A member 5	1875.46	152.03
gi|16716332	1	36.38	1.31E-05	12.43	treacle	2913.61	234.4
gi|309533	1	86.75	1.42E-03	12.59	vinculin	1409.57	111.95
gi|297285693	2 (1)	113.93	7.36E-06	12.66	mesencephalic astrocyte-derived neurotrophic factor-like	3.22E+04	2541.5
gi|4972627	1	43.21	3.90E-03	13.1	caveolin 1	2513.18	191.78
gi|197101633	1	44.94	4.54E-06	13.17	stanniocalcin-2 precursor	454.2	5980.62
gi|33585944	2 (1)	59.13	5.54E-05	13.44	Ptpn21 protein, partial	9716.36	1.31E+05
gi|4557032	7 (3)	440.18	3.23E-05	13.58	L-lactate dehydrogenase B chain	8308.98	1.13E+05
gi|13128992	1	85	5.37E-04	13.85	chromosome 19 open reading frame 43	5970.23	430.97
gi|206580	2 (1)	64.09	3.88E-05	13.85	RBL-NDP kinase 18kDa subunit (p18)	4994.38	360.73
gi|334348257	2 (1)	61.48	1.37E-04	14.05	ATP-dependent DNA helicase Q1	1434.92	102.1
gi|348552548	2 (1)	47.31	3.60E-05	14.2	bifunctional protease/dUTPase-like	3610.42	254.31
gi|344286678	1	37.92	1.72E-03	14.53	cell surface A33 antigen-like	2270.75	156.24
gi|5803165	1	50.76	9.98E-05	14.71	protein transport protein Sec61 subunit beta	508.79	7483.95
gi|228542	3	100.88	1.20E-06	14.74	myosin	7210.1	489.1
gi|1353272	1	37.21	1.11E-04	14.75	DnaJ (Hsp40) homolog, subfamily C, member 3	436.43	6438.14

gi|18479260	1	30.74	5.85E-06	14.95	olfactory receptor MOR223-3	7050.45	1.05E+05
gi|5107168	1	53.17	3.12E-04	15.12	small zinc finger-like protein	5864.06	387.76
gi|17352354	1	45.93	9.16E-05	15.42	apobec-1 binding protein 2	232.93	3592.24
gi|384942632	2	103.2	4.73E-05	15.47	transitional endoplasmic reticulum ATPase	8879.05	1.37E+05
gi|392339502	2 (1)	42.21	8.14E-06	15.48	fibrous sheath-interacting protein 2-like	1726.37	2.67E+04
gi|119594452	5	285	3.35E-05	15.77	glucosidase, alpha; neutral AB	1.31E+04	2.06E+05
gi|332248186	4 (3)	142.51	1.74E-06	15.96	polyadenylate-binding protein 1-like	4056.14	6.47E+04
gi|2493731	1	117.91	9.62E-04	15.99	Clathrin light chain A	973.93	1.56E+04
gi|5174723	2 (1)	78.43	2.80E-07	16.21	mitochondrial import receptor subunit TOM40 homolog	2.90E+04	4.70E+05
gi|354493703	1	43.74	6.86E-06	16.29	helicase-like transcription factor	3502.66	5.71E+04
gi|13878317	2 (1)	46.1	1.68E-03	16.39	AF4/FMR2 family member 1	8444.44	515.23
gi|180233	1	41.6	4.98E-04	16.49	cell adhesion protein (SQM1)	141.03	2326
gi|194376104	8 (1)	359.89	5.88E-06	16.55	heat shock 70kDa protein 1A	3407.59	5.64E+04
gi|395819884	1	30.48	5.52E-05	17.12	DNA replication licensing factor MCM5	7322.65	427.79
gi|194208691	1	34.3	1.56E-05	17.36	tumor necrosis factor receptor type 1-associated DEATH domain protein-like	1011.62	1.76E+04
gi|344308849	1	32.08	2.59E-04	17.94	uncharacterized coiled-coil domain-containing protein KIAA1984-like	507.26	9101.47
gi|395511170	2 (1)	51.78	2.69E-04	18.44	suppression of tumorigenicity 18 protein	2997.39	162.52
gi|1465751	1	44.38	9.39E-04	18.45	VHL binding protein-1, partial	1299.75	70.46
gi|62988641	2	101.64	8.80E-06	18.67	reticulon 4	1.35E+04	2.52E+05
gi|726098	1	154.93	2.03E-05	19.12	glutathione S-transferase-P1c	1.84E+04	961.93
gi|345308480	1	47.13	3.29E-04	19.13	E3 SUMO-protein ligase PIAS1	7399.02	386.82
gi|351715524	1	40.94	8.15E-06	19.17	Apoptosis-enhancing nuclease	799.36	1.53E+04
gi|5031851	1	49.73	1.23E-04	19.98	stathmin isoform a	288.85	5772.55
gi|15808948	1	40.85	8.38E-05	20.51	proliferating cell nuclear antigen	896.73	1.84E+04
gi|194225038	1	21.57	8.92E-06	20.63	exocyst complex component 5-like	2552.26	5.26E+04
gi|338711737	2 (1)	60.19	1.11E-04	20.9	myosin-XV	2.50E+04	1198.11

gi|354476994	1	31.84	1.56E-05	21.55	contactin-associated protein-like 4 isoform 2	3787.18	175.71
gi|90655152	3 (1)	152.46	1.67E-04	21.55	actinin alpha4 isoform	916.72	1.98E+04
gi|130492446	1	34.35	7.50E-06	21.98	lysosomal-associated transmembrane protein 4A	7179.58	1.58E+05
gi|395545687	2 (1)	40.88	4.44E-04	22.69	oligophrenin-1 [Sarcophilus harrisii]	1599.07	70.48
gi|77997786	7 (1)	458.67	4.09E-04	22.95	actin [Canis lupus familiaris]	2867.09	124.92
gi|395854881	1	46.54	5.40E-07	23.12	synaptotagmin-like protein 1	917.88	2.12E+04
gi|395835178	2 (1)	119.71	1.15E-04	23.18	receptor tyrosine-protein kinase erbB-3 isoform 1	643.48	1.49E+04
gi|358412217	2 (1)	62.94	2.02E-04	24.86	uncharacterized protein C12orf40-like	9133.47	367.37
gi|4758152	1	33.18	1.84E-05	24.94	mitochondrial import inner membrane translocase subunit Tim8 A isoform 1	386.87	9650
gi|28202071	2	95.77	2.44E-06	24.96	protein CDV3 isoform a	2.57E+04	1029.7
gi|351702995	2 (1)	52.63	2.74E-05	25.26	Dynein heavy chain 8, axonemal	278.08	7023.92
gi|345790259	1	25.11	2.57E-03	25.54	heat shock protein 105 kDa isoform 1	232.21	5931.66
gi|335306285	2 (1)	56.81	1.97E-04	25.95	cleavage stimulation factor subunit 2-like isoform 2	268.02	6955.3
gi|9910382	3	195.45	9.23E-07	26.15	mitochondrial import receptor subunit TOM22 homolog	9141.13	2.39E+05
gi|119612797	1	75.18	8.18E-05	26.39	signal recognition particle 14kDa (homologous Alu RNA binding protein)	3387.17	8.94E+04
gi|30794310	21	795.45	2.73E-07	26.66	beta-casein precursor	3.57E+04	9.52E+05
gi|576623	2	119.23	3.39E-04	27.07	ESP-2, partial	1.28E+04	471.07
gi|281348207	2 (1)	72.23	7.41E-04	28.16	hypothetical protein PANDA_014880	154.45	4349.47
gi|78557859	1	41.55	8.89E-06	28.18	ribosomal protein L4 [Ovis aries]	4844.5	171.91
gi|291399186	1	61.61	4.23E-04	28.41	adenylyl cyclase-associated protein	4294.3	151.18
gi|149061788	1	30.77	5.78E-06	30.03	rCG47569, isoform CRA_a	288.77	8670.29
gi|344286868	2 (1)	67.9	6.44E-06	30.11	pyruvate kinase isozymes R/L	669.94	2.02E+04
gi|359322549	1	37.95	1.37E-05	30.28	villin-like	502.1	1.52E+04
gi|3912945	1	55.17	1.82E-06	32.05	Proteolipid protein 2	1625.53	5.21E+04
gi|1552328	1	37.76	2.70E-03	33.36	TFG	6158.69	184.62
gi|281339108	1	34.57	7.36E-05	35.52	hypothetical protein PANDA_011978	7753	218.3

gi|225632	5	209.47	2.09E-07	35.65	casein alphaS1	2595.46	9.25E+04
gi|145273622	9	640.56	2.16E-06	38.24	Prohibitin family; SPFH (stomatin, prohibitin, flotillin, and HflK/C) superfamily	1.96E+04	7.49E+05
gi|354498605	1	32.2	1.33E-05	38.63	protein RoBo-1-like	2502.13	9.67E+04
gi|81342470	2	95.13	4.41E-07	38.83	Transketolase, pyrimidine binding domain	398.93	1.55E+04
gi|4504919	2	99.12	1.51E-05	39.6	keratin, type II cytoskeletal 8 isoform 2	204.45	8095.59
gi|297275921	2 (1)	52.71	3.87E-04	40.22	DENN domain-containing protein 1C-like, partial	258.72	1.04E+04
gi|1050527	1	64.76	2.06E-05	40.93	seryl-tRNA synthetase	408.95	1.67E+04
gi|334326776	1	50.39	2.44E-04	41.61	protein Dos-like	155.64	6475.77
gi|281349809	6 (1)	241.42	6.18E-06	41.85	hypothetical protein PANDA_005719	129.52	5421.03
gi|355684742	2 (1)	54.4	4.32E-06	42.03	dopey family member 2	1371.26	32.63
gi|16902046	2 (1)	43.8	5.37E-05	44.08	anti-HLA class II antibody F3.3 kappa light chain variable region	4414.24	100.14
gi|296193285	2 (1)	49.1	1.84E-03	45.45	protocadherin Fat 2	2.24E+04	492.5
gi|119625899	1	50.78	9.51E-04	47.35	phosphoribosylaminoimidazole carboxylase, phosphoribosylaminoimidazole succinocarboxamide synthetase	4930.74	104.13
gi|344286413	1	35.42	2.19E-05	51	protein S100-A7-like	110.83	5652.5
gi|112696	1	82.57	6.73E-04	53.83	D4, zinc and double PHD fingers family 1	6169.26	114.6
gi|1200072	1	31.99	2.68E-03	68.92	keratin 7	26.29	1811.94
gi|1352426	2	122.93	2.53E-04	71.51	Eukaryotic translation initiation factor 1A	1.69E+04	236.31
gi|1488414	1	45.41	1.57E-06	71.97	N8 gene product long isoform, N8L protein	427.98	3.08E+04
gi|119612746	1	30.65	1.52E-03	80.1	hCG37408, isoform CRA_a	113.51	9092.24
gi|109121620	2 (1)	46.62	9.20E-05	80.49	hypothetical protein LOC722509, partial	11.2	901.31
gi|20149993	8 (2)	321.37	1.41E-04	80.85	Chain A, Structure Of Human Trypsin Iv	2.00E+04	246.95
gi|180928	1	57.68	2.88E-07	85.74	core protein II precursor	295.43	2.53E+04
gi|74220095	1	64.13	4.29E-05	91	transketolase	6289.25	69.11

gi|344248195	1	30.06	7.19E-04	94.42	Eukaryotic translation initiation factor 2-alpha kinase 4	163.11	1.54E+04
gi|2232237	1	71.72	9.01E-06	96.72	secretory carrier membrane protein	191.65	1.85E+04
gi|16549111	1	34.91	4.22E-04	98.76	Shippo 1	813.82	8.24
gi|37620220	1	107.04	5.04E-05	101.77	HCCR-binding protein 2	4614.22	45.34
gi|344264156	2 (1)	43.03	5.22E-04	103.3	probable ATP-dependent RNA helicase DDX43-like	172.68	1.78E+04
gi|6753752	1	39.04	3.86E-04	115.52	homeobox protein engrailed-2	189.31	2.19E+04
gi|291395669	5 (1)	218.97	4.63E-04	134.32	histone cluster 1, H2bc-like	33.67	4522.18
gi|281341534	1	42.01	2.42E-04	195.65	hypothetical protein PANDA_009206	30.41	5950.67
gi|119910827	1	31.3	8.16E-05	228.89	sialic acid binding Ig-like lectin 5-like	95.72	2.19E+04
gi|395844012	2 (1)	51.27	1.46E-03	250.49	protein NPAT	59.49	1.49E+04
gi|119585908	1	30.53	6.41E-05	326.71	eukaryotic translation initiation factor 4E member 3	7.69	2512.21
gi|30424641	1	29.1	2.87E-03	344.72	SH2 domain-containing protein 7	1326.34	3.85
gi|344251893	1	30.05	4.08E-03	357.94	Solute carrier organic anion transporter family member 4C1	2262.85	6.32
gi|11139093	1	38.44	1.48E-04	412.95	GrpE-like protein cochaperone	12.45	5140.54
gi|297263815	2 (1)	50.53	1.48E-03	424.76	dynein heavy chain 10, axonemal-like	908.04	2.14
gi|50510489	2 (1)	49.18	1.43E-04	506.58	mKIAA0453 protein	74.03	3.75E+04
gi|124231	1	56.5	1.65E-03	796.61	Eukaryotic translation initiation factor 5A-1	2684.61	3.37
gi|345791651	1	38.66	1.52E-03	817.88	putative ribosomal RNA methyltransferase NOP2	5.45	4454.57
gi|351715907	1	81.04	2.77E-03	892.83	D-tyrosyl-tRNA(Tyr) deacylase 1	6.94	6200.26
gi|9966827	1	38.21	5.48E-04	1151.41	PEST proteolytic signal-containing nuclear protein	1564.02	1.36
gi|306873	2	203.77	1.36E-04	1335.24	high mobility group protein	36.81	4.92E+04
gi|334322442	1	46.34	4.25E-04	2160.65	u3 small nucleolar RNA-associated protein 18 homolog	1.56	3371.21
gi|345320670	2 (1)	43.31	5.76E-04	3689.67	high affinity immunoglobulin gamma Fc receptor I-like	2.44	8988.29
gi|344275348	1	33.29	6.68E-08	Infinity	lymphocyte function-associated antigen 3-like	0	1293.54

gi|5453549	1	53.87	1.05E-08	Infinity	peroxiredoxin-4 precursor	0	2114.02

***** significant expression : $p<0.05$.

Table S2. Identified differentially expressed proteins between DU145 and DU145RR cell lines

Accession	Peptides	Score	Anova (p)*	Fold	Description	Average Normalised Abundances	
						DU145	DU145RR
gi|114145515	6 (5)	378.45	1.09E-03	1.55	serine/threonine-protein kinase N3	7.56E+05	4.89E+05
gi|4505763	11 (10)	779.79	0.01	1.58	phosphoglycerate kinase 1	4.01E+05	2.55E+05
gi|340219	2	111.15	7.22E-03	6.35	vimentin	2.98E+04	46991
gi|432110524	2	95.52	0.02	1.72	Myosin regulatory light polypeptide 9	1.20E+05	2.06E+05
gi|860986	9 (8)	540.10	4.50E-03	1.73	protein disulfide isomerase	4.65E+05	2.70E+05
gi|6755202	2	184.40	0.04	1.74	proteasome subunit beta type-3	1.23E+05	7.05E+04
gi|693937	9 (5)	586.45	5.80E-03	1.80	polyadenylate binding protein II	1.97E+05	1.09E+05
gi|28614	9 (8)	457.77	0.02	1.81	aldolase A, fructose-bisphosphate	2.86E+05	5.17E+05
gi|6912494	3	89.36	0.02	1.88	microtubule-associated protein RP/EB family member 1	4533.11	8538.33
gi|895845	3	152.30	0.01	1.90	p64 CLCP	4.87E+04	9.24E+04
gi|426236743	3 (2)	142.26	3.65E-03	1.95	LOW QUALITY PROTEIN: ADP/ATP translocase 2-like	4.41E+04	8.61E+04
gi|3329498	12 (10)	1012.39	0.01	1.96	heterogenous nuclear ribonucleoprotein A2/B1	5.96E+05	1.17E+06
gi|488565178	4 (3)	135.45	0.01	1.96	splicing factor, proline- and glutamine-rich-like isoform 1	5.48E+04	1.07E+05
gi|296474454	22 (7)	1250.85	0.02	1.98	TPA: heat shock protein HSP 90-beta	2.69E+05	5.33E+05
gi|494619	3 (2)	98.94	0.04	1.98	Chain I, The Refined 2.4 Angstroms X-Ray Crystal Structure Of Recombinant Human Stefin B In Complex With The Cysteine Proteinase Papain: A Novel Type Of Proteinase Inhibitor Interaction	4.70E+04	2.37E+04
gi|431893704	7 (6)	540.44	0.03	2.02	Pyruvate kinase isozyme M1	8.01E+05	3.97E+05
gi|5031857	8 (3)	379.94	0.03	2.02	L-lactate dehydrogenase A chain isoform 1	1.19E+05	5.87E+04

gi|296234804	4 (2)	249.97	8.21E-03	2.07	transcription intermediary factor 1-beta isoform 2	4.76E+04	2.30E+04
gi|703093	3	142.12	0.03	2.07	serine hydroxymethyltransferase, partial	3.01E+04	6.22E+04
gi|507574478	5 (4)	193.10	0.04	2.11	ran-specific GTPase-activating protein	6.79E+04	1.43E+05
gi|38013966	13 (12)	700.12	3.71E-03	2.12	TKT protein	3.21E+05	6.81E+05
gi|504133999	10 (8)	470.95	3.04E-04	2.17	10 kDa heat shock protein, mitochondrial-like	3.00E+05	6.50E+05
gi|126342959	4	206.34	4.96E-04	2.22	proteasome subunit alpha type-2-like	4.15E+04	9.20E+04
gi|197692147	3 (2)	312.68	0.02	2.35	T-complex protein 1 subunit beta	7.09E+04	3.01E+04
gi|3712663	2	55.87	4.49E-03	2.38	DEAD-box protein	9894.54	2.35E+04
gi|426241785	5 (3)	171.85	0.01	2.43	ubiquitin-60S ribosomal protein L40-like	7.45E+04	3.06E+04
gi|528755289	5 (3)	264.91	3.20E-04	2.46	translational activator GCN1	5.17E+04	2.11E+04
gi|4502847	2	208.77	0.01	2.53	cold-inducible RNA-binding protein	8.92E+04	3.53E+04
gi|704416	3	187.54	8.06E-03	2.55	elongation factor Tu	4.62E+04	1.18E+05
gi|332218133	2	144.26	0.02	2.61	prolyl endopeptidase	2.13E+04	8158.60
gi|4503291	2	55.33	0.02	2.68	D-dopachrome decarboxylase	5089.22	1901.05
gi|444523630	4	194.22	0.01	2.85	NSFL1 cofactor p47	1.00E+05	3.50E+04
gi|194211629	5	242.15	6.96E-03	2.90	triosephosphate isomerase	2.37E+05	8.18E+04
gi|2501351	4	194.48	0.02	2.92	Serotransferrin	2.21E+04	6.46E+04
gi|397465809	6	316.50	1.97E-03	3.00	macrophage migration inhibitory factor	8.37E+05	2.79E+05
gi|432109189	3 (1)	99.64	6.85E-04	3.00	Isocitrate dehydrogenase [NADP] cytoplasmic	5279.53	1.59E+04
gi|119604623	4 (1)	194.20	7.26E-03	3.03	protein kinase C substrate 80K-H, isoform CRA_c	4.92E+04	1.62E+04
gi|397464729	9 (7)	585.75	1.76E-03	3.04	peptidyl-prolyl cis-trans isomerase A-like, partial	3.41E+05	1.04E+06
gi|662994	1	42.71	2.33E-03	3.05	GPI-anchored protein p137	9208.40	3023.50
gi|5901922	2	78.85	3.58E-03	3.10	hsp90 co-chaperone Cdc37	1.87E+04	5.79E+04
gi|537218817	3 (1)	114.17	1.57E-03	3.11	myomesin-3	4.51E+04	1.45E+04

gi|35218	2 (1)	127.76	2.75E-03	3.15	unnamed protein product	2459.27	7734.80
gi|444721520	9	804.93	4.96E-03	3.23	Nucleoside diphosphate kinase B	4.91E+05	1.58E+06
gi|470632809	2 (1)	76.81	1.25E-03	3.23	LOW QUALITY PROTEIN: insulin receptor substrate 1-like	1273.40	4109.45
gi|465991859	2 (1)	101.22	2.00E-03	3.24	serine hydroxymethyltransferase, cytosolic isoform 1	8554.65	2643.46
gi|109113948	3	209.04	6.52E-05	3.28	40S ribosomal protein S17-like isoform 2	3.67E+05	1.12E+05
gi|12653493	2 (1)	115.82	6.58E-03	3.28	Brain abundant, membrane attached signal protein 1	1.71E+05	5.62E+05
gi|524930080	6 (1)	347.43	1.31E-03	3.29	polyadenylate-binding protein 1-like	3.44E+04	1.13E+05
gi|537191106	4 (1)	140.09	9.70E-03	3.37	nuclear factor related to kappa-B-binding protein	1.20E+04	4.03E+04
gi|74152226	2	141.86	5.12E-03	3.42	unnamed protein product	1.28E+05	3.75E+04
gi|431910380	9 (1)	341.49	5.67E-03	3.44	Neuroblast differentiation-associated protein AHNAK	1.39E+04	4036.69
gi|20987362	1	121.82	3.90E-03	3.45	TXLNA protein, partial	1.02E+04	2947.67
gi|1535	4 (3)	149.01	0.01	3.50	unnamed protein product	1.35E+04	3851.39
gi|512905160	2	179.78	9.76E-03	3.54	peptidyl-prolyl cis-trans isomerase FKBP3 isoform X1	6.13E+04	1.73E+04
gi|441597562	3 (2)	108.74	1.55E-03	3.60	prefoldin subunit 1	6974.29	2.51E+04
gi|444722654	3 (2)	114.11	1.39E-03	3.60	T-complex protein 1 subunit delta	8.02E+04	2.23E+04
gi|114052298	1	69.34	9.53E-05	3.66	apolipoprotein A-II precursor	1.09E+05	2.98E+04
gi|1373363	2	77.98	1.57E-03	3.70	platelet-activating factor acetylhydrolase isoform Ib beta subunit	3.97E+04	1.07E+04
gi|507984758	1	43.47	2.52E-03	3.73	caspase recruitment domain-containing protein 14	7594.80	2.84E+04
gi|441620457	16 (1)	978.87	4.86E-03	3.74	tubulin alpha-1C chain-like	5440.31	1454.89
gi|426232708	2 (1)	98.19	2.73E-03	3.75	proteasome activator complex subunit 1	4915.35	1.85E+04
gi|495128	3	258.19	2.29E-03	3.75	mCBP	1.63E+05	4.36E+04

gi|4826760	3	189.31	8.04E-04	3.84	heterogeneous nuclear ribonucleoprotein F	2.65E+04	1.02E+05
gi|4502981	2	71.33	0.02	3.85	cytochrome c oxidase subunit 4 isoform 1, mitochondrial precursor	2.03E+04	7.83E+04
gi|344287751	9 (4)	427.11	8.36E-04	3.95	peroxiredoxin-1-like	7.53E+04	2.97E+05
gi|194381968	4 (3)	169.99	1.42E-03	3.97	unnamed protein product	1.04E+04	4.14E+04
gi|181575	1	88.40	7.64E-03	4.01	dihydrolipoamide dehydrogenase precursor	5.08E+04	1.27E+04
gi|57014043	6 (5)	359.96	7.60E-04	4.02	lamin A/C transcript variant 1	3.38E+05	8.40E+04
gi|4758638	5	292.46	1.02E-03	2.47	peroxiredoxin-6	1.51E+05	6.12E+05
gi|27597059	1	47.10	3.50E-04	4.06	dnaJ homolog subfamily C member 9	1.71E+04	4195.41
gi|4755083	1	96.93	3.93E-03	4.06	density regulated protein drp1	8485.75	3.44E+04
gi|17028367	1	38.77	2.80E-03	4.07	Similar to gelsolin (amyloidosis, Finnish type), partial	1689.74	6870.91
gi|190192184	11 (9)	645.26	4.89E-06	4.08	filamin A	2.26E+05	5.53E+04
gi|344249729	2 (1)	125.37	1.11E-03	4.09	Phosphoglycerate kinase 2	1.11E+04	4.56E+04
gi|74007500	12 (4)	670.05	9.22E-04	4.09	moesin isoformX2	1.41E+05	3.43E+04
gi|297689148	3 (1)	161.13	8.06E-03	4.10	L-lactate dehydrogenase A-like 6A	7173.73	2.94E+04
gi|225719953	2 (1)	112.23	8.91E-03	4.11	selenium binding protein 1 (predicted)	2504.55	608.66
gi|14141152	7 (5)	434.01	6.29E-06	4.14	heterogeneous nuclear ribonucleoprotein M isoform a	3.51E+04	1.46E+05
gi|20067392	3 (2)	131.28	1.46E-03	4.14	thioredoxin related protein	3.87E+04	9343.12
gi|528949501	2 (1)	65.73	8.06E-04	4.14	nascent polypeptide-associated complex subunit alpha isoform X1	2.02E+04	4872.54
gi|56554533	1	61.88	7.53E-03	4.21	Chain J, Clathrin D6 Coat	1.40E+04	3318.84
gi|31645	22 (3)	1357.56	0.01	4.22	glyceraldehyde-3-phosphate dehydrogenase	3.30E+04	1.39E+05
gi|332836687	30 (12)	1484.41	4.31E-04	4.23	neuroblast differentiation-associated protein AHNAK isoform	3.45E+05	8.16E+04
gi|395530406	2	71.30	3.57E-03	4.30	uncharacterized protein LOC100927683	4.94E+04	1.15E+04
gi|5729953	5	232.76	2.99E-03	4.37	nuclear migration protein nudC	1.40E+04	6.11E+04
gi|74222288	4 (3)	209.31	9.24E-04	4.40	unnamed protein product	1.75E+05	3.97E+04

gi|7387724	1	33.81	5.60E-05	4.52	3-hydroxyacyl-CoA dehydrogenase type-2	4317.86	1.95E+04
gi|317455099	1	78.90	4.15E-03	4.60	Chain A, Crystal Structure Of Human Nudt5 Complexed With 8-oxo-dgdp And Manganese	4.98E+04	1.08E+04
gi|533183091	2 (1)	101.27	2.78E-04	4.66	RNA-binding motif protein, X chromosome isoform X4	6.57E+04	1.41E+04
gi|119597764	1	61.96	1.46E-04	4.68	hCG2045348	2.12E+04	9.93E+04
gi|444727966	2 (1)	60.19	2.19E-05	4.69	Alpha-aminoadipic semialdehyde dehydrogenase	6990.91	1490.58
gi|19923193	4 (1)	175.51	6.21E-03	4.72	hsc70-interacting protein isoform 1	2658.36	1.26E+04
gi|466049892	2 (1)	98.46	5.24E-04	4.74	cathepsin D	3.38E+04	7144.64
gi|730529	3	154.46	2.82E-05	4.75	RecName: Full=60S ribosomal protein L13	8.76E+04	1.84E+04
gi|148667738	4 (3)	201.17	1.56E-03	4.79	mCG1035528	1.45E+04	6.93E+04
gi|55733515	1	78.72	8.27E-03	4.79	hypothetical protein	3.54E+04	7396.09
gi|119602640	4	248.84	3.73E-03	4.80	eukaryotic translation elongation factor 1 delta (guanine nucleotide exchange protein), isoform CRA_e	3.29E+04	1.58E+05
gi|225029822	1	51.35	2.21E-03	5.02	C-C chemokine receptor 2 transcript variant 2	4.13E+04	8227.25
gi|403290048	2 (1)	119.41	8.19E-03	5.02	drebrin	6171.95	3.10E+04
gi|507662067	2	170.25	6.07E-07	5.03	lupus La protein-like	1.52E+04	7.63E+04
gi|531997376	1	95.08	4.67E-03	5.10	proteasome subunit alpha type-3 isoform X1	1.53E+04	2994.15
gi|537204009	12 (8)	703.80	1.01E-05	5.14	phosphoglycerate mutase 2-like protein	9.97E+05	1.94E+05
gi|81294349	1	87.35	5.84E-04	5.17	DHX9 protein	2.45E+04	4744.29
gi|426386401	1	99.25	1.56E-03	5.22	calponin-2 isoform 4	7.93E+04	1.52E+04
gi|441592865	5	258.68	6.96E-04	5.28	thioredoxin isoform 2	1.31E+05	6.90E+05
gi|431905273	5 (1)	339.66	2.50E-03	5.36	Endoplasmin	2.78E+04	5181.53
gi|7657603	1	41.35	1.26E-03	5.37	heme-binding protein 2	783.16	4209.08

gi|5031512	2 (1)	91.80	5.41E-03	5.39	NSAP1 protein	1.20E+04	6.46E+04
gi|403265302	2 (1)	153.04	2.72E-03	5.40	T-complex protein 1 subunit beta-like	6990.37	1294.28
gi|7023323	1	43.75	2.29E-04	5.40	unnamed protein product	9624.67	1782.31
gi|403292972	3 (1)	219.41	5.65E-03	5.48	alpha-actinin-4	1.06E+05	1.94E+04
gi|432096880	6 (5)	340.59	5.38E-04	5.59	Methylcrotonoyl-CoA carboxylase subunit alpha, mitochondrial	7.56E+05	1.35E+05
gi|347964	1	49.97	8.75E-03	5.60	TARBP-b, partial	573.63	3214.30
gi|4827038	2	84.08	7.19E-04	5.64	tumor protein D52 isoform 3	1.69E+04	9.55E+04
gi|537179976	1	39.14	1.29E-03	5.66	exophilin-5	6.08E+04	3.44E+05
gi|296209622	3	103.70	4.21E-04	5.72	replication protein A 14 kDa subunit-like	1.23E+04	7.02E+04
gi|119625664	9 (1)	644.03	0.01	5.77	annexin A5, isoform CRA_c	5.69E+04	9853.79
gi|514475709	1	35.29	8.60E-04	5.92	vomeronasal type-2 receptor 26-like	9669.32	1632.67
gi|524928321	10 (2)	548.64	7.23E-04	5.95	creatine kinase B-type isoform X1	1.03E+05	1.74E+04
gi|478490666	1	34.84	5.88E-03	5.96	three prime repair exonuclease 1-like isoform 1	3809.65	639.13
gi|343960957	18 (1)	1755.07	8.76E-04	6.00	alpha-enolase	7.00E+04	1.17E+04
gi|154147607	2	90.79	6.32E-03	6.06	calpain-2 catalytic subunit	1.87E+04	3081.60
gi|74007267	2	80.10	1.76E-03	6.06	poly(rC)-binding protein 1-like isoform X2	9.60E+04	1.58E+04
gi|511881761	2 (1)	73.78	2.85E-04	6.12	cytochrome b-c1 complex subunit 1, mitochondrial-like isoform X1	1700.92	1.04E+04
gi|545485343	16 (3)	985.56	4.64E-04	6.16	LOW QUALITY PROTEIN: tubulin beta-4B chain	1.54E+05	2.50E+04
gi|344248192	2 (1)	127.85	5.77E-06	6.17	Signal recognition particle 14 kDa protein	1.44E+05	2.34E+04
gi|126291169	2	71.90	5.45E-05	6.34	dihydropyrimidinase-related protein 3	1.08E+04	1706.05
gi|351698607	1	41.71	5.06E-04	6.37	CLIP-associating protein 1	155.71	991.34
gi|14388456	4	254.13	2.60E-04	6.39	hypothetical protein	3.12E+04	2.00E+05
gi|332235675	2 (1)	121.87	9.40E-03	6.40	transcription elongation factor B polypeptide 1-like	5.62E+04	8780.61
gi|548504378	2 (1)	105.15	1.00E-04	6.40	programmed cell death protein 5	5.22E+04	8158.83

gi|537153540	4 (2)	271.81	4.65E-03	6.41	bromodomain adjacent to zinc finger domain protein 1A	2.33E+05	3.63E+04
gi|687935	1	86.93	9.72E-03	6.47	PA700 subunit p48=ATP-dependent 20 S proteasome activator	9.25E+04	1.43E+04
gi|504137204	4 (2)	184.19	8.44E-05	6.49	talin-1	1.89E+04	2912.27
gi|46015221	3 (2)	100.70	4.93E-04	6.53	Chain A, Human SdhNADHINHIBITOR COMPLEX	1.54E+05	2.35E+04
gi|395857161	2 (1)	76.35	7.68E-03	6.54	zinc finger protein 185 isoform 5	2245.54	343.13
gi|512895554	1	44.97	2.06E-04	6.54	LOW QUALITY PROTEIN: angiomotin-like protein 1	1.72E+04	2637.04
gi|296481520	1	37.13	7.28E-03	6.60	TPA: inter-alpha globulin inhibitor H2 polypeptide	411.02	2714.67
gi|22538467	2	194.49	3.70E-04	6.61	proteasome subunit beta type-4	1.36E+04	8.97E+04
gi|448295	2	173.83	1.39E-03	6.63	TLS protein	7.32E+04	1.10E+04
gi|309271453	3 (2)	122.89	5.30E-04	6.71	splicing factor U2AF 65 kDa subunit-like	4.14E+04	2.78E+05
gi|13775198	1	76.10	3.96E-03	6.72	SH3 domain-binding glutamic acid-rich-like protein 3	2.40E+04	3574.90
gi|444728160	2 (1)	67.52	1.19E-03	6.72	40S ribosomal protein S3a	1.64E+04	2440.26
gi|511836643	1	36.23	6.67E-03	6.78	laminin subunit alpha-3 isoform X5	5.27E+04	7765.88
gi|507695278	2	84.86	1.76E-04	6.86	S-adenosylmethionine synthase isoform type-2-like	2.34E+04	1.60E+05
gi|291395669	8 (1)	488.57	5.29E-03	6.87	histone cluster 1, H2bc-like	2692.54	1.85E+04
gi|478519122	2 (1)	115.80	4.83E-04	7.04	ribosome-binding protein 1	1.10E+04	1560.69
gi|197099610	3	152.01	5.34E-03	7.11	C-1-tetrahydrofolate synthase, cytoplasmic	2836.67	2.02E+04
gi|397490603	8 (6)	457.32	3.78E-04	7.20	glucose-6-phosphate isomerase	4.38E+04	3.16E+05
gi|73696699	2 (1)	170.08	9.92E-05	7.28	ERp28 protein	3.38E+04	4648.44
gi|126723746	1	61.13	1.08E-04	7.29	serum albumin precursor	1.25E+04	9.14E+04
gi|441670702	3 (2)	206.42	1.20E-03	7.31	LOW QUALITY PROTEIN: peptidyl-prolyl cis-trans isomerase FKBP4	3459.49	2.53E+04

gi|5031755	2 (1)	98.34	8.03E-03	7.38	heterogeneous nuclear ribonucleoprotein R isoform 2	2.09E+04	2832.22
gi|5032069	1	69.48	7.32E-03	7.48	splicing factor 3B subunit 4	3067.98	2.29E+04
gi|39777597	2	187.48	2.54E-03	7.53	protein-glutamine gamma-glutamyltransferase 2 isoform a	1.32E+05	1.75E+04
gi|3095186	2	173.44	2.97E-04	7.57	cargo selection protein TIP47	2.14E+04	1.62E+05
gi|545521016	1	31.07	2.27E-03	7.73	maestro heat-like repeat family member 5 isoform X1	2582.01	2.00E+04
gi|55824584	1	80.84	1.18E-03	7.78	ribosomal protein S5	1.19E+05	1.52E+04
gi|530405491	4 (2)	229.28	7.04E-05	7.79	deoxyuridine 5'-triphosphate nucleotidohydrolase, mitochondrial isoform X1	1.09E+05	1.40E+04
gi|390462794	3	169.74	7.83E-05	7.82	60S ribosomal protein L15-like	1.99E+05	2.54E+04
gi|119621461	6	299.92	3.36E-03	7.90	ribosomal protein S7, isoform CRA_a	4.29E+05	5.42E+04
gi|4503477	1	60.45	9.30E-03	7.91	elongation factor 1-beta	3404.84	2.69E+04
gi|402905452	2 (1)	133.03	8.47E-06	7.96	delta(3,5)-Delta(2,4)-dienoyl-CoA isomerase, mitochondrial	5.97E+04	7503.63
gi|12847201	1	52.70	2.79E-03	8.03	unnamed protein product	3.00E+04	3728.94
gi|307109	1	81.94	6.88E-03	8.03	lysosomal membrane glycoprotein-1	3.96E+04	4939.53
gi|27806941	9	361.85	6.13E-05	8.16	alpha-1-antiproteinase precursor	3.55E+04	2.89E+05
gi|505841803	5	336.08	1.09E-04	8.23	heterogeneous nuclear ribonucleoprotein D0	4.64E+04	3.82E+05
gi|223036	2	203.53	1.27E-03	8.25	troponin C-like protein	2.10E+04	1.73E+05
gi|21730367	1	104.46	1.04E-03	8.36	Chain A, Ca2+-Binding Mimicry In The Crystal Structure Of The Eu3+-Bound Mutant Human Macrophage Capping Protein Cap G	1.94E+04	2320.23
gi|4757810	5 (4)	388.44	1.10E-03	8.43	ATP synthase subunit alpha, mitochondrial isoform a precursor	1.30E+04	1.10E+05
gi|431914265	2 (1)	125.64	9.78E-03	8.50	Paxillin	1.08E+05	1.27E+04

gi|507668095	3	206.63	2.80E-04	8.59	proteasome subunit alpha type-6	1.98E+05	2.31E+04
gi|494066	2	159.04	2.31E-03	8.64	Chain A, Three-Dimensional Structure Of Class Pi Glutathione S-Transferase From Human Placenta In Complex With S-Hexylglutathione At 2.8 Angstroms Resolution	1.18E+05	1.36E+04
gi|221041758	1	58.88	9.72E-03	8.72	unnamed protein product	364.50	3179.01
gi|77702086	20 (15)	1081.18	6.18E-07	8.74	heat shock protein 60	3.38E+04	2.96E+05
gi|3929737	1	32.40	1.85E-03	8.80	cathepsin L	2708.28	2.38E+04
gi|403263579	1	26.51	1.20E-03	9.07	LOW QUALITY PROTEIN: synaptotagmin-like protein 5	1.04E+04	1141.64
gi|537136955	29 (26)	1933.90	1.73E-05	9.10	actin, cytoplasmic 1	6.51E+05	5.92E+06
gi|472347285	2	60.58	1.36E-04	9.28	zinc finger homeobox protein 3-like isoform 1 [Odobenus rosmarus divergens]	2.87E+05	3.09E+04
gi|108967	1	55.54	6.98E-05	9.31	ubiquitin carboxyl-terminal proteinase - bovine (fragments)	2.14E+04	2300.10
gi|465987982	1	28.33	5.48E-04	9.32	protein FAM198B	1.13E+05	1.21E+04
gi|2529707	2	124.93	6.22E-04	9.41	Hpast	2.30E+04	2.17E+05
gi|26341628	4 (1)	261.64	1.77E-04	9.52	unnamed protein product	2.64E+04	2.51E+05
gi|505848858	2	78.27	9.26E-04	9.53	glutaredoxin-3	3172.67	3.02E+04
gi|162813	1	109.45	3.74E-04	9.59	cathepsin B, partial	1.40E+04	1.34E+05
gi|297272999	2	175.24	4.68E-03	9.67	hypothetical protein LOC706830 isoform 3	1.89E+04	1958.68
gi|537214960	5 (4)	256.26	9.33E-05	9.70	putative UV excision repair protein RAD23-like protein	1.13E+04	1.09E+05
gi|7022407	1	71.52	1.83E-03	9.77	unnamed protein product	667.72	6521.39
gi|262391	1	68.50	2.89E-04	9.86	Rig homolog [human, brain, Peptide Partial, 135 aa]	3031.67	2.99E+04
gi|296472298	5 (1)	171.67	1.53E-04	9.90	TPA: histone cluster 1, H2bd-like	8.49E+04	8580.09
gi|46249758	10 (1)	596.31	7.25E-03	9.94	Ezrin	1.17E+05	1.18E+04

gi|1263196	5	297.76	5.92E-04	9.95	AICAR formyltransferase/IMP cyclohydrolase bifunctional enzyme	1.45E+04	1.44E+05
gi|537227169	9 (1)	518.71	4.97E-06	10.13	glyceraldehyde-3-phosphate dehydrogenase	7.94E+04	7838.14
gi|209838088	1	81.30	8.51E-03	10.17	glutathione synthetase	2.91E+04	2865.55
gi|397466849	5 (2)	238.59	4.01E-03	10.21	keratin, type II cytoskeletal 8-like	3.96E+04	3876.87
gi|544412571	1	34.85	4.25E-03	10.37	actin-related protein T3 isoform X1	150.53	1561.60
gi|32363191	3 (1)	134.08	3.32E-03	10.50	Merlin	4.75E+04	4526.74
gi|4507401	1	53.40	3.08E-03	10.83	transcription factor A, mitochondrial isoform 1 precursor	227.31	2460.76
gi|90075304	3 (2)	189.30	2.87E-03	11.12	unnamed protein product	2.33E+05	2.10E+04
gi|221040626	3 (2)	259.24	3.18E-05	11.22	unnamed protein product	1.98E+04	2.22E+05
gi|436226	2	106.12	2.01E-03	11.29	KIAA0038	3699.23	4.18E+04
gi|5031753	2	109.13	3.37E-03	11.37	heterogeneous nuclear ribonucleoprotein H	3920.36	4.46E+04
gi|9507245	4 (1)	200.64	3.10E-03	11.45	14-3-3 protein gamma	503.83	5770.76
gi|344257037	2	109.11	9.49E-05	11.47	Purine nucleoside phosphorylase	2.12E+04	1846.96
gi|355748350	7 (1)	439.61	2.30E-04	11.48	Histone H2B.n	3511.57	4.03E+04
gi|16306978	14 (3)	790.45	6.32E-05	11.59	Annexin A2	1.83E+05	1.58E+04
gi|392354114	6 (2)	268.01	3.48E-03	11.59	LOW QUALITY PROTEIN: ezrin-like	4073.52	4.72E+04
gi|13928824	8 (4)	402.85	5.29E-04	11.86	14-3-3 epsilon, an isoform of 14-3-3 protein	4710.58	5.59E+04
gi|4506243	5 (4)	336.93	1.20E-04	11.88	polypyrimidine tract-binding protein 1 isoform a	4.45E+04	5.28E+05
gi|504172774	6 (3)	364.38	1.19E-04	11.99	elongation factor 2	1.44E+04	1.72E+05
gi|179832	2 (1)	117.89	1.52E-04	12.01	calnexin	1.14E+04	1.37E+05
gi|444724161	3 (1)	157.32	3.95E-04	12.03	FERM domain-containing protein 4A	1.33E+04	1102.29
gi|332218882	2 (1)	94.12	4.19E-03	12.12	LOW QUALITY PROTEIN: putative pre-mRNA-splicing factor ATP-dependent RNA helicase DHX15	4.01E+04	3309.55
gi|57143	3 (2)	147.78	2.56E-04	12.12	ribosomal protein S9	5.39E+04	4447.83

gi|114558626	4 (1)	198.75	4.10E-03	12.19	translationally-controlled tumor protein-like isoform 7	4.92E+04	4035.76
gi|5031635	6 (4)	355.61	6.00E-05	12.35	cofilin-1	1.98E+04	2.44E+05
gi|312005	1	55.46	1.37E-04	12.56	small nuclear ribonucleoprotein E	1.11E+05	8812.54
gi|77735779	3	190.34	8.62E-05	12.61	inosine-5'-monophosphate dehydrogenase 2	3.18E+04	2519.92
gi|3097244	1	72.58	1.12E-03	12.66	ribosomal protein S14	3.18E+04	2514.93
gi|219519732	4 (1)	188.06	2.31E-04	12.69	Psma8 protein	1.07E+05	8466.26
gi|291387421	5 (3)	243.35	3.50E-03	12.84	heat shock 70kDa protein 9	3616.88	4.64E+04
gi|4502013	5 (1)	230.10	4.18E-04	12.85	adenylate kinase 2, mitochondrial isoform a	2.20E+04	1710.71
gi|238684652	1	57.26	3.39E-03	13.00	MHC class I antigen	2.08E+04	1601.40
gi|109470171	1	75.79	1.38E-04	13.02	DNA polymerase epsilon subunit 4-like	8.10E+04	6224.77
gi|533118782	2 (1)	64.28	3.87E-03	13.03	protein ELYS isoform X1	3.05E+04	2339.29
gi|532081396	2 (1)	65.82	2.51E-03	13.12	collagen alpha-3(VI) chain	7604.79	579.79
gi|62087310	2	130.17	3.13E-03	13.13	gamma filamin variant	4.48E+04	3412.00
gi|7305107	1	47.01	1.36E-04	13.26	protein C10	8531.84	643.62
gi|65987	12 (1)	646.49	5.24E-03	13.40	glyceraldehyde-3-phosphate dehydrogenase (phosphorylating) (EC 1.2.1.12) - pig	794.32	59.29
gi|529281076	2 (1)	122.59	2.03E-03	13.42	Chain A, Crystal Structure Of Rat Galectin-1 In Complex With Lactose	9.58E+04	7137.93
gi|187302	3 (1)	134.89	2.94E-03	13.64	epithelial cell marker protein 1	6.06E+04	4446.22
gi|297412	1	38.30	2.54E-03	13.86	thrombin inhibitor	1.02E+04	737.82
gi|533125814	7 (1)	350.85	6.59E-03	13.87	radixin isoform X3	2651.07	3.68E+04
gi|478493086	2	105.52	5.52E-04	14.00	LOW QUALITY PROTEIN: ribosomal protein L5	6.93E+04	4945.74
gi|410968032	2 (1)	100.57	2.33E-03	14.22	ATP synthase subunit b, mitochondrial	2502.54	3.56E+04
gi|74267962	5 (1)	280.03	3.66E-03	14.23	ALB protein	6367.64	9.06E+04
gi|189617	1	121.63	3.84E-03	14.43	protein PP4-X	1.38E+04	955.20
gi|9802306	1	144.93	3.54E-05	14.43	DNA-binding protein TAXREB107	1.54E+05	1.07E+04

gi|345305212	1	37.92	7.59E-04	14.45	coiled-coil domain-containing protein 28A-like	6185.29	428.19
gi|4502303	2	146.03	5.05E-05	14.49	ATP synthase subunit O, mitochondrial precursor	2885.96	4.18E+04
gi|351701782	1	75.84	2.81E-03	14.66	Protein S100-A10	6095.27	8.93E+04
gi|7710086	1	72.80	4.47E-03	14.91	ras-related protein Rab-10	1.73E+04	1162.81
gi|291385575	2 (1)	85.45	1.48E-03	14.97	leucine aminopeptidase 3	6557.53	437.97
gi|10120888	3 (1)	132.85	6.09E-03	14.98	Chain A, The Crystal Structure Of Human Barrier-To-Autointegration Factor (Baf)	1.49E+05	9969.20
gi|4506699	1	54.65	2.06E-03	15.07	40S ribosomal protein S21	853.45	1.29E+04
gi|179531	2	70.28	2.96E-04	15.08	IgE-binding protein	4.24E+04	2810.33
gi|6014493	2 (1)	143.72	6.52E-04	15.20	PLIC-2	5.13E+04	3372.13
gi|392344177	1	33.45	7.38E-04	15.23	zinc finger protein 850-like	2177.53	3.32E+04
gi|197304975	2 (1)	84.05	6.51E-03	15.41	Chain A, Oxidized And Reduced Forms Of Human Peroxiredoxin 5	1491.69	2.30E+04
gi|56606098	1	36.63	8.14E-03	15.55	complement C1q subcomponent subunit C precursor	1042.49	1.62E+04
gi|432094225	2 (1)	124.23	8.35E-04	15.65	Hydroxymethylglutaryl-CoA synthase, cytoplasmic	1.34E+04	854.22
gi|90080203	2 (1)	109.85	6.66E-03	15.68	unnamed protein product	5764.77	367.55
gi|4757900	3	87.06	1.05E-05	15.98	calreticulin precursor	3513.81	5.61E+04
gi|55727871	2 (1)	90.61	2.60E-03	16.67	hypothetical protein	7.43E+04	4456.51
gi|19527026	1	65.04	3.99E-03	16.74	leucine-rich repeat-containing protein 59	3.07E+04	1831.93
gi|297302457	11 (1)	604.42	7.69E-04	17.00	heat shock 70 kDa protein 1A/1B, partial	1127.08	1.92E+04
gi|28277147	1	64.54	1.99E-03	17.15	Metadherin	550.92	9446.10
gi|53035	1	71.16	3.83E-03	17.17	cyclophilin CyP-S1	2077.59	3.57E+04
gi|390462052	5 (1)	222.63	3.79E-03	17.28	tropomyosin alpha-3 chain-like	944.31	1.63E+04
gi|511864079	15 (2)	816.61	7.94E-03	17.81	tubulin beta-3 chain	4.82E+04	2703.44
gi|4506597	6 (5)	396.94	5.87E-05	18.01	60S ribosomal protein L12	5341.60	9.62E+04

gi|12963573	1	62.62	5.02E-03	18.02	dCTP pyrophosphatase 1	1086.42	1.96E+04
gi|355693385	5 (1)	243.25	3.39E-03	18.26	hypothetical protein EGK_18319, partial	4.10E+04	2242.22
gi|537141603	2	97.46	7.32E-04	18.46	sequestosome-1	3275.55	6.05E+04
gi|291384816	2 (1)	74.26	8.21E-03	18.68	catalase	58.08	1085.06
gi|528765647	2 (1)	90.91	2.43E-03	19.01	peptidyl-prolyl cis-trans isomerase FKBP4	2.45E+04	1287.67
gi|537271418	3 (1)	120.19	4.99E-04	19.03	chromodomain-helicase-DNA-binding protein 1	841.48	1.60E+04
gi|28204689	1	56.89	2.42E-04	19.22	ribosomal protein S4	888.28	1.71E+04
gi|344240817	1	54.82	5.23E-04	19.39	Dynein heavy chain 5, axonemal	2.73E+04	5.30E+05
gi|351713549	2	100.76	1.30E-04	19.54	Prohibitin	5336.58	1.04E+05
gi|466023766	4 (1)	211.79	2.70E-03	19.57	adenosylhomocysteinase isoform 1	397.17	7773.94
gi|28077049	1	96.15	3.15E-05	19.71	charged multivesicular body protein 4b	8.41E+04	4265.88
gi|537129831	6 (1)	346.66	9.91E-04	20.21	tubulin alpha-1B chain	5738.69	1.16E+05
gi|488563063	1	44.16	8.87E-04	20.29	glycine N-acyltransferase-like protein 3	5.42E+04	1.10E+06
gi|47847498	4 (1)	194.46	4.52E-04	20.78	mFLJ00279 protein	5016.16	241.44
gi|187609338	2 (1)	101.92	4.00E-04	20.99	Chain A, Crystal Structure Of The Extracellular Portion Of Hab18gCD147	1668.44	3.50E+04
gi|32425649	1	46.16	2.78E-04	21.16	EIF4B protein, partial	2.18E+04	1032.40
gi|4502565	3 (2)	145.79	1.41E-04	21.39	calpain small subunit 1	1305.44	2.79E+04
gi|511910713	4 (2)	239.30	8.11E-06	21.47	keratin, type II cytoskeletal 7	7.91E+04	3683.77
gi|162678	2	135.99	1.11E-04	22.13	apolipoprotein A-I precursor	2780.21	6.15E+04
gi|441592694	3 (1)	139.86	1.45E-04	22.49	actin, cytoplasmic type 5-like isoform 1	363.15	8166.97
gi|114665952	6 (4)	288.19	2.61E-04	22.53	complement component 1 Q subcomponent-binding protein, mitochondrial isoform 6	5566.94	1.25E+05
gi|441669563	6 (3)	464.53	8.70E-04	22.62	nucleolin	1.21E+04	2.74E+05
gi|358419864	1	39.08	8.85E-03	22.78	mucin-19	8.75E+05	3.84E+04
gi|301770723	6 (1)	303.08	1.43E-03	22.98	LOW QUALITY PROTEIN: elongation	911.00	2.09E+04

					factor 1-alpha, oocyte form-like		
gi|505779444	2 (1)	79.47	6.64E-04	23.09	fetuin-B-like	1628.07	3.76E+04
gi|544480052	1	39.32	8.52E-03	23.86	zinc finger protein 415-like	381.89	9112.99
gi|6755728	1	85.53	3.86E-04	24.03	transcription elongation factor A protein 1 isoform 2	2760.00	6.63E+04
gi|109107090	2	160.26	9.63E-05	24.10	protein S100-A11	8548.22	2.06E+05
gi|444725708	8 (1)	394.42	2.14E-04	24.78	60S ribosomal protein L7a	1599.44	3.96E+04
gi|471382122	1	27.73	9.72E-03	24.94	retinal-specific ATP-binding cassette transporter	3349.60	8.35E+04
gi|297289595	3 (2)	131.18	1.24E-05	25.25	protein disulfide-isomerase A4-like	2571.61	6.49E+04
gi|5453541	1	152.59	3.73E-04	25.36	anterior gradient protein 2 homolog precursor	3377.01	8.56E+04
gi|545834628	2 (1)	80.36	1.01E-03	25.82	LOW QUALITY PROTEIN: YTH domain family protein 2	2579.13	6.66E+04
gi|189308	1	45.69	2.46E-03	26.44	nucleobindin	6440.61	243.59
gi|344238877	2 (1)	89.05	1.79E-03	26.60	Transcription factor BTF3	2039.96	5.43E+04
gi|296483028	1	37.93	9.56E-03	27.06	TPA: hCG1994130-like	5.07E+04	1874.81
gi|200812	2	80.07	5.48E-05	27.20	LLRep3 protein	4.39E+04	1615.14
gi|524948880	2 (1)	83.02	5.25E-04	27.97	UDP-glucose 6-dehydrogenase	2180.41	6.10E+04
gi|306875	8 (5)	527.44	7.71E-06	28.82	C protein	2.98E+04	8.59E+05
gi|545178330	2 (1)	71.23	2.85E-03	28.86	LOW QUALITY PROTEIN: Na(+)/H(+) exchange regulatory cofactor NHE-RF1	252.32	7280.97
gi|5031595	2 (1)	101.89	3.08E-04	29.64	actin-related protein 2/3 complex subunit 4 isoform a	1.51E+04	509.00
gi|507653930	2 (1)	137.16	1.42E-03	29.78	translation initiation factor IF-2-like	9.66E+04	3242.71
gi|348554675	9 (1)	471.96	1.15E-03	30.74	creatine kinase B-type isoform X1	3.00E+04	976.49
gi|27806751	5 (4)	228.06	1.30E-05	31.03	alpha-2-HS-glycoprotein precursor	3.78E+04	1.17E+06
gi|332230006	11 (8)	664.66	9.31E-05	31.26	78 kDa glucose-regulated protein isoform 1	2.15E+04	6.73E+05
gi|1022961	3 (1)	143.94	9.15E-04	32.18	HuR RNA binding protein	1.44E+04	447.83

gi|73760405	1	68.22	2.30E-03	32.45	thymopoietin isoform beta	694.71	2.25E+04
gi|62460494	2 (1)	228.13	1.70E-03	33.20	hemoglobin fetal subunit beta	4.71E+04	1419.77
gi|478529796	3 (1)	69.15	9.12E-03	34.35	kinesin-like protein KIF21B	395.27	1.36E+04
gi|119581823	2	68.47	3.43E-04	34.74	hCG1979282	7.55E+04	2173.41
gi|126305744	2 (1)	72.87	8.34E-03	35.07	UMP-CMP kinase-like	4.56E+04	1300.60
gi|10140853	1	136.15	1.27E-03	35.74	acyl-CoA-binding protein isoform 1	3437.35	1.23E+05
gi|426232407	2 (1)	80.25	7.25E-04	37.62	uncharacterized protein LOC101107705	1.50E+04	399.08
gi|332250529	2 (1)	61.52	1.61E-03	37.76	uncharacterized protein C5orf42 homolog	1127.40	4.26E+04
gi|4092058	4 (1)	198.06	1.73E-04	37.83	proteasome subunit HSPC	1.45E+04	382.34
gi|1351907	6 (1)	334.89	9.51E-05	37.91	Serum albumin	3543.02	1.34E+05
gi|109122464	4 (1)	186.83	1.17E-03	38.75	l-lactate dehydrogenase B chain	766.37	2.97E+04
gi|55732000	21 (5)	1488.99	1.65E-04	40.13	hypothetical protein	1.33E+04	5.32E+05
gi|62897945	19 (1)	1820.45	4.31E-04	40.21	enolase 1 variant	2.37E+05	5897.21
gi|4503481	3	174.01	5.82E-05	40.30	elongation factor 1-gamma	1.50E+05	3713.90
gi|12082136	19 (5)	1073.58	6.09E-05	41.42	heat shock protein 90 alpha	5125.13	2.12E+05
gi|444721795	6 (1)	228.92	1.12E-05	42.25	ATP-binding cassette sub-family A member 3	9896.88	234.24
gi|511846280	5 (4)	243.59	1.03E-05	43.58	transitional endoplasmic reticulum ATPase isoform X1	4830.45	2.10E+05
gi|77736017	1	59.51	1.42E-03	44.09	NAD(P)H dehydrogenase [quinone] 1	2.19E+04	495.66
gi|33239320	1	50.94	9.99E-04	44.58	olfactory receptor 96	1.64E+04	368.39
gi|533137684	2	113.43	6.04E-06	47.00	60S ribosomal protein L14	1.80E+05	3822.98
gi|148681385	3 (2)	156.07	3.59E-05	47.16	succinate dehydrogenase complex, subunit B, iron sulfur (Ip), isoform CRA_b	845.34	3.99E+04
gi|9955206	1	50.21	4.17E-04	48.17	Chain B, Crystal Structure Of A Rac-Rhogdi Complex	1586.98	7.64E+04
gi|14249348	1	48.53	1.40E-03	53.31	thioredoxin domain-containing protein 17	3.67E+04	688.74
gi|4506743	1	80.03	4.18E-04	53.51	40S ribosomal protein S8	468.66	2.51E+04

gi|544430751	20 (7)	1259.04	4.57E-04	53.57	uncharacterized protein LOC102118097	2.47E+04	1.32E+06
gi|472391178	3 (1)	140.25	8.74E-04	54.62	T-complex protein 1 subunit epsilon isoform 2	557.49	3.04E+04
gi|403294184	2 (1)	105.84	7.31E-04	67.46	T-complex protein 1 subunit gamma	2.91E+04	430.91
gi|4759154	1	50.63	4.64E-05	71.57	synaptosomal-associated protein 29	80.36	5751.58
gi|431904909	4 (1)	186.64	3.12E-03	71.61	Translationally-controlled tumor protein	1.42E+04	198.75
gi|3088342	1	80.07	2.66E-03	73.46	ribosomal protein S23	3.30E+04	449.01
gi|201723	1	63.99	2.32E-03	75.19	t complex polypeptide 1, partial	2.10E+04	279.45
gi|3212116	2 (1)	152.17	5.88E-05	80.86	prefoldin subunit 2	8596.85	106.32
gi|344298464	4 (1)	215.87	7.64E-05	83.45	hypoxanthine-guanine phosphoribosyltransferase-like	1.89E+04	226.41
gi|253706	6 (4)	382.85	1.54E-05	83.55	14-3-3 protein zeta chain [cattle, brain, Peptide, 245 aa]	4629.70	3.87E+05
gi|3088338	1	63.41	7.49E-03	83.69	ribosomal protein S10	5.77E+04	689.98
gi|156766050	2 (1)	88.41	5.77E-04	90.16	protein AHNAK2	1.52E+04	168.12
gi|512876729	2 (1)	65.17	1.72E-04	93.39	LOW QUALITY PROTEIN: nicotinamide phosphoribosyltransferase	166.68	1.56E+04
gi|17933772	1	78.03	2.26E-03	93.78	protein S100-A16	4.83E+04	514.57
gi|524968854	2 (1)	99.52	1.93E-04	94.84	acidic leucine-rich nuclear phosphoprotein 32 family member E isoform X1	55.87	5298.19
gi|488538974	1	39.76	8.30E-04	108.15	probable asparagine--tRNA ligase, mitochondrial isoform 1	4213.84	4.56E+05
gi|7739445	2	140.29	3.22E-04	111.11	hnRNP 2H9B	135.42	1.50E+04
gi|444721551	2 (1)	82.47	1.76E-03	123.39	60S ribosomal protein L5	1.32E+05	1068.81
gi|348567745	2	74.04	6.81E-04	127.91	T-complex protein 1 subunit zeta-2 isoformX1	126.79	1.62E+04
gi|488566468	3 (2)	135.48	4.34E-06	139.86	far upstream element-binding protein 1 isoform 1	2.07E+04	148.04
gi|126343505	2 (1)	72.05	9.48E-03	169.45	structural maintenance of chromosomes protein 6-like	5.84	989.57

gi|251370	1	70.85	4.39E-03	210.68	acid phosphatase isoenzyme Af [human, erythrocytes, Peptide, 157 aa]	6276.10	29.79
gi|533203458	1	40.28	9.02E-04	276.86	LOW QUALITY PROTEIN: deoxyribonuclease-1-like 1	71.77	1.99E+04
gi|109465383	2 (1)	125.48	8.07E-03	282.97	60S acidic ribosomal protein P0-like	4955.03	17.51
gi|28461243	1	36.61	6.54E-03	305.22	histidine triad nucleotide-binding protein 1	12.04	3676.35
gi|512886886	4 (1)	224.23	1.31E-04	313.23	filamin-B isoform X1	217.33	6.81E+04
gi|160964410	3 (1)	123.03	8.32E-03	325.56	transferrin receptor 1	23.29	7583.21
gi|5453559	2 (1)	173.48	6.84E-05	367.48	ATP synthase subunit d, mitochondrial isoform a	375.90	1.38E+05
gi|11034825	1	101.63	6.31E-03	456.18	methionine adenosyltransferase 2 subunit beta isoform 1	1.22E+04	26.80
gi|544402295	2 (1)	120.19	6.31E-03	468.90	interleukin enhancer-binding factor 2 isoform X1	7669.05	16.36
gi|4502297	1	70.57	7.14E-03	549.36	ATP synthase subunit delta, mitochondrial precursor	23.56	1.29E+04
gi|42543459	2 (1)	57.10	1.95E-03	584.20	Chain A, Crystal Structure Of A Mu-Like Calpain	1918.43	3.28
gi|332820815	2 (1)	102.03	7.90E-03	596.45	succinate dehydrogenase [ubiquinone] flavoprotein subunit, mitochondrial-like isoform 1	36.97	2.21E+04
gi|402893095	3 (2)	137.43	7.98E-03	640.18	neutral alpha-glucosidase AB isoform 1	40.75	2.61E+04
gi|466045811	2 (1)	98.24	2.44E-03	739.07	lon protease homolog, mitochondrial	3766.90	5.10
gi|193787759	1	77.87	2.72E-04	1025.38	unnamed protein product	0.28	285.09
gi|6225527	1	44.24	1.26E-03	1398.48	Isopentenyl-diphosphate Delta-isomerase 1	4656.16	3.33
gi|126340084	3 (1)	126.32	2.22E-03	1560.34	prohibitin-2-like	7.17	1.12E+04
gi|26336162	1	45.86	5.39E-03	1730.64	unnamed protein product	27.31	4.73E+04
gi|1552242	2 (1)	127.73	1.75E-05	1967.83	hRlf beta subunit (p102 protein)	5499.13	2.79
gi|4558043	1	138.13	5.25E-03	2379.95	Chain B, Structure Of Nedd8	1.47E+05	61.96
gi|444517749	2 (1)	101.37	1.38E-03	2694.30	Importin subunit beta-1	1.33E+04	4.93

gi|7709912	1	53.33	5.12E-05	3154.06	phosphoglucomutase 1	174.10	0.06
gi|4504253	6 (1)	423.58	1.70E-03	3277.41	histone H2AX	6.06	1.99E+04
gi|507541388	1	35.75	8.93E-04	4024.93	coiled-coil domain-containing protein 102B	6.98	2.81E+04
gi|344256722	1	71.08	1.26E-03	6530.73	High mobility group protein 1-like 10	4.74	3.09E+04
gi|9506761	1	78.29	1.78E-08	3.57E+06	glucosamine 6-phosphate N-acetyltransferase	1.67E+04	4.67E-03
gi|1706754	1	49.06	2.26E-06	Infinity	Fatty acid-binding protein, epidermal	238.41	0.00
gi|194389534	1	28.19	1.01E-04	Infinity	unnamed protein product	0.00	1557.80
gi|296215020	2 (1)	73.50	6.44E-06	Infinity	small nuclear ribonucleoprotein-associated protein N-like	1.20E+04	0.00
gi|297296973	2 (1)	122.89	2.26E-08	Infinity	reticulocalbin-2	0.00	2.76E+04
gi|35959	14 (2)	899.56	7.20E-07	Infinity	tubulin 5-beta	0.00	2.48E+04
gi|432092846	2 (1)	123.83	1.28E-07	Infinity	Paxillin	3633.80	0.00
gi|440909513	2 (1)	127.95	1.37E-03	Infinity	Paxillin, partial	0.00	271.78
gi|444721584	2 (1)	65.49	2.03E-04	Infinity	Heterogeneous nuclear ribonucleoprotein D-like protein	0.00	401.06
gi|472389073	1	41.39	2.75E-05	Infinity	retinoic acid receptor responder protein 3	0.00	4398.76
gi|4758762	1	44.16	3.84E-07	Infinity	asparagine--tRNA ligase, cytoplasmic	4453.44	0.00
gi|505785940	1	51.76	1.15E-07	Infinity	protein shisa-2 homolog	8974.40	0.00
gi|507635704	3 (1)	108.57	3.02E-06	Infinity	60S ribosomal protein L4, partial	0.00	1.69E+04
gi|532057143	2 (1)	69.65	4.24E-05	Infinity	nascent polypeptide-associated complex subunit alpha, muscle-specific form	867.76	0.00

* significant expression : $p<0.05$.

Table S3. Identified differentially expressed proteins between LNCaP and LNCaPRR cell lines

Accession	Peptides	Score	Anova (p)*	Fold	Description	Average Normalised Abundances	
						**LNCaP**	**LNCaPRR**
gi|4758638	5	292.46	1.99E-03	1.55	peroxiredoxin-6	8.26E+04	1.28E+05
gi|296234804	4 (2)	249.97	0.02	1.55	transcription intermediary factor 1-beta isoform 2	5.42E+04	3.50E+04
gi|38013966	13 (12)	700.12	0.02	1.58	TKT protein	1.59E+05	2.51E+05
gi|530405491	4 (2)	229.28	3.28E-04	1.62	deoxyuridine 5'-triphosphate nucleotidohydrolase, mitochondrial isoform X1	2.18E+04	3.52E+04
gi|340219	2	111.15	0.01	1.77	vimentin	1.40E+04	24710
gi|507574478	5 (4)	193.10	0.03	1.81	ran-specific GTPase-activating protein	7.13E+04	3.95E+04
gi|27806751	5 (4)	228.06	9.20E-04	1.82	alpha-2-HS-glycoprotein precursor	5.42E+05	9.85E+05
gi|13928824	8 (4)	402.85	7.17E-04	1.83	14-3-3 protein,epsilon	4.95E+04	90341.3
gi|528755289	5 (3)	264.91	0.03	1.93	translational activator GCN1	1.40E+04	2.70E+04
gi|28614	9 (8)	457.77	8.39E-03	2.04	aldolase A, fructose-bisphosphate	1.20E+05	2.45E+05
gi|472347285	2	60.58	0.05	2.26	zinc finger homeobox protein 3-like isoform 1	7.68E+04	1.73E+05
gi|309271453	3 (2)	122.89	5.14E-03	2.37	splicing factor U2AF 65 kDa subunit-like	1.25E+05	2.96E+05
gi|2501351	4	194.48	0.04	2.58	Serotransferrin	4.99E+04	1.29E+05
gi|74222288	4 (3)	209.31	4.94E-03	2.87	unnamed protein product	2.62E+04	7.52E+04
gi|3095186	2	173.44	0.01	2.93	cargo selection protein TIP47	2.03E+04	5.96E+04
gi|426241785	5 (3)	171.85	0.02	3.00	ubiquitin-60S ribosomal protein L40-like	1.50E+04	4.49E+04
gi|81294349	1	87.35	0.05	3.05	DHX9 protein	4644.93	1525.13
gi|332218133	2	144.26	0.01	3.06	prolyl endopeptidase	2.27E+04	6.93E+04
gi|55733515	1	78.72	0.02	3.09	hypothetical protein	8026.79	2.48E+04
gi|109113948	3	209.04	0.02	3.12	40S ribosomal protein S17-like isoform 2	8.96E+04	2.80E+05
gi|4827038	2	84.08	2.64E-03	3.13	tumor protein D52 isoform 3	8.21E+04	2.63E+04
gi|35218	2 (1)	127.76	0.03	3.14	unnamed protein product	2940.27	9244.72

gi|225029822	1	51.35	0.02	3.16	C-C chemokine receptor 2 transcript variant 2	1.42E+04	4.49E+04
gi|3097244	1	72.58	2.33E-03	3.25	ribosomal protein S14	4.63E+04	1.42E+04
gi|5901922	2	78.85	0.02	3.28	hsp90 co-chaperone Cdc37	4.96E+04	1.51E+04
gi|12847201	1	52.70	1.66E-03	3.32	unnamed protein product	1.01E+04	3.37E+04
gi|432096880	6 (5)	340.59	8.67E-04	3.35	Methylcrotonoyl-CoA carboxylase subunit alpha, mitochondrial	1.62E+05	5.44E+05
gi|444724161	3 (1)	157.32	7.03E-03	3.39	FERM domain-containing protein 4A	2757.07	9342.51
gi|5031755	2 (1)	98.34	0.05	3.42	heterogeneous nuclear ribonucleoprotein R isoform 2	5023.36	1.72E+04
gi|507695278	2	84.86	3.71E-03	3.42	S-adenosylmethionine synthase isoform type-2-like	1.13E+05	3.29E+04
gi|181575	1	88.40	0.05	3.46	dihydrolipoamide dehydrogenase precursor	2.50E+04	8.67E+04
gi|14388456	4	254.13	1.83E-03	3.48	hypothetical protein	1.07E+05	3.06E+04
gi|512905160	2	179.78	4.45E-04	3.50	peptidyl-prolyl cis-trans isomerase FKBP3 isoform X1	1.39E+04	4.85E+04
gi|57143	3 (2)	147.78	0.03	3.50	ribosomal protein S9	7645.80	2.67E+04
gi|200812	2	80.07	0.01	3.55	LLRep3 protein	5868.23	2.08E+04
gi|426232708	2 (1)	98.19	4.57E-03	3.61	proteasome activator complex subunit 1	4176.77	1.51E+04
gi|432094225	2 (1)	124.23	1.21E-03	3.65	Hydroxymethylglutaryl-CoA synthase, cytoplasmic	3402.49	1.24E+04
gi|512895554	1	44.97	0.03	3.75	LOW QUALITY PROTEIN: angiomotin-like protein 1	2.09E+04	5568.32
gi|545834628	2 (1)	80.36	0.04	3.86	LOW QUALITY PROTEIN: YTH domain family protein 2	4.28E+04	1.11E+04
gi|537179976	1	39.14	1.01E-03	3.95	exophilin-5	1.26E+05	5.00E+05
gi|344257037	2	109.11	5.19E-03	3.96	Purine nucleoside phosphorylase	1931.93	7648.67
gi|397490603	8 (6)	457.32	5.97E-03	4.03	glucose-6-phosphate isomerase	1.90E+05	4.71E+04
gi|403290048	2 (1)	119.41	8.33E-03	4.04	drebrin	3.05E+04	7545.77
gi|189308	1	45.69	0.05	4.04	nucleobindin	1159.43	4682.25
gi|537136955	29 (26)	1933.90	3.42E-03	4.07	actin, cytoplasmic 1	2.44E+06	6.01E+05
gi|344249729	2 (1)	125.37	0.02	4.10	Phosphoglycerate kinase 2	8.32E+04	2.03E+04

gi|20987362	1	121.82	0.01	4.12	TXLNA protein, partial	1.81E+04	4380.48
gi|56554533	1	61.88	0.02	4.14	Chain J, Clathrin D6 Coat	4282.66	1.77E+04
gi|444728160	2 (1)	67.52	0.03	4.22	40S ribosomal protein S3a	1731.66	7309.51
gi|5729953	5	232.76	0.04	4.29	nuclear migration protein nudC	9.41E+04	2.19E+04
gi|4755083	1	96.93	1.81E-03	4.29	density regulated protein drp1	5.28E+04	1.23E+04
gi|703093	3	142.12	7.04E-03	4.32	serine hydroxymethyltransferase, partial	7.65E+04	1.77E+04
gi|397465809	6	316.50	0.02	4.35	macrophage migration inhibitory factor	3.12E+05	1.36E+06
gi|19923193	4 (1)	175.51	0.04	4.49	hsc70-interacting protein isoform 1	1.03E+04	2305.07
gi|332218882	2 (1)	94.12	0.04	4.50	LOW QUALITY PROTEIN: putative pre-mRNA-splicing factor ATP-dependent RNA helicase DHX15	4721.62	2.12E+04
gi|344287751	9 (4)	427.11	3.59E-04	4.55	peroxiredoxin-1-like	1.97E+05	4.32E+04
gi|296481520	1	37.13	5.86E-03	4.57	TPA: inter-alpha globulin inhibitor H2 polypeptide	1324.97	6053.51
gi|431905273	5 (1)	339.66	0.02	4.68	Endoplasmin	3012.94	1.41E+04
gi|1535	4 (3)	149.01	0.03	4.74	unnamed protein product	3952.12	1.87E+04
gi|397464729	9 (7)	585.75	4.02E-04	4.79	peptidyl-prolyl cis-trans isomerase A-like, partial	1.27E+06	2.64E+05
gi|14141152	7 (5)	434.01	3.54E-04	4.84	heterogeneous nuclear ribonucleoprotein M isoform a	1.11E+05	2.28E+04
gi|533183091	2 (1)	101.27	0.02	4.87	RNA-binding motif protein, X chromosome isoform X4	1.31E+04	6.37E+04
gi|291385575	2 (1)	85.45	7.67E-03	4.91	leucine aminopeptidase 3	1456.13	7146.19
gi|5031857	8 (3)	379.94	0.01	4.96	L-lactate dehydrogenase A chain isoform 1	2.72E+04	1.35E+05
gi|514475709	1	35.29	9.32E-03	5.11	vomeronasal type-2 receptor 26-like	690.16	3523.91
gi|504137204	4 (2)	184.19	6.09E-03	5.14	talin-1	4669.22	2.40E+04
gi|225719953	2 (1)	112.23	1.53E-03	5.14	selenium binding protein 1 (predicted)	2.68E+04	5214.83
gi|544412571	1	34.85	0.02	5.14	actin-related protein T3 isoform X1	665.40	3421.93
gi|444721520	9	804.93	7.56E-04	5.21	Nucleoside diphosphate kinase B	1.71E+06	3.28E+05
gi|355693385	5 (1)	243.25	0.01	5.25	hypothetical protein EGK_18319, partial	7081.50	3.71E+04

gi|3212116	2 (1)	152.17	0.05	5.30	prefoldin subunit 2	2253.92	1.20E+04
gi|512876729	2 (1)	65.17	5.71E-03	5.31	LOW QUALITY PROTEIN: nicotinamide phosphoribosyltransferase	4782.57	901.02
gi|466049892	2 (1)	98.46	2.24E-03	5.32	cathepsin D	1.10E+04	5.84E+04
gi|344248192	2 (1)	127.85	1.24E-03	5.33	Signal recognition particle 14 kDa protein	1.18E+04	6.28E+04
gi|537218817	3 (1)	114.17	4.51E-03	5.37	myomesin-3	7041.92	3.78E+04
gi|5453541	1	152.59	2.48E-04	5.40	anterior gradient protein 2 homolog precursor	5.72E+04	1.06E+04
gi|5032069	1	69.48	6.47E-03	5.40	splicing factor 3B subunit 4	4.71E+04	8732.90
gi|524930080	6 (1)	347.43	5.08E-03	5.50	polyadenylate-binding protein 1-like	1.47E+04	8.08E+04
gi|119602640	4	248.84	5.35E-04	5.51	eukaryotic translation elongation factor 1 delta (guanine nucleotide exchange protein), isoform CRA_e	1.42E+05	2.57E+04
gi|448295	2	173.83	4.13E-04	5.55	TLS protein	1.77E+04	9.81E+04
gi|403294184	2 (1)	105.84	3.01E-03	5.56	T-complex protein 1 subunit gamma	1268.66	7058.29
gi|444727966	2 (1)	60.19	0.03	5.65	Alpha-aminoadipic semialdehyde dehydrogenase	1287.86	7282.07
gi|511836643	1	36.23	0.04	5.65	laminin subunit alpha-3 isoform X5	8618.94	4.87E+04
gi|505841803	5	336.08	2.42E-03	5.73	heterogeneous nuclear ribonucleoprotein D0	3.13E+05	5.46E+04
gi|395530406	2	71.30	2.76E-04	5.73	uncharacterized protein LOC100927683	9324.76	5.34E+04
gi|3929737	1	32.40	8.98E-03	5.83	cathepsin L	4137.77	709.82
gi|687935	1	86.93	0.02	5.85	PA700 subunit p48=ATP-dependent 20 S proteasome activator	9602.79	5.62E+04
gi|74007267	2	80.10	9.51E-03	5.90	poly(rC)-binding protein 1-like isoform X2	1.62E+04	9.57E+04
gi|297302457	11 (1)	604.42	0.03	6.01	heat shock 70 kDa protein 1A/1B, partial	1.98E+04	3299.45
gi|537191106	4 (1)	140.09	6.50E-03	6.04	nuclear factor related to kappa-B-binding protein	2.19E+04	3623.36
gi|119621461	6	299.92	2.10E-03	6.09	ribosomal protein S7, isoform CRA_a	3.98E+04	2.42E+05
gi|4826760	3	189.31	9.60E-05	6.09	heterogeneous nuclear ribonucleoprotein F	1.08E+05	1.77E+04
gi|17028367	1	38.77	0.01	6.17	Similar to gelsolin (amyloidosis, Finnish type), partial	848.48	5239.29

gi|26341628	4 (1)	261.64	9.86E-05	6.25	unnamed protein product	4.78E+05	7.65E+04
gi|528765647	2 (1)	90.91	3.78E-03	6.40	peptidyl-prolyl cis-trans isomerase FKBP4	1326.48	8490.96
gi|505848858	2	78.27	9.87E-04	6.41	glutaredoxin-3	3.84E+04	5989.10
gi|189617	1	121.63	0.01	6.43	protein PP4-X	2004.24	1.29E+04
gi|296209622	3	103.70	2.85E-04	6.53	replication protein A 14 kDa subunit-like	5.81E+04	8895.18
gi|537214960	5 (4)	256.26	9.93E-03	6.54	putative UV excision repair protein RAD23-like protein	7.95E+04	1.22E+04
gi|5031753	2	109.13	1.08E-03	6.62	heterogeneous nuclear ribonucleoprotein H	6.52E+04	9845.60
gi|531997376	1	95.08	0.03	6.62	proteasome subunit alpha type-3 isoform X1	1934.20	1.28E+04
gi|432109189	3 (1)	99.64	9.44E-04	6.72	Isocitrate dehydrogenase [NADP] cytoplasmic	4.11E+04	6118.25
gi|4759154	1	50.63	8.89E-03	6.91	synaptosomal-associated protein 29	856.59	123.94
gi|109470171	1	75.79	6.45E-04	7.21	DNA polymerase epsilon subunit 4-like	1.22E+04	8.83E+04
gi|507984758	1	43.47	0.01	7.22	caspase recruitment domain-containing protein 14	1.77E+04	2452.67
gi|5031595	2 (1)	101.89	7.62E-03	7.27	actin-related protein 2/3 complex subunit 4 isoform a	3580.25	2.60E+04
gi|194381968	4 (3)	169.99	2.09E-03	7.38	unnamed protein product	3.81E+04	5156.15
gi|9802306	1	144.93	0.02	7.44	DNA-binding protein TAXREB107	8211.74	6.11E+04
gi|317455099	1	78.90	2.72E-03	7.55	Chain A, Crystal Structure Of Human Nudt5 Complexed With 8-oxo-dgdp And Manganese	3543.50	2.67E+04
gi|662994	1	42.71	0.02	7.58	GPI-anchored protein p137	1189.53	9019.31
gi|403292972	3 (1)	219.41	3.49E-06	7.59	alpha-actinin-4	1.32E+04	9.99E+04
gi|109107090	2	160.26	1.57E-03	7.60	protein S100-A11	6.73E+04	8852.06
gi|548504378	2 (1)	105.15	3.10E-04	7.62	programmed cell death protein 5	7296.07	5.56E+04
gi|221041758	1	58.88	0.03	7.71	unnamed protein product	195.35	1506.97
gi|219519732	4 (1)	188.06	3.19E-03	7.76	Psma8 protein	8655.02	6.71E+04
gi|73696699	2 (1)	170.08	2.60E-03	7.76	ERp28 protein	3745.45	2.91E+04
gi|74007500	12 (4)	670.05	5.32E-03	7.84	moesin isoformX2	5.82E+04	4.56E+05
gi|65987	12 (1)	646.49	2.00E-03	7.86	glyceraldehyde-3-phosphate dehydrogenase	356.09	2798.92

					(phosphorylating) (EC 1.2.1.12) - pig		
gi|511910713	4 (2)	239.30	4.32E-05	8.04	keratin, type II cytoskeletal 7	1.13E+04	9.10E+04
gi|291395669	8 (1)	488.57	5.02E-03	8.05	histone cluster 1, H2bc-like	2.51E+04	3123.41
gi|426232407	2 (1)	80.25	9.62E-03	8.13	uncharacterized protein LOC101107705	511.71	4157.79
gi|344298464	4 (1)	215.87	0.02	8.21	hypoxanthine-guanine phosphoribosyltransferase-like	7054.38	5.79E+04
gi|528949501	2 (1)	65.73	8.24E-03	8.23	nascent polypeptide-associated complex subunit alpha isoform X1	3567.90	2.94E+04
gi|162813	1	109.45	0.01	8.28	cathepsin B, partial	1.91E+05	2.30E+04
gi|7387724	1	33.81	0.01	8.37	RecName: Full=3-hydroxyacyl-CoA dehydrogenase type-2; AltName: Full=17-beta-hydroxysteroid dehydrogenase 10; Short=17-beta-HSD 10; AltName: Full=3-hydroxy-2-methylbutyryl-CoA dehydrogenase; AltName: Full=3-hydroxyacyl-CoA dehydrogenase type II; AltN	2.11E+04	2517.71
gi|4502565	3 (2)	145.79	0.01	8.48	calpain small subunit 1	1.35E+04	1593.13
gi|444517749	2 (1)	101.37	0.02	8.48	Importin subunit beta-1	512.88	4346.91
gi|478493086	2	105.52	7.21E-03	8.52	LOW QUALITY PROTEIN: ribosomal protein L5	6074.07	5.18E+04
gi|332836687	30 (12)	1484.41	2.52E-03	8.61	neuroblast differentiation-associated protein AHNAK isoform 1	6.46E+04	5.57E+05
gi|345305212	1	37.92	0.05	8.66	coiled-coil domain-containing protein 28A-like	413.59	3581.47
gi|4503477	1	60.45	5.53E-03	8.69	elongation factor 1-beta	2.45E+04	2824.35
gi|27597059	1	47.10	5.65E-04	8.70	dnaJ homolog subfamily C member 9	1317.81	1.15E+04
gi|537129831	6 (1)	346.66	1.45E-04	8.71	tubulin alpha-1B chain	6.92E+04	7941.35
gi|533118782	2 (1)	64.28	8.47E-03	8.72	protein ELYS isoform X1	1751.86	1.53E+04
gi|507662067	2	170.25	1.94E-03	8.80	lupus La protein-like	7.57E+04	8604.94
gi|119597764	1	61.96	0.01	8.80	hCG2045348	1.54E+05	1.75E+04
gi|190192184	11 (9)	645.26	3.09E-04	8.84	filamin A	7.10E+04	6.28E+05
gi|55824584	1	80.84	2.94E-03	8.88	ribosomal protein S5	5382.91	4.78E+04

gi|119604623	4 (1)	194.20	4.15E-03	9.13	protein kinase C substrate 80K-H, isoform CRA_c	7654.17	6.99E+04
gi|194211629	5	242.15	1.54E-03	9.25	triosephosphate isomerase	1.58E+05	1.46E+06
gi|221040626	3 (2)	259.24	1.60E-03	9.32	unnamed protein product	1.28E+05	1.37E+04
gi|1373363	2	77.98	5.55E-04	9.38	platelet-activating factor acetylhydrolase isoform Ib beta subunit	5865.25	5.50E+04
gi|488566468	3 (2)	135.48	3.93E-03	9.44	far upstream element-binding protein 1 isoform 1	2160.91	2.04E+04
gi|7305107	1	47.01	4.64E-06	9.44	protein C10	1745.19	1.65E+04
gi|730529	3	154.46	4.73E-03	9.48	RecName: Full=60S ribosomal protein L13	5856.11	5.55E+04
gi|344240817	1	54.82	9.77E-05	9.48	Dynein heavy chain 5, axonemal	2.82E+05	2.98E+04
gi|402905452	2 (1)	133.03	3.60E-04	9.62	delta(3,5)-Delta(2,4)-dienoyl-CoA isomerase, mitochondrial	3897.26	3.75E+04
gi|537204009	12 (8)	703.80	1.30E-05	9.64	phosphoglycerate mutase 2-like protein	1.39E+05	1.34E+06
gi|343960957	18 (1)	1755.07	0.03	9.75	alpha-enolase	1.29E+04	1.26E+05
gi|126723746	1	61.13	9.71E-04	9.76	serum albumin precursor	1.49E+05	1.53E+04
gi|505779444	2 (1)	79.47	0.02	9.79	fetuin-B-like	3.22E+04	3287.67
gi|90080203	2 (1)	109.85	0.02	9.83	unnamed protein product	2885.73	293.51
gi|238684652	1	57.26	6.22E-03	9.87	MHC class I antigen	4040.19	409.35
gi|2529707	2	124.93	3.54E-04	9.93	Hpast	2.83E+05	2.86E+04
gi|197099610	3	152.01	6.41E-04	9.95	C-1-tetrahydrofolate synthase, cytoplasmic	1.89E+04	1899.15
gi|126291169	2	71.90	7.88E-03	9.95	dihydropyrimidinase-related protein 3	4463.86	4.44E+04
gi|507668095	3	206.63	1.00E-03	10.03	proteasome subunit alpha type-6	1.59E+04	1.60E+05
gi|201723	1	63.99	0.03	10.04	t complex polypeptide 1, partial	692.58	6956.52
gi|488563063	1	44.16	5.30E-05	10.04	glycine N-acyltransferase-like protein 3	6.13E+05	6.11E+04
gi|355748350	7 (1)	439.61	8.77E-04	10.10	Histone H2B.n	6.66E+04	6595.87
gi|431904909	4 (1)	186.64	0.03	10.13	Translationally-controlled tumor protein	1958.68	1.98E+04
gi|390462794	3	169.74	1.42E-03	10.31	60S ribosomal protein L15-like	1.28E+04	1.32E+05
gi|3712663	2	55.87	1.37E-03	10.34	DEAD-box protein	3.32E+04	3210.45
gi|332235675	2 (1)	121.87	9.65E-05	10.46	transcription elongation factor B polypeptide 1-like	3620.51	3.79E+04

gi|57014043	6 (5)	359.96	3.20E-05	10.57	lamin A/C transcript variant 1	4.89E+04	5.17E+05
gi|90075304	3 (2)	189.30	2.16E-04	11.34	unnamed protein product	2.01E+04	2.28E+05
gi|403263579	1	26.51	6.47E-03	11.52	LOW QUALITY PROTEIN: synaptotagmin-like protein 5	2099.90	2.42E+04
gi|524928321	10 (2)	548.64	5.27E-03	11.83	creatine kinase B-type isoform X1	5.12E+04	4326.86
gi|441592694	3 (1)	139.86	3.59E-03	12.10	actin, cytoplasmic type 5-like isoform 1	4012.66	331.72
gi|444721551	2 (1)	82.47	4.40E-04	12.12	60S ribosomal protein L5	5621.77	6.81E+04
gi|306875	8 (5)	527.44	1.26E-04	12.46	C protein	4.96E+05	3.98E+04
gi|62087310	2	130.17	0.01	12.55	gamma filamin variant	1726.41	2.17E+04
gi|332230006	11 (8)	664.66	3.92E-06	12.61	78 kDa glucose-regulated protein isoform 1	3.47E+05	2.75E+04
gi|179832	2 (1)	117.89	4.59E-04	12.74	calnexin	5.42E+04	4250.29
gi|431910380	9 (1)	341.49	3.45E-04	12.77	Neuroblast differentiation-associated protein AHNAK	2758.18	3.52E+04
gi|179531	2	70.28	5.71E-03	13.05	IgE-binding protein	5699.94	7.44E+04
gi|9506761	1	78.29	0.01	13.19	glucosamine 6-phosphate N-acetyltransferase	686.85	9056.35
gi|7023323	1	43.75	1.64E-03	13.21	unnamed protein product	632.35	8352.42
gi|524948880	2 (1)	83.02	7.74E-04	13.23	UDP-glucose 6-dehydrogenase	1.07E+04	806.82
gi|296483028	1	37.93	0.02	13.26	TPA: hCG1994130-like	1198.94	1.59E+04
gi|704416	3	187.54	1.38E-03	13.45	elongation factor Tu	1.55E+05	1.15E+04
gi|512886886	4 (1)	224.23	1.65E-03	13.70	filamin-B isoform X1	1.03E+05	7500.81
gi|297289595	3 (2)	131.18	2.05E-03	13.77	protein disulfide-isomerase A4-like	5.00E+04	3634.80
gi|14249348	1	48.53	0.01	13.77	thioredoxin domain-containing protein 17	1405.57	1.94E+04
gi|223036	2	203.53	5.34E-04	14.23	troponin C-like protein	1.91E+05	1.34E+04
gi|344238877	2 (1)	89.05	0.05	14.26	Transcription factor BTF3	3.45E+04	2416.20
gi|402893095	3 (2)	137.43	1.15E-03	14.31	neutral alpha-glucosidase AB isoform 1	1.32E+04	923.20
gi|4503481	3	174.01	1.70E-03	14.34	elongation factor 1-gamma	2900.57	4.16E+04
gi|533137684	2	113.43	2.04E-04	14.78	60S ribosomal protein L14	1.15E+04	1.70E+05
gi|77702086	20 (15)	1081.18	6.41E-05	14.79	heat shock protein 60	9.35E+05	6.33E+04

gi|4506243	5 (4)	336.93	6.46E-04	14.81	polypyrimidine tract-binding protein 1 isoform a	5.04E+05	3.41E+04
gi|12963573	1	62.62	1.95E-03	14.82	dCTP pyrophosphatase 1	3.77E+04	2543.37
gi|478519122	2 (1)	115.80	1.78E-03	15.01	ribosome-binding protein 1	5355.28	8.04E+04
gi|114052298	1	69.34	9.23E-04	15.04	apolipoprotein A-II precursor	1.29E+04	1.94E+05
gi|4506743	1	80.03	0.03	15.10	40S ribosomal protein S8	1.97E+04	1302.03
gi|32363191	3 (1)	134.08	9.46E-04	15.70	RecName: Full=Merlin; AltName: Full=Moesin-ezrin-radixin-like protein; AltName: Full=Neurofibromin-2; AltName: Full=Schwannomin	3755.46	5.90E+04
gi|504172774	6 (3)	364.38	4.46E-06	15.76	elongation factor 2	2.18E+05	1.38E+04
gi|494066	2	159.04	3.13E-04	15.93	Chain A, Three-Dimensional Structure Of Class Pi Glutathione S-Transferase From Human Placenta In Complex With S-Hexylglutathione At 2.8 Angstroms Resolution	3.19E+04	5.08E+05
gi|262391	1	68.50	8.19E-03	16.21	Rig homolog [human, brain, Peptide Partial, 135 aa]	2.54E+04	1565.66
gi|74267962	5 (1)	280.03	1.64E-04	16.34	ALB protein	7.23E+04	4426.83
gi|22538467	2	194.49	3.95E-04	16.37	proteasome subunit beta type-4	1.56E+05	9556.00
gi|53035	1	71.16	5.09E-04	16.70	cyclophilin CyP-S1	1.24E+05	7407.69
gi|444523630	4	194.22	3.02E-04	16.73	NSFL1 cofactor p47	1.67E+04	2.79E+05
gi|297412	1	38.30	2.53E-03	16.84	thrombin inhibitor	559.75	9427.84
gi|436226	2	106.12	2.25E-03	16.94	KIAA0038	4.73E+04	2792.54
gi|426386401	1	99.25	5.11E-03	16.96	calponin-2 isoform 4	9388.26	1.59E+05
gi|478529796	3 (1)	69.15	0.02	17.09	kinesin-like protein KIF21B	4340.69	253.95
gi|28077049	1	96.15	9.58E-05	17.18	charged multivesicular body protein 4b	7306.85	1.26E+05
gi|7657603	1	41.35	1.54E-03	17.31	heme-binding protein 2	1.96E+04	1130.64
gi|28277147	1	64.54	2.58E-03	17.40	Metadherin	5115.40	293.98
gi|465987982	1	28.33	2.46E-03	17.43	protein FAM198B	1.36E+04	2.36E+05
gi|160964410	3 (1)	123.03	5.21E-03	17.47	transferrin receptor 1	6749.21	386.30

gi|511864079	15 (2)	816.61	1.87E-04	17.48	tubulin beta-3 chain	1105.23	1.93E+04
gi|296472298	5 (1)	171.67	8.49E-03	17.56	TPA: histone cluster 1, H2bd-like	1.02E+05	5782.75
gi|5031512	2 (1)	91.80	9.31E-04	17.66	NSAP1 protein	2.66E+04	1507.41
gi|351698607	1	41.71	7.23E-03	17.73	CLIP-associating protein 1	2601.00	146.67
gi|39777597	2	187.48	1.36E-03	18.03	protein-glutamine gamma-glutamyltransferase 2 isoform a	1.75E+04	3.15E+05
gi|544430751	20 (7)	1259.04	2.04E-03	18.05	uncharacterized protein LOC102118097	2.18E+05	1.21E+04
gi|162678	2	135.99	3.56E-03	18.07	apolipoprotein A-I precursor	7.58E+04	4196.20
gi|511846280	5 (4)	243.59	2.22E-05	18.09	transitional endoplasmic reticulum ATPase isoform X1	8.59E+04	4747.12
gi|466023766	4 (1)	211.79	0.04	18.10	adenosylhomocysteinase isoform 1	3218.44	177.79
gi|7710086	1	72.80	2.42E-03	18.11	ras-related protein Rab-10	714.88	1.29E+04
gi|297272999	2	175.24	3.27E-04	18.25	hypothetical protein LOC706830 isoform 3	547.36	9990.31
gi|441670702	3 (2)	206.42	8.26E-05	18.35	LOW QUALITY PROTEIN: peptidyl-prolyl cis-trans isomerase FKBP4	4.80E+04	2617.03
gi|1263196	5	297.76	1.57E-05	18.85	AICAR formyltransferase/IMP cyclohydrolase bifunctional enzyme	2.18E+05	1.16E+04
gi|5031635	6 (4)	355.61	3.06E-04	18.90	cofilin-1	3.56E+05	1.88E+04
gi|20067392	3 (2)	131.28	1.27E-04	19.14	thioredoxin related protein	1999.56	3.83E+04
gi|33239320	1	50.94	9.26E-03	19.40	olfactory receptor 96	3009.04	5.84E+04
gi|291384816	2 (1)	74.26	0.01	19.58	catalase	5981.90	305.50
gi|55727871	2 (1)	90.61	2.70E-03	20.06	hypothetical protein	2098.54	4.21E+04
gi|187609338	2 (1)	101.92	2.20E-03	20.50	Chain A, Crystal Structure Of The Extracellular Portion Of Hab18gCD147	2.73E+04	1330.31
gi|533125814	7 (1)	350.85	2.58E-03	20.53	radixin isoform X3	3.61E+04	1756.14
gi|21730367	1	104.46	3.36E-03	20.58	Chain A, Ca2+-Binding Mimicry In The Crystal Structure Of The Eu3+-Bound Mutant Human Macrophage Capping Protein Cap G	2698.80	5.56E+04
gi|6755728	1	85.53	4.55E-03	20.90	transcription elongation factor A protein 1 isoform 2	3.34E+04	1598.53

gi|16306978	14 (3)	790.45	7.60E-05	21.01	Annexin A2	1.49E+04	3.13E+05
gi|4502303	2	146.03	2.75E-03	21.18	ATP synthase subunit O, mitochondrial precursor	1.03E+05	4853.06
gi|4092058	4 (1)	198.06	0.03	21.46	proteasome subunit HSPC	617.01	1.32E+04
gi|46249758	10 (1)	596.31	7.66E-05	21.60	Ezrin	8900.36	1.92E+05
gi|4506597	6 (5)	396.94	8.64E-04	21.72	60S ribosomal protein L12	1.56E+05	7200.75
gi|392354114	6 (2)	268.01	6.01E-03	22.39	LOW QUALITY PROTEIN: ezrin-like	1.02E+05	4562.93
gi|297689148	3 (1)	161.13	3.13E-05	22.63	L-lactate dehydrogenase A-like 6A	4.75E+04	2098.28
gi|3088342	1	80.07	0.02	22.69	ribosomal protein S23	538.68	1.22E+04
gi|537227169	9 (1)	518.71	7.11E-06	22.79	glyceraldehyde-3-phosphate dehydrogenase	5883.52	1.34E+05
gi|4507401	1	53.40	9.76E-03	22.82	transcription factor A, mitochondrial isoform 1 precursor	1.13E+04	496.75
gi|545178330	2 (1)	71.23	2.46E-04	22.84	LOW QUALITY PROTEIN: Na(+)/H(+) exchange regulatory cofactor NHE-RF1	3.54E+04	1548.67
gi|209838088	1	81.30	2.03E-04	23.03	glutathione synthetase	1698.38	3.91E+04
gi|291387421	5 (3)	243.35	3.43E-03	23.71	heat shock 70kDa protein 9	4.22E+04	1778.19
gi|119581823	2	68.47	1.50E-04	23.91	hCG1979282	1985.59	4.75E+04
gi|441620457	16 (1)	978.87	4.50E-03	23.94	tubulin alpha-1C chain-like	1850.94	4.43E+04
gi|19527026	1	65.04	0.02	23.94	leucine-rich repeat-containing protein 59	448.34	1.07E+04
gi|470632809	2 (1)	76.81	4.62E-04	24.06	LOW QUALITY PROTEIN: insulin receptor substrate 1-like [Tursiops truncatus]	1.73E+04	718.65
gi|156766050	2 (1)	88.41	0.01	24.35	protein AHNAK2	642.13	1.56E+04
gi|511881761	2 (1)	73.78	2.22E-03	24.46	cytochrome b-c1 complex subunit 1, mitochondrial-like isoform X1	2.58E+04	1054.03
gi|312005	1	55.46	8.16E-05	25.03	small nuclear ribonucleoprotein E	3573.16	8.94E+04
gi|6014493	2 (1)	143.72	3.52E-04	25.15	PLIC-2	1899.31	4.78E+04
gi|12082136	19 (5)	1073.58	1.61E-05	26.58	heat shock protein 90 alpha	1.83E+05	6881.01
gi|108967	1	55.54	8.73E-04	27.23	ubiquitin carboxyl-terminal proteinase - bovine (fragments)	2948.66	8.03E+04
gi|351713549	2	100.76	3.17E-03	27.41	Prohibitin	1.18E+05	4322.03

gi|4757810	5 (4)	388.44	9.21E-05	28.05	ATP synthase subunit alpha, mitochondrial isoform a precursor	2.36E+05	8412.11
gi|9507245	4 (1)	200.64	2.23E-03	28.24	14-3-3 protein gamma	1.57E+04	557.06
gi|73760405	1	68.22	2.23E-03	29.61	thymopoietin isoform beta	2.89E+04	976.02
gi|537271418	3 (1)	120.19	7.25E-03	29.98	chromodomain-helicase-DNA-binding protein 1	668.83	22.31
gi|62460494	2 (1)	228.13	0.01	30.18	hemoglobin fetal subunit beta	3853.27	1.16E+05
gi|332250529	2 (1)	61.52	5.32E-03	30.25	uncharacterized protein C5orf42 homolog	5.24E+04	1732.18
gi|13775198	1	76.10	9.94E-03	30.86	SH3 domain-binding glutamic acid-rich-like protein 3	3080.25	9.51E+04
gi|444721795	6 (1)	228.92	0.04	31.16	ATP-binding cassette sub-family A member 3	114.45	3565.94
gi|1351907	6 (1)	334.89	7.42E-04	31.17	RecName: Full=Serum albumin; AltName: Full=BSA; AltName: Allergen=Bos d 6; Flags: Precursor	1.84E+04	590.71
gi|431914265	2 (1)	125.64	2.67E-03	32.11	Paxillin	3758.75	1.21E+05
gi|397466849	5 (2)	238.59	1.02E-04	32.39	keratin, type II cytoskeletal 8-like	3070.43	9.95E+04
gi|187302	3 (1)	134.89	9.89E-03	32.75	epithelial cell marker protein 1	1280.32	4.19E+04
gi|4757900	3	87.06	2.75E-05	32.76	calreticulin precursor	9.25E+04	2822.22
gi|444725708	8 (1)	394.42	4.07E-04	32.86	60S ribosomal protein L7a	5.04E+04	1533.32
gi|545521016	1	31.07	0.01	33.63	maestro heat-like repeat family member 5 isoform X1	2.94E+04	875.29
gi|194389534	1	28.19	0.05	34.21	unnamed protein product	4468.64	130.63
gi|351701782	1	75.84	3.62E-03	35.36	Protein S100-A10	1.87E+04	527.65
gi|4502013	5 (1)	230.10	2.11E-03	36.09	adenylate kinase 2, mitochondrial isoform a	618.00	2.23E+04
gi|441669563	6 (3)	464.53	8.17E-04	37.25	nucleolin	2.66E+05	7147.78
gi|532081396	2 (1)	65.82	5.47E-04	37.62	collagen alpha-3(VI) chain [Ictidomys tridecemlineatus]	760.89	2.86E+04
gi|47847498	4 (1)	194.46	6.06E-03	37.89	mFLJ00279 protein	100.25	3798.12
gi|12653493	2 (1)	115.82	6.95E-04	38.48	Brain abundant, membrane attached signal protein 1	4851.17	1.87E+05
gi|62897945	19 (1)	1820.45	1.50E-04	38.57	enolase 1 variant	1.05E+04	4.04E+05

gi|7739445	2	140.29	7.15E-03	38.69	hnRNP 2H9B	3288.72	85.00
gi|197304975	2 (1)	84.05	7.81E-04	39.30	Chain A, Oxidized And Reduced Forms Of Human Peroxiredoxin 5	2.88E+04	733.25
gi|537141603	2	97.46	2.17E-04	40.03	sequestosome-1	4.67E+04	1166.39
gi|4502297	1	70.57	5.28E-03	40.85	ATP synthase subunit delta, mitochondrial precursor	1.71E+04	419.01
gi|507653930	2 (1)	137.16	1.44E-04	41.42	translation initiation factor IF-2-like	1927.96	7.99E+04
gi|390462052	5 (1)	222.63	2.26E-04	42.14	tropomyosin alpha-3 chain-like	1.08E+04	255.23
gi|77736017	1	59.51	1.21E-03	42.24	NAD(P)H dehydrogenase [quinone] 1	234.47	9904.77
gi|410968032	2 (1)	100.57	1.35E-04	42.57	ATP synthase subunit b, mitochondrial [Felis catus]	9.43E+04	2216.09
gi|403265302	2 (1)	153.04	0.03	48.06	T-complex protein 1 subunit beta-like	102.20	4911.53
gi|109465383	2 (1)	125.48	0.03	48.76	60S acidic ribosomal protein P0-like	57.49	2803.31
gi|1022961	3 (1)	143.94	8.58E-05	49.28	HuR RNA binding protein	367.36	1.81E+04
gi|347964	1	49.97	6.93E-03	49.58	TARBP-b, partial	3.22E+04	649.84
gi|28204689	1	56.89	6.40E-04	50.03	ribosomal protein S4 [Pongo pygmaeus]	4.45E+04	889.73
gi|7022407	1	71.52	2.08E-03	50.14	unnamed protein product	4.36E+04	868.90
gi|307109	1	81.94	3.15E-03	50.90	lysosomal membrane glycoprotein-1	1386.02	7.05E+04
gi|478490666	1	34.84	0.05	55.28	three prime repair exonuclease 1-like isoform 1	137.95	7625.62
gi|488538974	1	39.76	1.21E-04	55.80	probable asparagine--tRNA ligase, mitochondrial isoform 1	2.98E+05	5347.14
gi|114665952	6 (4)	288.19	1.12E-04	56.44	complement component 1 Q subcomponent-binding protein, mitochondrial isoform 6	1.77E+05	3131.31
gi|119625664	9 (1)	644.03	3.27E-04	56.88	annexin A5, isoform CRA_c	1373.63	7.81E+04
gi|114558626	4 (1)	198.75	8.40E-04	57.90	translationally-controlled tumor protein-like isoform 7	1159.64	6.71E+04
gi|42543459	2 (1)	57.10	0.03	62.18	Chain A, Crystal Structure Of A Mu-Like Calpain	152.09	9457.16
gi|55732000	21 (5)	1488.99	4.16E-05	64.39	hypothetical protein	1.02E+06	1.58E+04
gi|533203458	1	40.28	7.49E-04	65.54	LOW QUALITY PROTEIN: deoxyribonuclease-1-like 1	1.84E+04	281.21
gi|1552242	2 (1)	127.73	3.85E-03	71.35	hRlf beta subunit (p102 protein)	15.83	1129.33

gi|440909513	2 (1)	127.95	0.02	72.35	Paxillin, partial [Bos grunniens mutus]	1542.49	21.32
gi|11034825	1	101.63	3.16E-03	73.74	methionine adenosyltransferase 2 subunit beta isoform 1	188.47	1.39E+04
gi|10140853	1	136.15	2.96E-04	76.04	acyl-CoA-binding protein isoform 1	3.22E+05	4239.30
gi|529281076	2 (1)	122.59	2.01E-04	82.59	Chain A, Crystal Structure Of Rat Galectin-1 In Complex With Lactose	3442.81	2.84E+05
gi|392344177	1	33.45	2.10E-04	83.80	zinc finger protein 850-like	8.29E+04	989.34
gi|471382122	1	27.73	5.09E-04	84.18	retinal-specific ATP-binding cassette transporter [Trichechus manatus latirostris]	1.88E+05	2233.64
gi|4506699	1	54.65	7.77E-04	85.50	40S ribosomal protein S21	6.17E+04	721.13
gi|253706	6 (4)	382.85	2.48E-04	89.90	14-3-3 protein zeta chain	2.53E+05	2812.70
gi|358419864	1	39.08	5.41E-06	90.73	mucin-19	7910.88	7.18E+05
gi|537153540	4 (2)	271.81	1.45E-04	91.67	bromodomain adjacent to zinc finger domain protein 1A	1995.83	1.83E+05
gi|3088338	1	63.41	2.13E-04	96.23	ribosomal protein S10	373.97	3.60E+04
gi|297296973	2 (1)	122.89	0.04	97.26	reticulocalbin-2	1.74E+04	178.65
gi|465991859	2 (1)	101.22	0.03	113.16	serine hydroxymethyltransferase, cytosolic isoform 1	148.83	1.68E+04
gi|301770723	6 (1)	303.08	0.05	124.39	LOW QUALITY PROTEIN: elongation factor 1-alpha, oocyte form-like	6.13E+04	492.78
gi|74152226	2	141.86	4.37E-04	124.39	unnamed protein product	1692.62	2.11E+05
gi|109122464	4 (1)	186.83	1.86E-04	132.70	l-lactate dehydrogenase B chain	8.63E+04	650.28
gi|544480052	1	39.32	0.02	135.66	zinc finger protein 415-like	1.08E+04	79.26
gi|472391178	3 (1)	140.25	0.03	140.69	T-complex protein 1 subunit epsilon isoform 2	2.59E+04	184.40
gi|10120888	3 (1)	132.85	1.19E-05	143.30	Chain A, The Crystal Structure Of Human Barrier-To-Autointegration Factor	997.32	1.43E+05
gi|5453559	2 (1)	173.48	7.17E-04	167.68	ATP synthase subunit d, mitochondrial isoform a	2.12E+05	1261.72
gi|148681385	3 (2)	156.07	0.02	169.71	succinate dehydrogenase complex, subunit B, iron sulfur (Ip), isoform CRA_b	3.93E+04	231.84

gi|17933772	1	78.03	6.28E-05	207.93	protein S100-A16	217.36	4.52E+04
gi|4758762	1	44.16	6.02E-03	221.64	asparagine--tRNA ligase, cytoplasmic	3.37	747.90
gi|348554675	9 (1)	471.96	0.02	231.16	creatine kinase B-type isoform X1	8.78E+04	379.76
gi|32425649	1	46.16	0.01	232.43	EIF4B protein, partial	46.11	1.07E+04
gi|126305744	2 (1)	72.87	6.59E-03	244.69	UMP-CMP kinase-like	156.68	3.83E+04
gi|505785940	1	51.76	0.01	290.86	protein shisa-2 homolog	38.87	1.13E+04
gi|35959	14 (2)	899.56	0.01	334.01	tubulin 5-beta	3.48E+04	104.34
gi|251370	1	70.85	3.05E-03	364.06	acid phosphatase isoenzyme Af	6.64	2416.73
gi|9955206	1	50.21	3.66E-03	421.36	Chain B, Crystal Structure Of A Rac-Rhogdi Complex	1.09E+05	257.53
gi|332820815	2 (1)	102.03	0.01	518.39	succinate dehydrogenase [ubiquinone] flavoprotein subunit, mitochondrial-like isoform 1	3.24E+04	62.42
gi|507541388	1	35.75	5.08E-03	934.69	coiled-coil domain-containing protein 102B	7931.64	8.49
gi|344256722	1	71.08	5.57E-03	941.74	High mobility group protein 1-like 10	2.28E+04	24.21
gi|544402295	2 (1)	120.19	3.00E-03	1002.84	interleukin enhancer-binding factor 2 isoform X1	7.31	7329.63
gi|7709912	1	53.33	6.91E-04	1071.42	phosphoglucomutase 1	1.49	1599.26
gi|348567745	2	74.04	2.75E-03	1210.53	T-complex protein 1 subunit zeta-2 isoformX1	1.27E+04	10.46
gi|524968854	2 (1)	99.52	3.54E-03	1287.50	acidic leucine-rich nuclear phosphoprotein 32 family member E isoform X1	8771.86	6.81
gi|444721584	2 (1)	65.49	1.45E-04	4022.81	Heterogeneous nuclear ribonucleoprotein D-like protein	3524.06	0.88
gi|4558043	1	138.13	1.42E-03	4716.05	Chain B, Structure Of Nedd8	41.79	1.97E+05
gi|4504253	6 (1)	423.58	2.57E-03	1.58E+04	histone H2AX	4232.37	0.27
gi|56606098	1	36.63	1.22E-05	1.61E+04	complement C1q subcomponent subunit C precursor	0.22	3503.93
gi|126340084	3 (1)	126.32	4.00E-05	Infinity	prohibitin-2-like	1305.74	0.00
gi|432092846	2 (1)	123.83	1.59E-06	Infinity	Paxillin	0.00	1216.20
gi|507635704	3 (1)	108.57	5.73E-05	Infinity	60S ribosomal protein L4, partial	2.35E+04	0.00
gi|466045811	2 (1)	98.24	4.01E-07	Infinity	lon protease homolog, mitochondrial	0.00	551.23

gi|193787759	1	77.87	4.60E-04	Infinity	unnamed protein product	1600.53	0.00
gi|395857161	2 (1)	76.35	5.39E-08	Infinity	zinc finger protein 185 isoform 5	0.00	1.15E+04
gi|296215020	2 (1)	73.50	6.68E-09	Infinity	small nuclear ribonucleoprotein-associated protein N-like	0.00	4790.34
gi|126343505	2 (1)	72.05	2.44E-03	Infinity	structural maintenance of chromosomes protein 6-like	2109.61	0.00
gi|532057143	2 (1)	69.65	8.93E-05	Infinity	nascent polypeptide-associated complex subunit alpha, muscle-specific form	0.00	354.98
gi|1706754	1	49.06	6.99E-07	Infinity	Fatty acid-binding protein, epidermal	0.00	1301.86
gi|26336162	1	45.86	8.20E-07	Infinity	unnamed protein product	1.92E+05	0.00
gi|6225527	1	44.24	4.14E-08	Infinity	Isopentenyl-diphosphate Delta-isomerase 1	0.00	2366.24
gi|472389073	1	41.39	1.76E-05	Infinity	retinoic acid receptor responder protein 3	2807.75	0.00
gi|28461243	1	36.61	2.24E-04	Infinity	histidine triad nucleotide-binding protein 1	5850.43	0.00

***** significant expression : $p<0.05$.

Table S4. Ingenuity canonical pathway analysis of mapped differentially expressed proteins between PC-3 and PC-3RR cell lines

Ingenuity Canonical Pathways	-log(p-value)	Ratio	Molecules
Aldosterone Signaling in Epithelial Cells	5.93E00	5.56E-02	HSP90B1,HSP90AB1,PDIA3,DNAJB11,HSPE1,HSPA1A/HSPA1B,DNAJC3,HSPD1,AHCY
Protein Ubiquitination Pathway	3.52E00	3.09E-02	HSP90B1,HSP90AB1,DNAJB11,HSPE1,HSPA1A/HSPA1B,DNAJC3,PSMB8,HSPD1
Unfolded protein response	3.38E00	7.41E-02	P4HB,HSP90B1,HSPA1A/HSPA1B,DNAJC3
14-3-3-mediated Signaling	2.96E00	4.2E-02	YWHAQ,YAP1,YWHAE,PDIA3,VIM
HIPPO signaling	2.6E00	4.6E-02	YWHAQ,YAP1,YWHAE,CD44
Pentose Phosphate Pathway (Non-oxidative Branch)	2.51E00	1.54E-01	TKT,TKTL1
Regulation of Cellular Mechanics by Calpain Protease	2.19E00	5.17E-02	CAPNS1,VCL,ACTN4
Oxidative Phosphorylation	2.1E00	3.33E-02	ATP5H,UQCRC2,ATP5I,NDUFA8
Mitochondrial Dysfunction	2.1E00	2.65E-02	PRDX3,ATP5H,UQCRC2,ATP5I,NDUFA8
Glucocorticoid Receptor Signaling	2.09E00	2.25E-02	HSP90B1,HSP90AB1,HSPA1A/HSPA1B,CSN2,POLR2K,NCOA3
Endoplasmic Reticulum Stress Pathway	2.09E00	9.52E-02	HSP90B1,DNAJC3
Hypoxia Signaling in the Cardiovascular System	2.06E00	4.62E-02	P4HB,HSP90B1,HSP90AB1
p70S6K Signaling	2.04E00	3.2E-02	YWHAQ,YWHAE,PDIA3,EEF2
Pentose Phosphate Pathway	2.01E00	8.7E-02	TKT,TKTL1

PI3K/AKT Signaling	2.01E00	3.12E-02	YWHAQ,HSP90B1,YWHAE,HSP90AB1
Remodeling of Epithelial Adherens Junctions	2E00	4.41E-02	ZYX,VCL,ACTN4
Integrin Signaling	1.98E00	2.48E-02	CAPNS1,CAV1,ZYX,VCL,ACTN4
Aryl Hydrocarbon Receptor Signaling	1.82E00	2.74E-02	HSP90B1,HSP90AB1,GSTP1,NCOA3
eNOS Signaling	1.74E00	2.6E-02	HSP90B1,HSP90AB1,HSPA1A/HSPA1B,CAV1
Prostate Cancer Signaling	1.73E00	3.49E-02	HSP90B1,HSP90AB1,GSTP1
Antigen Presentation Pathway	1.62E00	5.41E-02	PDIA3,PSMB8
Virus Entry via Endocytic Pathways	1.62E00	3.16E-02	CLTC,CAV1,TFRC
VEGF Signaling	1.59E00	3.09E-02	YWHAE,VCL,ACTN4
Glycolysis I	1.54E00	4.88E-02	PGAM2,ALDOA
PPARα/RXRα Activation	1.49E00	2.17E-02	HSP90B1,HSP90AB1,PDIA3,NCOA3
EIF2 Signaling	1.46E00	2.13E-02	RPS28,RPLP1,RPL3,RPS21
Gluconeogenesis I	1.44E00	4.35E-02	PGAM2,ALDOA
Nitric Oxide Signaling in the Cardiovascular System	1.44E00	2.68E-02	HSP90B1,HSP90AB1,CAV1
Cell Cycle: G2/M DNA Damage Checkpoint Regulation	1.39E00	4.08E-02	YWHAQ,YWHAE

Table S5. Ingenuity canonical pathway analysis of mapped differentially expressed proteins between DU145 and DU145RR cell lines

**Ingenuity Canonical Pathways**	**-log (p-value)**	**Ratio**	**Molecules**
EIF2 Signaling	8.47E00	6.91E-02	RPS7,PABPC1,RPL6,RPL3,RPS9,RPS10,RPS8,RPL12,RPS21,RPS3,RPS23,RPL13,RPS14
Regulation of eIF4 and p70S6K Signaling	5.51E00	6E-02	PABPC1,RPS7,RPS9,RPS10,RPS8,RPS21,RPS3,RPS23,RPS14
mTOR Signaling	4.61E00	4.64E-02	RPS7,RPS9,RPS10,RPS8,RPS21,RPS3,RPS23,EIF4B,RPS14
Oxidative Phosphorylation	4.35E00	5.88E-02	SDHA,SDHB,ATP5O,ATP5H,ATP5D,ATP5A1,COX4I1
Mitochondrial Dysfunction	3.89E00	4.26E-02	HSD17B10,SDHA,SDHB,ATP5O,ATP5H,ATP5D,ATP5A1,COX4I1
14-3-3-mediated Signaling	3.43E00	5.04E-02	YWHAG,YWHAE,PDIA3,VIM,TUBB4A,SFN
Actin Cytoskeleton Signaling	3.42E00	3.62E-02	MYH9,CFL1,FLNA,EZR,GSN,ARPC4,MSN,FGF6
Acute Phase Response Signaling	3.37E00	4.09E-02	ALB,APOA1,TF,ITIH2,APOA2,AHSG,SERPINA1
LXR/RXR Activation	3.26E00	4.69E-02	ALB,APOA1,TF,APOA2,AHSG,SERPINA1
Clathrin-mediated Endocytosis Signaling	3.12E00	3.72E-02	ALB,APOA1,TF,APOA2,SERPINA1,ARPC4,FGF6
FXR/RXR Activation	3.09E00	4.35E-02	ALB,APOA1,TF,APOA2,AHSG,SERPINA1
Lipid Antigen Presentation by CD1	2.96E00	1.15E-01	CALR,PDIA3,CANX
Caveolar-mediated Endocytosis Signaling	2.55E00	5.41E-02	ALB,FLNA,FLNC,PTRF
Atherosclerosis Signaling	2.51E00	4.03E-02	ALB,APOA1,APOA2,SERPINA1,PRDX6
Antigen Presentation Pathway	2.51E00	8.11E-02	CALR,PDIA3,CANX
PI3K/AKT Signaling	2.46E00	3.91E-02	CDC37,YWHAG,YWHAE,HSP90AB1,SFN
TCA Cycle II (Eukaryotic)	2.38E00	7.32E-02	SDHA,SDHB,DLD

Glycolysis I	2.38E00	7.32E-02	PGK1,ENO1,ALDOA
HIPPO signaling	2.3E00	4.6E-02	YWHAG,YWHAE,NF2,SFN
Gluconeogenesis I	2.24E00	6.52E-02	PGK1,ENO1,ALDOA
Cell Cycle: G2/M DNA Damage Checkpoint Regulation	2.17E00	6.12E-02	YWHAG,YWHAE,SFN
Myc Mediated Apoptosis Signaling	1.96E00	5.17E-02	YWHAG,YWHAE,SFN
ERK5 Signaling	1.85E00	4.69E-02	YWHAG,YWHAE,SFN
Hypoxia Signaling in the Cardiovascular System	1.83E00	4.62E-02	HSP90AB1,NQO1,LDHA
Production of Nitric Oxide and Reactive Oxygen Species in Macrophages	1.79E00	2.69E-02	ALB,APOA1,APOA2,CYBA,SERPINA1
Remodeling of Epithelial Adherens Junctions	1.78E00	4.41E-02	MAPRE1,TUBB4A,ARPC4
ILK Signaling	1.77E00	2.66E-02	MYH9,CFL1,FLNA,FLNC,VIM
RhoA Signaling	1.77E00	3.23E-02	CFL1,EZR,ARPC4,MSN
Protein Ubiquitination Pathway	1.77E00	2.32E-02	PSMB3,PSMB4,DNAJC9,HSP90AB1,PSMA7,HSPD1
p70S6K Signaling	1.76E00	3.2E-02	YWHAG,YWHAE,PDIA3,SFN
Isoleucine Degradation I	1.66E00	6.9E-02	HSD17B10,DLD
IL-12 Signaling and Production in Macrophages	1.61E00	2.88E-02	ALB,APOA1,APOA2,SERPINA1
Complement System	1.44E00	5.26E-02	C1QC,C1QBP
Regulation of Actin-based Motility by Rho	1.43E00	3.23E-02	CFL1,GSN,ARPC4
Huntington's Disease Signaling	1.4E00	2.12E-02	SDHA,TGM2,PSMA8,SDHB,CAPNS1

Signaling by Rho Family GTPases	1.4E00	2.12E-02	CFL1,EZR,VIM,ARPC4,MSN
Aldosterone Signaling in Epithelial Cells	1.4E00	2.47E-02	DNAJC9,HSP90AB1,PDIA3,HSPD1
VEGF Signaling	1.38E00	3.09E-02	YWHAE,SFN,ELAVL1
Purine Nucleotides De Novo Biosynthesis II	1.36E00	4.76E-02	IMPDH2,ATIC
IGF-1 Signaling	1.36E00	3.03E-02	YWHAG,YWHAE,SFN
Glycine Biosynthesis I	1.33E00	1.67E-01	SHMT2

Table S6. Ingenuity canonical pathway analysis of mapped differentially expressed proteins between LNCaP and LNCaPRR cell lines

**Ingenuity Canonical Pathways**	**-log (p-value)**	**Ratio**	**Molecules**
EIF2 Signaling	7.92E00	6.38E-02	RPS7,RPL6,RPL3,RPS9,RPS10,RPS8,RPS21,RPL12,RPS3,RPS23,RPL13,RPS14
mTOR Signaling	4.93E00	4.64E-02	RPS7,RPS9,RPS10,RPS8,RPS21,RPS3,RPS23,EIF4B,RPS14
Regulation of eIF4 and p70S6K Signaling	4.88E00	5.33E-02	RPS7,RPS9,RPS10,RPS8,RPS21,RPS3,RPS23,RPS14
Actin Cytoskeleton Signaling	3.69E00	3.62E-02	MYH9,CFL1,FLNA,EZR,GSN,ARPC4,MSN,FGF6
Oxidative Phosphorylation	3.65E00	5.04E-02	SDHA,SDHB,ATP5O,ATP5H,ATP5D,ATP5A1
Mitochondrial Dysfunction	3.36E00	3.72E-02	HSD17B10,SDHA,SDHB,ATP5O,ATP5H,ATP5D,ATP5A1
Acute Phase Response Signaling	2.82E00	3.51E-02	ALB,APOA1,TF,ITIH2,APOA2,AHSG
14-3-3-mediated Signaling	2.77E00	4.2E-02	YWHAG,YWHAE,VIM,TUBB4A,SFN
Caveolar-mediated Endocytosis Signaling	2.7E00	5.41E-02	ALB,FLNA,FLNC,PTRF
LXR/RXR Activation	2.63E00	3.91E-02	ALB,APOA1,TF,APOA2,AHSG
Clathrin-mediated Endocytosis Signaling	2.61E00	3.19E-02	ALB,APOA1,TF,APOA2,ARPC4,FGF6
TCA Cycle II (Eukaryotic)	2.5E00	7.32E-02	SDHA,SDHB,DLD
FXR/RXR Activation	2.49E00	3.62E-02	ALB,APOA1,TF,APOA2,AHSG
HIPPO signaling	2.44E00	4.6E-02	YWHAG,YWHAE,NF2,SFN
Cell Cycle: G2/M DNA Damage Checkpoint Regulation	2.28E00	6.12E-02	YWHAG,YWHAE,SFN
Myc Mediated Apoptosis Signaling	2.07E00	5.17E-02	YWHAG,YWHAE,SFN
ERK5 Signaling	1.96E00	4.69E-02	YWHAG,YWHAE,SFN
ILK Signaling	1.93E00	2.66E-02	MYH9,CFL1,FLNA,FLNC,VIM

RhoA Signaling	1.91E00	3.23E-02	CFL1,EZR,ARPC4,MSN
Atherosclerosis Signaling	1.91E00	3.23E-02	ALB,APOA1,APOA2,PRDX6
PI3K/AKT Signaling	1.86E00	3.12E-02	CDC37,YWHAG,YWHAE,SFN
Lipid Antigen Presentation by CD1	1.83E00	7.69E-02	CALR,CANX
Isoleucine Degradation I	1.74E00	6.9E-02	HSD17B10,DLD
Huntington's Disease Signaling	1.55E00	2.12E-02	SDHA,TGM2,PSMA8,SDHB,CAPNS1
Signaling by Rho Family GTPases	1.55E00	2.12E-02	CFL1,EZR,VIM,ARPC4,MSN
Antigen Presentation Pathway	1.54E00	5.41E-02	CALR,CANX
Regulation of Actin-based Motility by Rho	1.53E00	3.23E-02	CFL1,GSN,ARPC4
Complement System	1.52E00	5.26E-02	C1QC,C1QBP
VEGF Signaling	1.48E00	3.09E-02	YWHAE,SFN,ELAVL1
IGF-1 Signaling	1.46E00	3.03E-02	YWHAG,YWHAE,SFN
Glycolysis I	1.45E00	4.88E-02	ENO1,ALDOA
RhoGDI Signaling	1.39E00	2.23E-02	CFL1,EZR,ARPC4,MSN
NRF2-mediated Oxidative Stress Response	1.38E00	2.22E-02	ERP29,DNAJC9,PPIB,NQO1
Glycine Biosynthesis I	1.37E00	1.67E-01	SHMT2
Gluconeogenesis I	1.36E00	4.35E-02	ENO1,ALDOA
Production of Nitric Oxide and Reactive Oxygen Species in Macrophages	1.34E00	2.15E-02	ALB,APOA1,APOA2,CYBA

Table S7. Disease and function analysis of mapped differentially expressed proteins between PC-3 and PC-3RR cell lines

**Categories**	**Diseases or Functions Annotation**	**p-Value**	**Molecules**	**Molecules number**
Cellular Growth and Proliferation	proliferation of cells	1.72E-08	ACTN4,AHCY,AHNAK,AHSG,ALDOA,ANXA7,CAPNS1,CAV1,CD44,CLTC,DNAJC3,EEF1A1,EIF4B,EIF4E3,ERO1L,FABP5,FZD4,GALNT2,GSTP1,HINT1,HMGA1,HNRNPA1,HNRNPAB,HNRNPK,HSP90AB1,HSP90B1,HSPA1A/HSPA1B,HSPD1,IL27,KRT7,KRT8,LASP1,LGALS3,MANF,MARCKSL1,NCOA3,NPM1,PCDH1,PDIA3,PFN1,PGAM2,POLR2K,PRDX3,PRDX4,RTN4,S100A6,SCAMP3,scopolamine,SF1,SLC12A6,SRSF1,SRSF3,STMN1,TCOF1,TFG,TFRC,THBS1,TIMM8A,TPD52L2,VCL,VIM,YAP1,YBX1,YWHAQ,ZYX	65
Hematological Disease, Immunological Disease, Inflammatory Disease, Inflammatory Response, Respiratory Disease	allergic pulmonary eosinophilia	5.34E-08	ALDOA,HNRNPAB,P4HB,PDIA3,PRDX6,TKT	6
Cellular Development, Cellular Growth and Proliferation, Connective Tissue Development and Function	proliferation of fibroblast cell lines	7.47E-08	ALDOA,CAV1,CLTC,EIF4E3,ERO1L,HINT1,HMGA1,HSPA1A/HSPA1B,NPM1,S100A6,SLC12A6,SRSF1,STMN1,TFG,TFRC,THBS1,YAP1	17
Neurological Disease, Psychological Disorders	disorder of basal ganglia	7.68E-08	AHCY,CAPNS1,CAV1,CD44,EEF1A1,GSTP1,HINT1,HNRNPDL,HSPA1A/HSPA1B,LDHB,NDUFA8,NPM1,PDLIM1,PFN1,PGAM2,PRDX6,PSMB8,PTBP2,RPL3,RTN4,STMN1,SUB1,TPD52L2,VIM	24
Neurological Disease, Skeletal and Muscular Disorders	neuromuscular disease	2.05E-07	AHCY,CAPNS1,CD44,DNAJB11,EEF1A1,GSTP1,HINT1,HNRNPDL,HSPA1A/HSPA1B,LDHB,NDUFA8,NPM1,PDIA3,PDLIM1,PFN1,PGAM2,PRDX6,PSMB8,RPL3,RTN4,SRSF1,STMN1,SUB1,TPD52L2,VIM	25
Cellular Movement	invasion of cells	6.55E-07	AHCY,AHSG,CAPNS1,CAV1,CD44,FABP5,FH,GALNT2,HMGA1,HSP90AB1,HSP90B1,KRT8,LASP1,LGALS3,NCOA3,NPM1,S100A6,SLC12A6,STMN1,THBS1,VIM,YAP1,YWHAQ	23
Immunological Disease	allergy	1.21E-06	AHCY,AHNAK,ALDOA,CD44,EEF1A1,FABP5,HN1,HNRNPAB,P4HB,PDIA3,PRDX6,TFRC,TKT,VCL,ZYX	15
Neurological Disease	Movement Disorders	2.90E-06	AHCY,CAPNS1,CAV1,CD44,EEF1A1,EEF2,FZD4,GSTP1,HINT1,HNRNPDL,HSPA1A/HSPA1B,LDHB,NDUFA8,NPM1,PDLIM1,PFN1,PGAM2,PRDX6,PSMB8,PTBP2,RP	27

			L3,RTN4,scopolamine,STMN1,SUB1,TPD52L2,VIM	
Inflammatory Response	inflammation of organ	2.97E-06	ACTN4,AHCY,AHNAK,ALDOA,ANXA7,CAV1,CD44,EEF1A1,FABP5,GSTP1,HN1,HNRNPAB,HSP90B1,IL27,KRT8,LGALS3,P4HB,PDIA3,PDLIM1,PRDX6,THBS1,TKT,TKTL1,VCL,VIM,YBX1,ZYX	27
Cell Death and Survival	apoptosis	3.00E-06	ACTN4,ALDOA,ANXA7,BAG3,CAPNS1,CAV1,CD44,DNAJC3,DPF1,EEF1A1,EIF4B,GSTP1,HINT1,HMGA1,HNRNPA1,HNRNPK,HSP90AB1,HSP90B1,HSPA1A/HSPA1B,HSPD1,HSPE1,IL27,KRT8,LGALS3,NCOA3,NPM1,P4HB,PDIA3,PRDX3,PRDX4,PRDX6,PSMB8,RTN4,S100A6,SRSF1,STMN1,SUB1,TCOF1,TFRC,THBS1,TMEM109,VCL,VIM,YAP1,YBX1,YWHAE,YWHAQ	47
Cellular Movement	cell movement	5.02E-06	ACTN4,AHCY,AHSG,ALDOA,BAG3,CAPNS1,CAV1,CD44,FABP5,FH,FZD4,GALNT2,GDI1,HNRNPK,HSP90AB1,HSP90B1,HSPA1A/HSPA1B,HSPD1,KRT8,LASP1,LGALS3,MARCKSL1,NCOA3,NPM1,PFN1,RTN4,S100A6,SARS,SF1,SLC12A6,SRSF1,STMN1,THBS1,VCL,VIM,YAP1,YBX1,YWHAE,ZYX	39
Cellular Movement	migration of cells	7.11E-06	ACTN4,AHCY,AHSG,ALDOA,BAG3,CAPNS1,CAV1,CD44,FABP5,FH,FZD4,GALNT2,HNRNPK,HSP90AB1,HSP90B1,HSPA1A/HSPA1B,HSPD1,KRT8,LASP1,LGALS3,MARCKSL1,NCOA3,NPM1,PFN1,RTN4,S100A6,SARS,SF1,STMN1,THBS1,VCL,VIM,YAP1,YBX1,YWHAE,ZYX	36
Neurological Disease	progressive motor neuropathy	7.35E-06	DNAJB11,EEF1A1,GSTP1,HN1,HNRNPA1,HNRNPDL,LDHB,PDIA3,PFN1,PGAM2,RPL3,RTN4,S100A6,STMN1,TFRC,VIM	16
Inflammatory Disease	chronic inflammatory disorder	8.33E-06	ACTN4,AHSG,ALDOA,COTL1,EEF2,GALNT2,HNRNPA1,HSP90B1,HSPA1A/HSPA1B,HSPD1,KHSRP,KRT8,LDHB,P4HB,PDIA3,PDLIM1,PSMB8,SRP14,TFG,TFRC,THBS1,UQCRC2,VIM	23
Cancer	breast or ovarian cancer	9.29E-06	ALDOA,CAV1,CD44,EEF1A1,EIF4B,FABP5,GSTP1,HMGA1,HN1,HNRNPA1,HSP90AB1,HSP90B1,HSPD1,KHSRP,KRT8,LASP1,LGALS3,NCOA3,P4HB,PCDH1,PDIA3,PFN1,S100A6,SLC12A6,THBS1,TPD52L2,VBP1,VCL,VIM,VPS13D,YAP1,YWHAQ,ZYX	33
Cancer	growth of tumor	1.07E-05	ACTN4,AHCY,CAV1,CD44,EEF1A1,EIF4B,GALNT2,HMGA1,HSPA1A/HSPA1B,HSPD1,IL27,LGALS3,NPM1,S100A6,SRSF1,SRSF3,STMN1,TFG,THBS1,YBX1	20
Post-Translational Modification, Protein Folding	folding of protein	1.81E-05	ERO1L,HSP90AB1,HSPA1A/HSPA1B,HSPD1,P4HB,PDIA6	6
Cellular Movement	cell movement of	1.88E-05	ACTN4,CAPNS1,CAV1,CD44,FH,GALNT2,GDI1,HNRNPK,KRT8,LASP1,LGALS3,NCOA3,SLC12A6,STMN1,THBS1,VCL,VIM,YAP1,YBX1,ZYX	20

	tumor cell lines			
Protein Synthesis	translation	2.12E-05	EEF1A1,EEF2,EIF4B,EIF4H,HNRNPK,HSPA1A/HSPA1B,MRPL12,SARS,SNRNP70,YBX1	10
Connective Tissue Disorders, Immunological Disease, Inflammatory Disease, Skeletal and Muscular Disorders	rheumatoid arthritis	2.57E-05	AHSG,ALDOA,EEF2,GALNT2,HNRNPA1,HSP90B1,HSPA1A/HSPA1B,HSPD1,KHSRP,LDHB,PDIA3,PSMB8,SRP14,TFG,TFRC,THBS1,UQCRC2,VIM	18
Neurological Disease	dyskinesia	3.44E-05	AHCY,CAPNS1,CAV1,CD44,HINT1,HNRNPDL,HSPA1A/HSPA1B,LDHB,NDUFA8,NPM1,PDLIM1,PFN1,PGAM2,PRDX6,PSMB8,scopolamine,SUB1,TPD52L2	18
RNA Post-Transcriptional Modification	processing of RNA	3.94E-05	HNRNPA1,HNRNPK,KHSRP,NPM1,RPS28,SARS,SNRNP70,SRSF1,SRSF3,SRSF9	10
Inflammatory Disease, Inflammatory Response, Organismal Injury and Abnormalities, Renal and Urological Disease	diffuse proliferative lupus nephritis	4.20E-05	ACTN4,PDLIM1	2
Connective Tissue Disorders, Metabolic Disease	adiposity	4.22E-05	CAV1,DNAJC3,FABP5,LGALS3,NCOA3,PRKAG3,TKT	7
Tissue Development	growth of connective tissue	4.55E-05	CAPNS1,CAV1,CD44,CLTC,GSTP1,HMGA1,HSPA1A/HSPA1B,LGALS3,NPM1,PGAM2,PRDX4,SLC12A6,STMN1,TFG,VCL,YAP1,YBX1	17
Cellular Movement	invasion of prostate cancer cell lines	4.62E-05	CAV1,FABP5,LGALS3,NCOA3,VIM,YWHAQ	6
Cell Death and Survival	cell death	5.14E-05	ACTN4,ALDOA,ANXA7,BAG3,CAPNS1,CAV1,CD44,DNAJC3,DPF1,EEF1A1,EIF4B,EN2,EPG5,GSTP1,HINT1,HMGA1,HNRNPA1,HNRNPK,HSP90AB1,HSP90B1,HSPA1A/HSPA1B,HSPD1,HSPE1,IL27,KRT8,LGALS3,MANF,NCOA3,NPM1,P4HB,PDIA3,PRDX3,PRDX4,PRDX6,PSMB8,RTN4,S100A6,scopolamine,SLC12A6,SRSF1,STMN1,SUB1,TCOF1,TFRC,THBS1,TMEM109,VCL,VIM,YAP1,YBX1,YWHAE,YWHAQ,ZY	53

			X	
Cellular Movement	invasion of tumor cell lines	5.17E-05	CAPNS1,CAV1,CD44,FABP5,FH,GALNT2,HMGA1,KRT8,LGALS3,NCOA3,S100A6,SLC12A6,STMN1,VIM,YAP1,YWHAQ	16
Cellular Growth and Proliferation, Tissue Development	proliferation of connective tissue cells	5.25E-05	CAPNS1,CAV1,CD44,CLTC,GSTP1,HSPA1A/HSPA1B,LGALS3,NPM1,PGAM2,PRDX4,SLC12A6,STMN1,TFG,VCL,YAP1,YBX1	16
Hereditary Disorder, Neurological Disease, Psychological Disorders, Skeletal and Muscular Disorders	Huntington's Disease	5.33E-05	AHCY,CAPNS1,CD44,HINT1,HNRNPDL,HSPA1A/HSPA1B,LDHB,NDUFA8,NPM1,PDLIM1,PFN1,PGAM2,PRDX6,PSMB8,SUB1,TPD52L2	16
Neurological Disease	amyotrophic lateral sclerosis	5.40E-05	EEF1A1,HN1,HNRNPA1,PFN1,PGAM2,RTN4,S100A6,TFRC,VIM	9
Neurological Disease	chorea	5.57E-05	AHCY,CAPNS1,CD44,HINT1,HNRNPDL,HSPA1A/HSPA1B,LDHB,NDUFA8,NPM1,PDLIM1,PFN1,PGAM2,PRDX6,PSMB8,scopolamine,SUB1,TPD52L2	17
Cellular Movement	invasion of fibroblast cell lines	5.61E-05	CAV1,CD44,FABP5,LASP1,NPM1	5
Protein Synthesis	expression of protein	5.61E-05	CAV1,EEF1A1,EIF4B,EIF4H,HNRNPK,HSPA1A/HSPA1B,MRPL12,SARS,SNRNP70,THBS1,YBX1	11
Cancer, Organismal Injury and Abnormalities, Reproductive System Disease	breast cancer	5.93E-05	ALDOA,CAV1,CD44,EEF1A1,EIF4B,FABP5,GSTP1,HMGA1,HNRNPA1,HSP90AB1,HSP90B1,HSPD1,KHSRP,KRT8,LASP1,LGALS3,NCOA3,P4HB,PCDH1,PFN1,S100A6,THBS1,VBP1,VCL,VPS13D,YAP1,YWHAQ,ZYX	28
Cellular Function and Maintenance	engulfment of tumor cell lines	6.49E-05	CAV1,CD44,CLTC,PFN1,RPS21,SRSF3,VIM	7
Cell Death and Survival	apoptosis of tumor cell lines	6.51E-05	BAG3,CAPNS1,CAV1,CD44,EIF4B,GSTP1,HINT1,HMGA1,HNRNPA1,HNRNPK,HSP90AB1,HSPA1A/HSPA1B,HSPD1,LGALS3,NCOA3,NPM1,P4HB,RTN4,S100A6,SRSF1,STMN1,TFRC,THBS1,YBX1,YWHAE	25
Cell Death and Survival	necrosis	6.85E-05	ALDOA,BAG3,CAPNS1,CAV1,CD44,EEF1A1,EIF4B,EN2,EPG5,GSTP1,HINT1,HMGA1,HNRNPA1,HNRNPK,HSP90AB1,HSP90B1,HSPA1A/HSPA1B,HSPD1,HSPE1,IL2	44

			7,KRT8,LGALS3,MANF,NCOA3,NPM1,P4HB,PDIA3,PRDX3,PRDX6,PSMB8,RTN4,S100A6,scopolamine,SRSF1,STMN1,TFRC,THBS1,TMEM109,VIM,YAP1,YBX1,YWHAE,YWHAQ,ZYX	
Cell Death and Survival	anoikis of breast cancer cell lines	7.33E-05	CAV1,LGALS3,NCOA3	3
RNA Post-Transcriptional Modification	selection of splice site	7.33E-05	PTBP2,SRSF1,SRSF9	3
Cancer, Cellular Development, Cellular Growth and Proliferation, Tumor Morphology	proliferation of tumor cells	7.41E-05	ACTN4,AHCY,CAV1,CD44,EEF1A1,HSPA1A/HSPA1B,LGALS3,NPM1,S100A6,SRSF1,SRSF3,STMN1,TFG,THBS1	14
Cell Death and Survival	cell death of breast cancer cell lines	7.49E-05	BAG3,CAV1,CD44,HINT1,HSPA1A/HSPA1B,HSPD1,LGALS3,NCOA3,STMN1,TFRC,THBS1	11
Cardiovascular System Development and Function, Cell-To-Cell Signaling and Interaction, Tissue Development	adhesion of endothelial cell lines	8.57E-05	CAV1,CD44,LGALS3,THBS1	4
Cancer	osteosarcoma	8.76E-05	CD44,HINT1,HSP90AB1,HSP90B1,NCOA3,THBS1	6
Cancer, Organismal Injury and Abnormalities, Renal and Urological Disease	transitional cell bladder cancer	9.29E-05	GSTP1,HSP90AB1,HSP90B1	3
Protein Synthesis	synthesis of protein	9.74E-05	CAV1,EEF1A1,EEF2,EIF4B,EIF4H,HNRNPK,HSPA1A/HSPA1B,MRPL12,NPM1,SARS,SNRNP70,THBS1,YBX1	13
Infectious Disease	Viral Infection	9.75E-05	CAV1,CD44,CLTC,EEF1A1,GANAB,HMGA1,HNRNPDL,HNRNPK,HSP90AB1,HSP90B1,HSPD1,IL27,KHSRP,KRT8,LGALS3,NCOA3,NPM1,PDIA3,PDIA6,POLR2K,RPL3,RUSC2,SNRNP70,SRSF1,SUB1,TFRC,TIMM8A,TKT,YBX1,ZYX	30
Protein Synthesis	translation of protein	1.09E-04	EEF1A1,EIF4B,EIF4H,HNRNPK,HSPA1A/HSPA1B,MRPL12,SARS,SNRNP70,YBX1	9
Protein Trafficking	targeting of	1.23E-04	TIMM10,TIMM8A,TOMM22,TOMM40,YWHAE,YWHAQ	6

	protein			
Cellular Development, Cellular Growth and Proliferation	proliferation of tumor cell lines	1.23E-04	ACTN4,AHSG,ALDOA,ANXA7,CAV1,CD44,EEF1A1,EIF4B,FABP5,GALNT2,HMGA1,HNRNPA1,HNRNPK,KRT8,LGALS3,NCOA3,NPM1,PDIA3,PFN1,S100A6,SRSF1,SRSF3,STMN1,TFRC,THBS1,YAP1,YBX1,YWHAQ	28
Cancer, Respiratory Disease	advanced stage primary laryngeal cancer	1.25E-04	HSP90AB1,HSP90B1	2
Cellular Assembly and Organization	alignment of filaments	1.25E-04	CAV1,MARCKSL1	2
Cancer, Immunological Disease, Organismal Injury and Abnormalities, Reproductive System Disease	node positive cervical cancer	1.25E-04	HSP90AB1,HSP90B1	2
Energy Production, Nucleic Acid Metabolism, Small Molecule Biochemistry	recovery of ATP	1.25E-04	HSPD1,HSPE1	2
Cancer, Endocrine System Disorders, Organismal Injury and Abnormalities	benign thyroid nodule	1.42E-04	GSTP1,HSP90AB1,PRDX6	3
Cellular Function and Maintenance	engulfment of cells	1.48E-04	ACTN4,AHSG,CAV1,CD44,CLTC,LGALS3,NPM1,PFN1,RPS21,SRSF3,THBS1,VIM	12
Connective Tissue Disorders, Inflammatory Disease, Skeletal and Muscular Disorders	Rheumatic Disease	1.50E-04	AHSG,ALDOA,CD44,EEF2,GALNT2,HNRNPA1,HSP90B1,HSPA1A/HSPA1B,HSPD1,HSPE1,KHSRP,LDHB,PDIA3,PSMB8,SRP14,SRSF1,SRSF3,TFG,TFRC,THBS1,UQCRC2,VIM	22
Cell Death and Survival	cell death of muscle cells	1.61E-04	ALDOA,CAV1,EEF1A1,EPG5,HSPD1,HSPE1,MANF,PRDX3,S100A6,YAP1,ZYX	11
Cellular Function and Maintenance,	phagocytosis	1.68E-04	AHSG,CAV1,CD44,CLTC,EPG5,LGALS3,NPM1,PFN1,THBS1,VIM	10

Inflammatory Response				
Cardiovascular System Development and Function, Organ Morphology	dilation of left ventricle	1.73E-04	CAPNS1,CAV1,MARCKSL1,PRDX3	4
Cellular Development, Hematological System Development and Function, Hematopoiesis, Tissue Development	myelopoiesis of bone marrow	2.04E-04	CD44,IL27,NPM1	3
Inflammatory Response	inflammation of body region	2.05E-04	ACTN4,ALDOA,ANXA7,CAV1,CD44,GSTP1,HNRNPAB,HSP90B1,IL27,KRT8,LGALS3,P4HB,PDIA3,PDLIM1,PRDX6,THBS1,TKT,TKTL1,VIM,YBX1	20
Cellular Development, Cellular Growth and Proliferation	proliferation of breast cancer cell lines	2.06E-04	CAV1,CD44,EEF1A1,HMGA1,HNRNPK,KRT8,LGALS3,NCOA3,PDIA3,PFN1,YBX1,YWHAQ	12
Cardiovascular System Development and Function, Organ Morphology, Organismal Development, Tissue Morphology	morphology of heart tissue	2.33E-04	CAV1,HMGA1,HSP90B1,MARCKSL1,THBS1,VCL,YWHAE	7
Cancer	follicular adenoma	2.41E-04	GSTP1,HSP90AB1,PRDX6	3
Gastrointestinal Disease	abnormal absorption of mineral in intestine	2.50E-04	KRT8,TFRC	2
Molecular Transport	transport of	2.51E-04	CAV1,CD44,CSN2,FABP5,FH,FZD4,HMGA1,Hnrnpa3,KRT8,LASP1,MARCKS	28

	molecule		L1,NPM1,P4HB,PDIA3,PDIA4,PRDX6,PRKAG3,RTN4,S100A6,SCAMP3,SEC61B,SLC12A6,SLC4A9,SRSF3,TFRC,THBS1,TOMM40,YWHAE	
Immunological Disease	systemic autoimmune syndrome	2.65E-04	AHSG,ALDOA,CD44,EEF2,GALNT2,HNRNPA1,HSP90B1,HSPA1A/HSPA1B,HSPD1,KHSRP,LDHB,PDIA3,PSMB8,S100A6,SRP14,SRSF1,TFG,TFRC,THBS1,UQCRC2,VIM	21
Cellular Development, Cellular Growth and Proliferation	proliferation of prostate cancer cell lines	2.88E-04	ANXA7,CAV1,CD44,FABP5,HNRNPA1,LGALS3,NCOA3,YBX1,YWHAQ	9
Gastrointestinal Disease	abnormal function of digestive system	3.21E-04	CAV1,CD44,KRT8,SLC4A9,TFRC	5
Cancer, Cell-To-Cell Signaling and Interaction	binding of cancer cells	3.28E-04	CD44,PDIA3,THBS1	3
Cardiovascular System Development and Function	angiogenesis	3.30E-04	BAG3,CAV1,CD44,FZD4,HMGA1,HSP90B1,HSPD1,LGALS3,MARCKSL1,NCOA3,RTN4,SARS,THBS1,VCL,VIM,YAP1,YWHAE	17
Cell Morphology, Connective Tissue Development and Function	cell spreading of fibroblast cell lines	3.37E-04	RTN4,THBS1,VCL,VIM	4
Cellular Movement	migration of tumor cells	3.40E-04	ACTN4,AHCY,CAV1,CD44,LGALS3,S100A6,THBS1,VIM	8
Cardiovascular System Development and Function, Organismal Development, Tissue Morphology	morphology of cardiovascular tissue	3.48E-04	CAV1,CD44,HMGA1,HSP90B1,MARCKSL1,THBS1,VCL,YWHAE	8
Cell-To-Cell Signaling and Interaction,	phagocytosis of cells	3.52E-04	AHSG,CAV1,CD44,CLTC,LGALS3,NPM1,PFN1,THBS1,VIM	9

Cellular Function and Maintenance, Inflammatory Response				
Cell Cycle	senescence of cells	3.75E-04	AHCY,CAV1,HSPA1A/HSPA1B,NPM1,SRSF1,SRSF3,YAP1,YBX1	8
RNA Post-Transcriptional Modification	splicing of RNA	3.76E-04	KHSRP,NPM1,SNRNP70,SRSF1,SRSF3,SRSF9	6
Cellular Movement	migration of connective tissue cells	3.94E-04	CAPNS1,CAV1,CD44,STMN1,THBS1,VCL,ZYX	7
Cellular Movement, Connective Tissue Development and Function	cell movement of fibroblasts	4.06E-04	CAPNS1,CAV1,CD44,STMN1,THBS1,VCL,ZYX	7
Carbohydrate Metabolism, Drug Metabolism, Small Molecule Biochemistry	modification of hyaluronic acid	4.14E-04	CD44,THBS1	2
Cell Death and Survival	apoptosis of breast cancer cell lines	4.26E-04	BAG3,CAV1,CD44,HINT1,HSPD1,LGALS3,NCOA3,TFRC,THBS1	9
Cellular Movement	migration of tumor cell lines	4.41E-04	ACTN4,CAPNS1,CAV1,CD44,FH,GALNT2,HNRNPK,KRT8,LASP1,LGALS3,NCOA3,STMN1,THBS1,VIM,ZYX	15
Cancer, Endocrine System Disorders	small-cell carcinoma	4.52E-04	HSP90AB1,HSP90B1,HSPD1,THBS1,YWHAE	5
Cell Death and Survival	apoptosis of colon cancer cell lines	4.56E-04	BAG3,CD44,GSTP1,HINT1,HMGA1,HSPD1,LGALS3,YWHAE	8

Cellular Compromise, Cellular Function and Maintenance	endoplasmic reticulum stress response	4.66E-04	ERO1L,HSP90AB1,HSP90B1,HSPA1A/HSPA1B,HSPD1,HSPE1	6
Gene Expression	transactivation	5.02E-04	ACTN4,CAV1,CD44,FABP5,HMGA1,HNRNPA1,HNRNPK,MARCKSL1,NCOA3,NPM1,PDIA3,POLR2K,SF1,SUB1	14
Inflammatory Response	immune response of cells	5.17E-04	AHSG,CAV1,CD44,CLTC,LGALS3,NPM1,PFN1,PSMB8,THBS1,VIM	10
Cellular Movement, Connective Tissue Development and Function	migration of fibroblasts	5.73E-04	CAPNS1,CAV1,STMN1,THBS1,VCL,ZYX	6
Cancer	metastasis	5.82E-04	AHSG,ANXA7,CAV1,CD44,EEF1A1,FABP5,HMGA1,HSP90AB1,HSP90B1,KRT7,KRT8,LGALS3,THBS1,VIM,YAP1,YWHAE	16
Cellular Compromise	stress response of tumor cell lines	5.90E-04	ERO1L,HNRNPA1,HSP90B1,PRDX6	4
Cellular Movement	migration of cancer cells	6.01E-04	AHCY,CAV1,CD44,LGALS3,S100A6,THBS1,VIM	7
Cancer, Organismal Injury and Abnormalities, Reproductive System Disease	advanced uterine cancer	6.19E-04	HSP90AB1,HSP90B1	2
Cancer, Endocrine System Disorders, Organismal Injury and Abnormalities, Reproductive System Disease	metastatic ovarian tumor	6.19E-04	HSP90AB1,HSP90B1	2

Cell-To-Cell Signaling and Interaction	binding of breast cancer cell lines	6.26E-04	CD44,LGALS3,THBS1	3
Inflammatory Response, Respiratory Disease	inflammation of lung	6.63E-04	ALDOA,CAV1,CD44,HNRNPAB,LGALS3,P4HB,PDIA3,PRDX6,THBS1,TKT	10
Connective Tissue Disorders, Inflammatory Disease, Skeletal and Muscular Disorders	arthritis	6.95E-04	AHSG,ALDOA,CD44,EEF2,GALNT2,HNRNPA1,HSP90B1,HSPA1A/HSPA1B,HSPD1,KHSRP,LDHB,PDIA3,PSMB8,SRP14,TFG,TFRC,THBS1,UQCRC2,VIM	19
RNA Post-Transcriptional Modification	processing of mRNA	6.97E-04	HNRNPA1,HNRNPK,NPM1,SNRNP70,SRSF1,SRSF9	6
Cell Death and Survival	cell survival	7.20E-04	ATP5H,BAG3,CAV1,CD44,EEF2,EN2,FH,GSTP1,HINT1,HMGA1,HSP90AB1,HSP90B1,HSPA1A/HSPA1B,HSPD1,IL27,LGALS3,P4HB,PDIA3,PRDX6,S100A6,STMN1,THBS1,VCL,VIM,YBX1	25
Cellular Movement	cell movement of lung cancer cell lines	7.34E-04	CAV1,STMN1,VIM,YBX1,ZYX	5
Cancer, Respiratory Disease	laryngeal squamous cell carcinoma	7.82E-04	CD44,HSP90AB1,HSP90B1	3
Cellular Movement	migration of bone cancer cell lines	7.82E-04	ACTN4,CAV1,STMN1	3
Inflammatory Response	inflammation of body	7.89E-04	ALDOA,ANXA7,CAV1,CD44,GSTP1,HNRNPAB,KRT8,LGALS3,P4HB,PDIA3,PRDX6,THBS1,TKT	13

	cavity			
Gene Expression	transactivation of RNA	8.31E-04	ACTN4,CAV1,CD44,FABP5,HMGA1,HNRNPA1,HNRNPK,NCOA3,NPM1,PDIA3,POLR2K,SF1,SUB1	13
Cellular Assembly and Organization, Cellular Function and Maintenance	organization of membrane rafts	8.63E-04	CAV1,CD44	2
Inflammatory Response	inflammation of pancreas	8.68E-04	ANXA7,KRT8,THBS1	3
Cell-To-Cell Signaling and Interaction	response of connective tissue cells	8.68E-04	LGALS3,NCOA3,THBS1	3
Neurological Disease, Psychological Disorders, Skeletal and Muscular Disorders	Parkinson's disease	8.76E-04	EEF1A1,GSTP1,HNRNPDL,LDHB,RPL3,RTN4,STMN1,VIM	8
Cell Death and Survival	cell viability of neuroblastoma cell lines	9.02E-04	BAG3,HSP90AB1,P4HB,S100A6	4
Dermatological Diseases and Conditions, Inflammatory Disease, Inflammatory Response	Dermatitis	9.37E-04	AHCY,AHNAK,CD44,EEF1A1,FABP5,GSTP1,HN1,IL27,THBS1,VCL,ZYX	11
Dermatological Diseases and Conditions	psoriasis	9.66E-04	CAV1,EEF1A1,FABP5,GSTP1,HSPE1,LGALS3,MANF,P4HB,SEC61B,SRSF9,SUB1,YWHAE,YWHAQ	13
Protein Synthesis	metabolism of protein	9.96E-04	BAG3,CAPNS1,CAV1,EEF1A1,EEF2,EIF4B,EIF4H,HNRNPK,HSP90B1,HSPA1A/HSPA1B,HSPD1,MRPL12,NPM1,PDIA3,SARS,SNRNP70,THBS1,YBX1	18

Table S8. Disease and function analysis of mapped differentially expressed proteins between DU145 and DU145RR cell lines

Categories	Diseases or Functions Annotation	p-Value	Molecules	Molecules numbers
Cellular Growth and Proliferation	proliferation of cells	9.69E-13	AGR2,AHNAK,AHSG,AK2,ALB,ALDOA,ANXA2,ANXA6,APOA1,ATIC,ATP5A1,BASP1,C1QBP,CALR,CAPNS1, CAPRIN1,CCT2,CDC37,CFL1,CIRBP,CLIC1,CTSL,CYBA,DBI,DLD,DYNC1H1, EEF1B2,EEF1D,EIF4B,ELAVL1, ENO1,EPB41L3,EZR,FABP5,FGF6,FLNA,GNPNAT1,GSN,GSS,H2AFX,HINT1,HNRNPA2B1,HNRNPC,HNRNPF, HNRNPM,HNRNPR,HSP90AB1,HSPD1,IMPDH2,LDHA,LGALS3,LMNA,MAPRE1,MCM3,MTDH,MYH9,NAP1L1, NCL,NDRG1,NF2,NQO1,NUDC,PDIA3,PGK1,PLIN3,PPIB,PRKCSH,PTBP1,RPS14,RPS9,SDHB,SERPINA1,SFN, SHMT2,TAGLN2,TF,TFAM,TGM2,TMPO,TPD52,TXLNA,TXNDC5,VIM,YBX1, YWHAG	85
Protein Synthesis	metabolism of protein	1.38E-11	APOA1,APOA2,CALR,CANX,CAPNS1,CAPRIN1,CDC37,CIRBP,CNDP2,CTSL, DHX9,DLD,EEF1B2,EIF4B,EIF4H,ELAVL1,FLNA,GSN,HSPD1,LAMP1,MYH9, NCL,NF2,PABPC1,PDIA3,PPIB,PSMB3,PTBP1,RPS14,RPS3,RPS7,RPS9,SERPINA1,SYNCRIP,TGM2,TUFM,YBX1	37
Protein Synthesis	translation	5.47E-11	CALR,CAPRIN1,CIRBP,DHX9,EEF1B2,EIF4B,EIF4H,ELAVL1,NCL,PABPC1,PTBP1,RPS14,RPS3,RPS7,RPS9,SYNCRIP,TUFM,YBX1	18
Cell Death and Survival	cell death	2.04E-10	AGR2,ALB,ALDOA,ANXA2,ANXA4,ANXA5,APOA1,ATP5A1,BASP1,C1QBP,CALR,CANX,CAPNS1,CAPRIN1,CCT2,CDC37,CFL1,CIRBP,CTSL,CYBA,DHX9, DUT,DYNC1H1,EEF1D,EHD1,EIF4B,ELAVL1,ENO1,EZR,FGF6,FLNA,GNPNAT1,GSN,GSS,H2AFX,HEBP2,HINT1,HNRNPC,HSD17B10,HSP90AB1,HSPD1,IMPDH2,LAMP1,LDHA,LGALS3,LMNA,MSN,MTDH,MYH9,NCL,NDRG1,NF2,NQO1,PAFAH1B2,PDIA3,PLIN3,PPIB,PRDX6,RPS3,SDHA,SDHB,SERPINA1,SF3A1,SFN,SH3BGRL3,TAGLN2,TF,TFAM,TGM2,TPD52,TUFM,TXNDC5,VIM,YBX1,YWHAE,YWHAG	76
Protein Synthesis	translation of protein	3.62E-10	CALR,CAPRIN1,CIRBP,DHX9,EEF1B2,EIF4B,EIF4H,ELAVL1,NCL,PABPC1,PTBP1,RPS14,RPS3,RPS7,RPS9,SYNCRIP,YBX1	17
Cancer, Respiratory Disease	respiratory system tumor	9.55E-10	AGR2,AHNAK,AHNAK2,AHSG,AK2,ALDOA,ANXA2,APOA1,ATIC,CAPRIN1, CYBA,DHX9,EEF1B2,ENO1,EZR,FABP5,GSN,HSP90AB1,HSPD1,IMPDH2,ITIH	35

			2,LDHA,MSN,MYH9,NCL,NDRG1,NF2,NQO1,PABPC1,PSMB4,SFN,SHMT2,TUBB4A,VIM,YWHAE	
Cell Death and Survival	apoptosis	6.12E-09	ALB,ALDOA,ANXA2,ANXA4,ANXA5,APOA1,BASP1,C1QBP,CALR,CANX,CAPNS1,CAPRIN1,CCT2,CDC37,CFL1,CIRBP,CTSL,CYBA,DHX9,DUT,DYNC1H1,EEF1D,EHD1,EIF4B,ELAVL1,ENO1,EZR,FLNA,GNPNAT1,GSN,H2AFX,HEBP2,HINT1,HNRNPC,HSD17B10,HSP90AB1,HSPD1,LDHA,LGALS3,LMNA,MSN,MTDH,NCL,NDRG1,NF2,NQO1,PAFAH1B2,PDIA3,PRDX6,RPS3,SERPINA1,SFN,SH3BGRL3,TAGLN2,TF,TFAM,TGM2,TPD52,TXNDC5,VIM,YBX1,YWHAE	62
Cancer, Respiratory Disease	lung tumor	6.61E-09	AGR2,AHNAK,AHNAK2,AHSG,AK2,ALDOA,ANXA2,APOA1,ATIC,CAPRIN1,CYBA,DHX9,EEF1B2,ENO1,EZR,GSN,HSP90AB1,HSPD1,IMPDH2,ITIH2,LDHA,MSN,MYH9,NCL,NDRG1,NF2,NQO1,PABPC1,PSMB4,SHMT2,TUBB4A,VIM,YWHAE	33
Cancer, Respiratory Disease	Lung Cancer and Tumors	6.79E-09	AHNAK,AHNAK2,AHSG,AK2,ALDOA,ANXA2,APOA1,ATIC,CAPRIN1,CYBA,DHX9,EEF1B2,ENO1,EZR,GSN,HSP90AB1,HSPD1,IMPDH2,ITIH2,LDHA,MSN,MYH9,NCL,NF2,NQO1,PABPC1,PSMB4,SHMT2,TFAM,TUBB4A,VIM,YWHAE	32
Hematological Disease, Immunological Disease, Inflammatory Disease, Inflammatory Response, Respiratory Disease	allergic pulmonary eosinophilia	7.60E-09	ALB,ALDOA,ENO1,MYH9,PDIA3,PRDX6,TKT	7
Cancer	malignant neoplasm of thorax	1.15E-08	AHNAK,AHNAK2,AHSG,AK2,ALDOA,ANXA2,APOA1,ATIC,CAPRIN1,CYBA,DHX9,EEF1B2,ENO1,EZR,GSN,H2AFX,HSP90AB1,HSPD1,IMPDH2,ITIH2,LDHA,MSN,MYH9,NCL,NF2,NQO1,PABPC1,PSMB4,SHMT2,TUBB4A,VIM,YWHAE	32
Cancer	breast or ovarian cancer	2.08E-08	AGR2,ALDOA,APOA1,ATIC,ATP5A1,C1QBP,CNDP2,COX4I1,CYBA,DYNC1H1,EIF4B,ENO1,FABP5,FLNA,GSN,H2AFX,HSP90AB1,HSPD1,KRT15,LGALS3,LMNA,MAPRE1,MTDH,MYH9,NCL,NQO1,PAFAH1B2,PDIA3,PGK1,PGM1,PRKCSH,PSMA8,PSMB4,PTBP1,PTRF,RPL6,RPS3,SERPINA1,SHMT2,TAGLN2,TGM2,TPD52,TUBB4A,VIM	44
Cancer, Respiratory Disease	lung cancer	2.08E-08	AHNAK,AHNAK2,AHSG,AK2,ALDOA,ANXA2,APOA1,ATIC,CAPRIN1,CYBA,DHX9,EEF1B2,ENO1,EZR,GSN,HSP90AB1,HSPD1,IMPDH2,ITIH2,LDHA,MSN,MYH9,NCL,NF2,NQO1,PABPC1,PSMB4,SHMT2,TUBB4A,VIM,YWHAE	31
Immunological	allergy	2.60E-08	AHNAK,ALB,ALDOA,ANXA5,CFL1,CTSL,ENO1,FABP5,FLNA,GSN,HNRNPR,	19

Disease			IDI1,KRT15,MSN,MYH9,PDIA3,PRDX6,TGM2,TKT	
Cancer, Respiratory Disease	carcinoma in lung	2.91E-08	AHNAK,AHNAK2,AHSG,AK2,ALDOA,ANXA2,APOA1,ATIC,CAPRIN1,CYBA,DHX9,EEF1B2,ENO1,EZR,GSN,HSP90AB1,HSPD1,IMPDH2,ITIH2,LDHA,MSN,MYH9,NF2,NQO1,PABPC1,TUBB4A,VIM,YWHAE	28
Protein Degradation, Protein Synthesis	stabilization of protein	5.02E-08	APOA1,APOA2,CALR,CANX,CDC37,FLNA,HSPD1,LAMP1,NF2,PPIB	10
Post-Translational Modification	conformational modification of protein	9.91E-08	APOA2,CALR,CANX,ERP29,GSN,HSP90AB1,HSPD1,PFDN2,ST13	9
Cancer, Endocrine System Disorders, Organismal Injury and Abnormalities	benign cold thyroid nodule	1.18E-07	ANXA5,APOA1,CALR,HSP90AB1,PRDX6	5
RNA Post-Transcriptional Modification	splicing of RNA	1.19E-07	AHNAK,C1QBP,DDX39B,HNRNPA2B1,HNRNPF,HNRNPH1,HNRNPH3,HNRNPM,PTBP1,SF3A1,SYNCRIP	11
RNA Post-Transcriptional Modification	processing of RNA	1.44E-07	AHNAK,ALB,C1QBP,DDX39B,HNRNPA2B1,HNRNPF,HNRNPH1,HNRNPH3,HNRNPM,PABPC1,PTBP1,RPS7,SF3A1,SF3B4,SYNCRIP	15
Cancer	Breast Cancer and Tumors	2.22E-07	AGR2,ALDOA,APOA1,ATP5A1,C1QBP,CDC37,CNDP2,COX4I1,CYBA,DYNC1H1,EIF4B,ENO1,FABP5,FLNA,GSN,HSP90AB1,HSPD1,KRT15,LGALS3,LMNA,MAPRE1,MTDH,MYH9,NCL,NQO1,PAFAH1B2,PGK1,PGM1,PRKCSH,PSMB4,PTBP1,PTRF,RPL6,RPS3,SHMT2,TAGLN2,TPD52,TUBB4A	38
Cell Death and Survival	cell death of tumor cell lines	2.34E-07	AGR2,ALB,ANXA2,ATP5A1,CALR,CAPNS1,CCT2,CYBA,DUT,DYNC1H1,EIF4B,ELAVL1,ENO1,EZR,FGF6,GSN,H2AFX,HINT1,HNRNPC,HSP90AB1,HSPD1,LAMP1,LGALS3,LMNA,MSN,MTDH,NCL,NDRG1,NQO1,RPS3,SF3A1,SFN,TAGLN2,TF,TGM2,TPD52,TUFM,YBX1,YWHAE,YWHAG	40
Protein Synthesis	synthesis of protein	2.83E-07	CALR,CAPRIN1,CIRBP,DHX9,EEF1B2,EIF4B,EIF4H,ELAVL1,GSN,NCL,PABPC1,PTBP1,RPS14,RPS3,RPS7,RPS9,SYNCRIP,TUFM,YBX1	19
Cell Death and Survival	necrosis	2.99E-07	AGR2,ALB,ALDOA,ANXA2,APOA1,ATP5A1,CALR,CAPNS1,CAPRIN1,CCT2,CDC37,CYBA,DUT,DYNC1H1,EEF1D,EIF4B,ELAVL1,ENO1,EZR,FGF6,FLNA,GNPNAT1,GSN,H2AFX,HINT1,HNRNPC,HSD17B10,HSP90AB1,HSPD1,LAMP1,LDHA,LGALS3,LMNA,MSN,MTDH,NCL,NDRG1,NF2,NQO1,PDIA3,PRDX6,RPS3,SDHA,SDHB,SERPINA1,SF3A1,SFN,TAGLN2,TF,TFAM,TGM2,TPD52,TUF	57

			M,VIM,YBX1,YWHAE,YWHAG	
Cancer	follicular adenoma	3.08E-07	ANXA5,APOA1,CALR,HSP90AB1,PRDX6	5
Cancer, Respiratory Disease	non-small cell lung cancer	4.05E-07	AHNAK,AHNAK2,AK2,ALDOA,ANXA2,ATIC,CAPRIN1,CYBA,DHX9,EEF1B2,ENO1,EZR,GSN,HSP90AB1,HSPD1,IMPDH2,ITIH2,LDHA,MSN,MYH9,NF2,NQO1,PABPC1,TUBB4A,VIM	25
Cancer, Organismal Injury and Abnormalities, Reproductive System Disease	breast cancer	5.74E-07	AGR2,ALDOA,APOA1,ATP5A1,C1QBP,CNDP2,COX4I1,CYBA,DYNC1H1,EIF4B,ENO1,FABP5,FLNA,GSN,HSP90AB1,HSPD1,KRT15,LGALS3,LMNA,MAPRE1,MTDH,MYH9,NCL,NQO1,PAFAH1B2,PGK1,PGM1,PRKCSH,PSMB4,PTBP1,PTRF,RPL6,RPS3,SHMT2,TAGLN2,TPD52,TUBB4A	37
Infectious Disease	Viral Infection	6.47E-07	ALB,ANXA2,ANXA5,ANXA6,CCT2,CHMP4B,CTSL,DHX9,FLNA,HNRNPC,HNRNPF,HNRNPH1,HNRNPM,HSP90AB1,HSPD1,IMPDH2,LGALS3,LMNA,NCL,NF2,PDIA3,PGM1,PLIN3,PPIB,PSMA7,RPL12,RPL3,RPS10,RPS14,RPS23,SERPINA1,SERPINB6,SF3A1,SFN,ST13,TAGLN2,TKT,TMPO,TUBB4A,YBX1	40
Cancer, Respiratory Disease	non-squamous non-small cell lung cancer	6.82E-07	AHNAK,ALDOA,ATIC,CAPRIN1,CYBA,DHX9,EEF1B2,ENO1,EZR,HSP90AB1,IMPDH2,ITIH2,LDHA,MSN,MYH9,NF2,NQO1,TUBB4A	18
Inflammatory Response	inflammation of organ	8.14E-07	AGR2,AHNAK,ALB,ALDOA,ANXA5,APOA1,APOA2,CALR,CFL1,CTSL,ELAVL1,ENO1,FABP5,FLNA,GSN,HNRNPR,IDI1,IMPDH2,KRT15,LGALS3,MSN,MYH9,NQO1,PDIA3,PRDX6,SFN,TF,TGM2,TKT,TUBB4A,VIM,YBX1	32
Post-Translational Modification, Protein Folding	folding of protein	8.68E-07	APOA2,CALR,CANX,ERP29,HSP90AB1,HSPD1,PFDN2,ST13	8
Cancer, Organismal Injury and Abnormalities, Reproductive System Disease	mammary tumor	1.31E-06	AGR2,ALDOA,APOA1,ATP5A1,C1QBP,CNDP2,COX4I1,CYBA,DYNC1H1,EIF4B,ENO1,FABP5,FLNA,GSN,HSP90AB1,HSPD1,KRT15,LGALS3,LMNA,MAPRE1,MTDH,MYH9,NCL,NF2,NQO1,PAFAH1B2,PGK1,PGM1,PRKCSH,PSMB4,PTBP1,PTRF,RPL6,RPS3,SHMT2,TAGLN2,TPD52,TUBB4A	38
Nucleic Acid Metabolism, Small Molecule Biochemistry	metabolism of nucleoside triphosphate	2.34E-06	AK2,ALDOA,ATP5A1,ATP5D,ATP5H,ATP5O,DCTPP1,DDX39B,DHX9,HSPD1,MYH9,RHOBTB3,TFAM	13
Cancer, Respiratory	non small cell	2.47E-06	AHNAK,ALDOA,ATIC,CAPRIN1,CYBA,DHX9,EEF1B2,ENO1,EZR,IMPDH2,ITI	17

Disease	lung adenocarcinoma		H2,LDHA,MSN,MYH9,NF2,NQO1,TUBB4A	
RNA Post-Transcriptional Modification	processing of mRNA	3.57E-06	ALB,C1QBP,DDX39B,HNRNPA2B1,HNRNPH3,HNRNPM,PABPC1,PTBP1,SF3A1,SF3B4	10
Gene Expression, Protein Synthesis	translation of mRNA	3.73E-06	CALR,CAPRIN1,CIRBP,EEF1B2,EIF4B,EIF4H,ELAVL1,RPS14,RPS9,SYNCRIP	10
Protein Degradation, Protein Synthesis	catabolism of protein	4.04E-06	APOA1,APOA2,CALR,CANX,CAPNS1,CDC37,CNDP2,CTSL,DLD,FLNA,GSN,HSPD1,LAMP1,MYH9,NF2,PDIA3,PPIB,PSMB3,SERPINA1,TGM2	20
Protein Synthesis	polymerization of protein	4.71E-06	AHNAK,ANXA2,ANXA5,ANXA6,CHMP4B,DCTPP1,EHD1,GSN,HSD17B10,IMPDH2,PRKCSH,SHMT2,ST13,TGM2	14
Cancer, Endocrine System Disorders	thyroid adenoma	4.90E-06	ANXA5,APOA1,CALR,HSP90AB1,PRDX6	5
Cancer, Endocrine System Disorders	benign neoplasm of endocrine gland	6.88E-06	ANXA2,ANXA5,APOA1,CALR,DUT,HSP90AB1,LGALS3,PRDX6,TGM2,TUBB4A	10
Cell Death and Survival	apoptosis of tumor cell lines	7.10E-06	ALB,ANXA2,CALR,CAPNS1,CCT2,DUT,DYNC1H1,EIF4B,ELAVL1,ENO1,EZR,GSN,H2AFX,HINT1,HNRNPC,HSP90AB1,HSPD1,LGALS3,MSN,MTDH,NCL,NDRG1,NQO1,RPS3,SFN,TAGLN2,TF,TGM2,TPD52,YBX1,YWHAE	31
Dermatological Diseases and Conditions, Immunological Disease, Inflammatory Disease, Inflammatory Response	atopic dermatitis	7.87E-06	AHNAK,ANXA5,CFL1,CTSL,ENO1,FABP5,FLNA,GSN,HNRNPR,IDI1,KRT15,MSN	12
Cancer	benign neoplasia	9.15E-06	AK2,ALB,ALDOA,ANXA2,ANXA5,APOA1,CALR,CYBA,DUT,FABP5,HINT1,HSD17B10,HSP90AB1,KRT15,LGALS3,NDRG1,NF2,PRDX6,SF3A1,TGM2,TUBB4A	21
Cell Death and Survival	cell survival	9.79E-06	ALB,ANXA5,ATP5H,CALR,CYBA,DHX9,ELAVL1,EZR,FGF6,FLNA,H2AFX,HINT1,HSD17B10,HSP90AB1,HSPD1,LDHA,LGALS3,LMNA,MTDH,NDRG1,PDIA3,PFDN2,PPIB,PRDX6,PSMA7,PSMB4,SF3A1,SFN,SHMT2,TGM2,TXNDC5,UBQLN2,VIM,YBX1	34
Cellular Development, Cellular Growth and	proliferation of tumor cell lines	1.20E-05	AHSG,ALDOA,ANXA2,ANXA6,C1QBP,CALR,CDC37,CIRBP,DYNC1H1,EEF1B2,EIF4B,ELAVL1,EPB41L3,EZR,FABP5,FGF6,FLNA,HNRNPA2B1,LGALS3,M	35

Proliferation			APRE1,NCL,NDRG1,NF2,NQO1,PDIA3,PRKCSH,PTBP1,RPS14,SFN,TAGLN2,TF,TGM2,TPD52,YBX1,YWHAG	
Cancer, Endocrine System Disorders, Organismal Injury and Abnormalities, Reproductive System Disease	ovarian cancer	1.29E-05	AGR2,APOA1,ATIC,ENO1,H2AFX,HSP90AB1,LMNA,PDIA3,PSMA8,PSMB4,SERPINA1,SHMT2,TGM2,TPD52,TUBB4A,VIM	16
Cellular Movement	cell movement of tumor cell lines	1.41E-05	AGR2,ANXA2,C1QBP,CALR,CAPNS1,CTSL,EZR,FLNA,FLNC,HNRNPA2B1,LGALS3,MSN,MTDH,MYH9,NCL,NDRG1,NF2,SERPINA1,SFN,TAGLN2,TGM2,VIM,YBX1	23
DNA Replication, Recombination, and Repair, Nucleic Acid Metabolism, Small Molecule Biochemistry	catabolism of nucleoside triphosphate	1.76E-05	ATP5A1,ATP5D,ATP5H,ATP5O,DCTPP1,DDX39B,DHX9,MYH9,RHOBTB3	9
Cellular Function and Maintenance	internalization of cells	2.14E-05	APOA1,APOA2,EZR,LGALS3,NCL,SNAP29,TGM2,VIM	8
Neurological Disease	progressive motor neuropathy	2.35E-05	ANXA2,ANXA5,CCT2,DBI,EZR,GSN,IMPDH2,LAMP1,LDHA,PDIA3,PGK1,RPL13,RPL3,TCEA1,TGM2,UBQLN2,VIM	17
Cellular Function and Maintenance	engulfment of cells	2.42E-05	AHSG,ANXA5,APOA1,APOA2,CALR,EHD1,EZR,GSN,LGALS3,MYH9,NCL,RPS21,SF3B4,TGM2,VIM	15
Energy Production, Nucleic Acid Metabolism, Small Molecule Biochemistry	metabolism of ATP	2.56E-05	AK2,ATP5A1,ATP5D,ATP5H,ATP5O,DDX39B,DHX9,MYH9,RHOBTB3	9
RNA Post-Transcriptional Modification	splicing of mRNA	2.72E-05	C1QBP,DDX39B,HNRNPA2B1,HNRNPH3,HNRNPM,PTBP1,SF3A1	7
Endocrine System Disorders, Gastrointestinal Disease,	autoimmune pancreatitis	2.95E-05	ALB,ANXA4,APOA1,CALR	4

Immunological Disease, Inflammatory Disease				
Cellular Assembly and Organization, Cellular Function and Maintenance	organization of actin cytoskeleton	2.97E-05	ALDOA,CALR,CFL1,EZR,FLNA,FLNC,GSN,MSN,MYH9,NF2,TF,TGM2	12
Neurological Disease, Psychological Disorders	disorder of basal ganglia	3.07E-05	ANXA2,ATP5O,BASP1,CAPNS1,CIRBP,HINT1,LAMP1,LDHA,MTDH,NDRG1,PGK1,PRDX6,RHOBTB3,RPL13,RPL3,SDHA,SDHB,SERPINA1,TGM2,TPD52,TUBB4A,VIM	22
Dermatological Diseases and Conditions, Inflammatory Disease, Inflammatory Response	Dermatitis	3.12E-05	AHNAK,ANXA5,CALR,CFL1,CTSL,ENO1,FABP5,FLNA,GSN,HNRNPR,IDI1,KRT15,MSN,SFN,TUBB4A	15
Nucleic Acid Metabolism	metabolism of nucleic acid component or derivative	3.14E-05	AK2,ALDOA,APOA1,ATIC,ATP5A1,ATP5D,ATP5H,ATP5O,DBI,DCTPP1,DDX39B,DHX9,DUT,HSPD1,IMPDH2,MYH9,NQO1,PGK1,RHOBTB3,TFAM	20
Cancer	metastasis	3.38E-05	AGR2,AHSG,ANXA5,APOA1,ATIC,C1QBP,EPB41L3,EZR,FABP5,FLNA,HSP90AB1,LGALS3,NDRG1,NF2,NQO1,SERPINA1,TCEA1,TGM2,TUBB4A,VIM,YWHAE	21
Cell Death and Survival	cell death of lymphoma cell lines	3.68E-05	ALB,ANXA2,DUT,EZR,HNRNPC,LGALS3,MSN,NCL,TGM2,YWHAG	10
Hematological Disease	blood protein disorder	3.82E-05	AGR2,AHNAK2,ALB,ANXA2,ANXA4,ANXA6,CTSL,FLNA,GSN,LAMP1,TF,VIM,YWHAE	13
Cancer	adenoma	3.98E-05	ANXA2,ANXA5,APOA1,CALR,DUT,HINT1,HSP90AB1,KRT15,LGALS3,PRDX6,TGM2,TUBB4A	12
Organismal Injury and Abnormalities	nodule	4.41E-05	ANXA5,APOA1,CALR,HSP90AB1,PRDX6,TGM2	6
Cellular Movement	migration of cells	4.77E-05	AHSG,ALB,ALDOA,ANXA2,ANXA5,APOA1,C1QBP,CALR,CAPNS1,CFL1,CTSL,EHD1,EZR,FABP5,FGF6,FLNA,FLNC,GSN,HNRNPA2B1,HSP90AB1,HSPD1,LGALS3,LMNA,MAPRE1,MCM3,MSN,MTDH,MYH9,NARS,NCL,NDRG1,NF2,	40

			NQO1,PPIB,SERPINA1,SFN,TGM2,VIM,YBX1,YWHAE	
Cell Morphology, Connective Tissue Development and Function	cell spreading of fibroblast cell lines	5.07E-05	EHD1,FLNA,FLNC,PTBP1,VIM	5
Organismal Survival	organismal death	5.28E-05	AGR2,APOA1,C1QBP,CALR,CANX,CAPNS1,CAPRIN1,CFL1,DBI,DHX9,DLD,DYNC1H1,EHD1,ELAVL1,EPB41L3,FLNA,FLNC,GNPNAT1,GSN,GSS,H2AFX,HNRNPC,HSP90AB1,IMPDH2,LAMP1,LGALS3,LMNA,MSN,MTDH,MYH9,NDRG1,NF2,NQO1,PDIA3,PPIB,PSMB4,PTBP1,RPL6,SERPINA1,SHMT2,TCEA1,TFAM,TGM2,TKT,VIM,YBX1,YWHAE	47
Cellular Function and Maintenance, Inflammatory Response	phagocytosis	5.42E-05	AHSG,ANXA5,APOA1,APOA2,CALR,EHD1,GSN,LGALS3,MSN,MYH9,TGM2,VIM	12
Cellular Movement	cell movement	5.44E-05	AGR2,AHSG,ALB,ALDOA,ANXA2,ANXA5,APOA1,C1QBP,CALR,CAPNS1,CFL1,CTSL,EHD1,EZR,FABP5,FGF6,FLNA,FLNC,GSN,HNRNPA2B1,HSP90AB1,HSPD1,LGALS3,LMNA,MAPRE1,MCM3,MSN,MTDH,MYH9,NARS,NCL,NDRG1,NF2,NQO1,PPIB,SERPINA1,SFN,ST13,TAGLN2,TGM2,VIM,YBX1,YWHAE	43
Nucleic Acid Metabolism, Small Molecule Biochemistry	synthesis of ADP	5.57E-05	AK2,ATP5A1,ATP5D	3
Cancer	advanced malignant tumor	5.65E-05	AGR2,AHSG,ANXA5,APOA1,ATIC,C1QBP,EPB41L3,EZR,FABP5,FLNA,H2AFX,HSP90AB1,LGALS3,NDRG1,NF2,NQO1,SERPINA1,TCEA1,TGM2,TUBB4A,VIM,YWHAE	22
Cell Death and Survival	cell viability	7.10E-05	ALB,ANXA5,ATP5H,CALR,CYBA,DHX9,ELAVL1,EZR,FLNA,H2AFX,HINT1,HSD17B10,HSP90AB1,HSPD1,LDHA,LGALS3,LMNA,MTDH,NDRG1,PDIA3,PFDN2,PRDX6,PSMB4,SF3A1,SFN,SHMT2,TGM2,TXNDC5,UBQLN2,YBX1	30
Neurological Disease, Skeletal and Muscular Disorders	neuromuscular disease	7.23E-05	ANXA2,ATP5O,BASP1,CAPNS1,CIRBP,HINT1,IMPDH2,LAMP1,LDHA,MTDH,NDRG1,PDIA3,PGK1,PRDX6,RHOBTB3,RPL13,RPL3,SDHA,SDHB,SERPINA1,TGM2,TPD52,VIM	23
Cancer, Gastrointestinal Disease, Hepatic	biliary tract cancer	8.49E-05	ALB,ANXA2,HSP90AB1,NQO1,PGK1,VIM	6

System Disease				
Cell-To-Cell Signaling and Interaction, Cellular Function and Maintenance, Inflammatory Response	phagocytosis of cells	9.52E-05	AHSG,ANXA5,APOA1,APOA2,CALR,EHD1,GSN,LGALS3,MYH9,TGM2,VIM	11
Cell Morphology, Connective Tissue Development and Function	shape change of fibroblast cell lines	9.58E-05	EHD1,FLNA,FLNC,NF2,PTBP1,VIM	6
DNA Replication, Recombination, and Repair, Energy Production, Nucleic Acid Metabolism, Small Molecule Biochemistry	catabolism of ATP	1.02E-04	ATP5A1,ATP5D,ATP5H,ATP5O,DDX39B,DHX9,MYH9,RHOBTB3	8
Cancer, Gastrointestinal Disease, Hepatic System Disease	cholangiocarcino ma	1.03E-04	ALB,ANXA2,HSP90AB1,PGK1,VIM	5
Neurological Disease	Movement Disorders	1.05E-04	ANXA2,ATP5O,BASP1,CANX,CAPNS1,CIRBP,EPB41L3,HINT1,LAMP1,LDHA, LMNA,MTDH,NDRG1,PGK1,PRDX6,RHOBTB3,RPL13,RPL3,SDHA,SDHB,SER PINA1,TFAM,TGM2,TPD52,TUBB4A,VIM	26
Immunological Disease	hypersensitive reaction	1.09E-04	AHNAK,ALB,ANXA5,CALR,CFL1,CTSL,ENO1,FABP5,FLNA,GSN,HNRNPR,I DI1,KRT15,MSN	14
Carbohydrate Metabolism	glycolysis	1.10E-04	ALDOA,C1QBP,CYBA,ENO1,LDHA,PGK1,PGM1	7
Cellular Assembly and Organization, Cellular Function and Maintenance	organization of filaments	1.19E-04	ALDOA,ANXA2,CFL1,FLNA,GSN,MAPRE1,MSN,TF,VIM	9
DNA Replication,	metabolism of	1.24E-04	CALR,DHX9,DUT,ENO1,HSD17B10,LMNA,NAP1L1,NCL,NF2,PPIB,PRDX6,SE	14

Recombination, and Repair	DNA		RPINA1,TFAM,TMPO	
Embryonic Development, Tissue Morphology	abnormal morphology of visceral endoderm	1.28E-04	DHX9,DLD,MYH9,NF2	4
Cellular Assembly and Organization	stabilization of actin filaments	1.31E-04	CFL1,FLNA,GSN	3
Cell Morphology, Renal and Urological System Development and Function	shape change of kidney cell lines	1.40E-04	ANXA2,FLNA,FLNC,NF2,VIM	5
Cellular Movement	invasion of prostate cancer cell lines	1.60E-04	AGR2,EZR,FABP5,LGALS3,NDRG1,VIM	6
Cellular Movement	migration of tumor cell lines	1.69E-04	ANXA2,C1QBP,CAPNS1,CTSL,EZR,FLNA,FLNC,HNRNPA2B1,LGALS3,MSN, MTDH,MYH9,NCL,NDRG1,NF2,SFN,TGM2,VIM	18
Connective Tissue Disorders, Metabolic Disease, Skeletal and Muscular Disorders	metabolic bone disease	1.75E-04	ALB,FLNA,HNRNPA2B1,HNRNPC,PPIB	5
Nucleic Acid Metabolism, Small Molecule Biochemistry	metabolism of nucleotide	1.77E-04	AK2,ALDOA,APOA1,ATP5A1,ATP5D,ATP5H,ATP5O,DCTPP1,DDX39B,DHX9, DUT,HSPD1,IMPDH2,MYH9,NQO1,RHOBTB3,TFAM	17
Gastrointestinal Disease, Hepatic System Disease, Metabolic Disease	amyloidosis of liver	1.85E-04	APOA1,APOA2	2
Immunological Disease, Metabolic Disease	amyloidosis of spleen	1.85E-04	APOA1,APOA2	2
Lipid Metabolism, Molecular Transport, Small Molecule Biochemistry	depletion of glycosphingolipid	1.85E-04	APOA1,TFAM	2

Cellular Movement	initiation of migration of fibrosarcoma cell lines	1.85E-04	FLNA,FLNC	2
Cellular Assembly and Organization	linkage of actin cytoskeleton	1.85E-04	EZR,MSN	2
Lipid Metabolism, Small Molecule Biochemistry	recruitment of phospholipid	1.85E-04	APOA1,APOA2	2
Inflammatory Disease	chronic inflammatory disorder	1.94E-04	AHSG,ALB,ALDOA,ANXA4,APOA1,APOA2,ATIC,CALR,CTSL,CYBA,DDX39B,EEF1G,ENO1,GSN,HSPD1,IMPDH2,MAPRE1,OR11A1,PDIA3,PGK1,RAB10,RPS3,VIM	23
Carbohydrate Metabolism	glycolysis of cells	2.18E-04	ALDOA,C1QBP,ENO1,LDHA,PGK1,PGM1	6
Cellular Movement	cell movement of breast cancer cell lines	2.27E-04	ANXA2,CTSL,FLNA,MYH9,NDRG1,NF2,SERPINA1,SFN,TGM2,VIM	10
Cellular Assembly and Organization, Cellular Function and Maintenance	organization of actin filaments	2.41E-04	ALDOA,CFL1,FLNA,GSN,MSN,TF	6
Cellular Development	differentiation of cells	2.45E-04	ALB,ANXA2,ANXA6,BASP1,C1QBP,C1QC,CALR,CAPNS1,CFL1,CLIC1,DHX9,ELAVL1,ENO1,EZR,FABP5,FGF6,FLNC,GSN,HNRNPA2B1,HNRNPC,HSP90AB1,HSPD1,LAMP1,LGALS3,LMNA,NAP1L1,NDRG1,NF2,PDIA3,RPS14,RPS7,SF3B4,SFN,SYNCRIP,TAGLN2,TCEA1,TF,TFAM,TGM2,VIM,YBX1,YWHAG	42
Cancer, Endocrine System Disorders	neuroendocrine tumor	2.51E-04	ANXA2,DUT,HSP90AB1,HSPD1,LGALS3,SDHA,SDHB,TGM2,TUBB4A,YWHAE	10
Neurological Disease	neurological signs	2.64E-04	APOA1,ATP5O,BASP1,CAPNS1,CIRBP,HINT1,HNRNPA2B1,LDHA,MTDH,NDRG1,PGK1,PRDX6,RHOBTB3,SDHA,SDHB,SERPINA1,TGM2,TPD52	18
Cellular Movement	migration of breast cancer cell lines	2.82E-04	ANXA2,CTSL,FLNA,MYH9,NDRG1,NF2,SFN,TGM2,VIM	9
Cell Death and Survival	apoptosis of lymphoma cell lines	3.09E-04	ALB,DUT,EZR,HNRNPC,LGALS3,MSN,NCL,TGM2	8

Cancer	mesothelioma	3.61E-04	ATIC,HSP90AB1,NF2,TUBB4A,VIM	5
Inflammatory Disease, Skeletal and Muscular Disorders	inclusion body myopathy	3.62E-04	CALR,CANX,HNRNPA2B1	3
Cardiovascular Disease, Metabolic Disease, Organismal Injury and Abnormalities	cardiac amyloidosis	3.68E-04	APOA1,GSN	2
Cell-To-Cell Signaling and Interaction, Tissue Development	growth of focal adhesions	3.68E-04	FLNA,VIM	2
Cancer, Endocrine System Disorders	hereditary paraganglioma	3.68E-04	SDHA,SDHB	2
Developmental Disorder, Hereditary Disorder, Metabolic Disease	hereditary systemic amyloidosis	3.68E-04	APOA1,GSN	2
Energy Production	quantity of pyruvic acid	3.68E-04	ALDOA,NQO1	2
Cell Death and Survival	cell death of colon cancer cell lines	3.77E-04	CYBA,GSN,HINT1,HSPD1,LGALS3,LMNA,NDRG1,SFN,TGM2,YWHAE	10
Carbohydrate Metabolism, Lipid Metabolism, Small Molecule Biochemistry	metabolism of phosphatidylcholine	3.98E-04	APOA1,APOA2,FABP5,PRDX6	4
Developmental Disorder, Hereditary Disorder, Skeletal and Muscular Disorders	distal myopathy	4.27E-04	CALR,CANX,FLNC	3
Cellular Function and Maintenance	engulfment of phagocytes	4.28E-04	ANXA5,APOA1,APOA2,CALR,GSN,LGALS3,TGM2	7

Hematological Disease	anemia	4.58E-04	AGR2,ALDOA,C1QBP,GSS,IMPDH2,LGALS3,NQO1,RPS10,RPS7,TCEA1,TF	11
Organismal Injury and Abnormalities, Skeletal and Muscular Disorders	neurogenic muscular atrophy	4.73E-04	DYNC1H1,HINT1,LMNA,NDRG1	4
Cancer	Cancer	4.77E-04	AGR2,AHNAK,AHNAK2,AHSG,AK2,ALB,ALDOA,ANXA2,ANXA4,ANXA5,ANXA6,APOA1,APOA2,ARMC1,ARMCX4,ATIC,ATP5A1,ATP5O,BASP1,C1QBP,C1QC,CALR,CANX,CAPRIN1,CCT2,CDC37,CFL1,CHMP4B,CIRBP,CLIC1,CNDP2,COX4I1,CTSL,CYBA,DBI,DCTPP1,DDX39B,DHX9,DLD,DYNC1H1,EEF1B2,EEF1D,EFHD2,EHD1,EIF4B,EIF4H,ELAVL1,ENO1,EPB41L3,EZR,FABP5,FGF6,FLNA,FLNC,GNPNAT1,GSN,GSS,H2AFX,HBD,HINT1,HNRNPA2B1,HNRNPC,HNRNPF,HNRNPH1,HNRNPH3,HNRNPM,HNRNPR,HSP90AB1,HSPD1,IDI1,IMPDH2,ITIH2,KRT15,LAMP1,LDHA,LGALS3,LMNA,LRRC59,MAPRE1,MCM3,MROH5,MSN,MTDH,MYH9,NAP1L1,NCL,NDRG1,NF2,NQO1,NUCB1,OR11A1,PABPC1,PAFAH1B2,PDIA3,PGK1,PGM1,PLIN3,PRKCSH,PSMA7,PSMA8,PSMB3,PSMB4,PSPC1,PTBP1,PTRF,RAB10,RCN2,RHOBTB3,RPL12,RPL3,RPL6,RPS23,RPS3,RPS7,RPS9,SDHA,SDHB,SERPINA1,SERPINB6,SF3A1,SF3B4,SFN,SH3BGRL3,SHMT2,SNAP29,SYNCRIP,TAGLN2,TCEA1,TF,TFAM,TGM2,TMPO,TPD52,TUBB4A,TXLNA,TXNDC5,UBQLN2,VIM,YBX1,YWHAE,YWHAG	141
Cellular Movement	invasion of cells	4.95E-04	AGR2,AHSG,ANXA2,C1QBP,CALR,CAPNS1,CTSL,EZR,FABP5,FLNA,GSN,HSP90AB1,LGALS3,MTDH,NDRG1,NF2,NQO1,SERPINA1,TAGLN2,VIM	20
Tissue Morphology	abnormal morphology of ectoplacental cone	5.00E-04	DHX9,GNPNAT1,NF2	3
Hereditary Disorder, Neurological Disease, Psychological Disorders, Skeletal and Muscular Disorders	Huntington's Disease	5.13E-04	ATP5O,BASP1,CAPNS1,CIRBP,HINT1,LDHA,MTDH,NDRG1,PGK1,PRDX6,RHOBTB3,SDHA,SDHB,SERPINA1,TGM2,TPD52	16
Cell Morphology, Cellular Function and Maintenance	transmembrane potential of mitochondria	5.26E-04	ANXA6,CLIC1,GSN,HSPD1,LDHA,NDRG1,TGM2,TPD52,YWHAE	9
Protein Synthesis	homo-oligomerization	5.90E-04	ANXA5,ANXA6,CHMP4B,EHD1,ST13,TGM2	6

	of protein			
Cardiovascular Disease	vascular disease	6.49E-04	AHSG,ALB,ANXA2,ANXA5,APOA1,APOA2,CTSL,EEF1B2,EIF4H,FABP5,FLNA,GSN,GSS,H2AFX,HEBP2,HSPD1,IMPDH2,LGALS3,RCN2,TKT,TUBB4A,VIM	22
Nucleic Acid Metabolism, Small Molecule Biochemistry	biosynthesis of purine ribonucleotide	6.65E-04	AK2,ALDOA,ATP5A1,ATP5D,HSPD1,TFAM	6
Dermatological Diseases and Conditions	psoriasis	6.99E-04	ANXA2,C1QBP,CALR,CFL1,CTSL,FABP5,GSN,H2AFX,KRT15,LGALS3,PTRF,SFN,SYNCRIP,TF,YWHAE	15
Cardiovascular Disease, Organismal Injury and Abnormalities	dilated cardiomyopathy	7.52E-04	LMNA,MYH9,PGK1,SDHA,SDHB,TFAM,TMPO	7
Cancer, Hematological Disease, Immunological Disease	plasma cell dyscrasia	7.58E-04	AHNAK2,ANXA2,ANXA4,ANXA6,CTSL,FLNA,GSN,LAMP1,VIM,YWHAE	10
Cell Morphology, Renal and Urological System Development and Function	cell spreading of kidney cell lines	7.63E-04	FLNA,FLNC,VIM	3
Infectious Disease	replication of RNA virus	7.88E-04	ANXA5,ANXA6,DHX9,HNRNPM,HSP90AB1,HSPD1,NCL,PLIN3,RPS10,RPS14,SERPINA1,SF3A1,TMPO,YBX1	14
Cellular Growth and Proliferation	colony formation	8.00E-04	ATIC,C1QBP,DYNC1H1,ELAVL1,ENO1,EPB41L3,EZR,HNRNPA2B1,LGALS3,NDRG1,NF2,SERPINA1,SFN,TPD52	14
Infectious Disease	infection of cells	8.01E-04	ANXA2,C1QBP,CCT2,CTSL,DHX9,HNRNPF,HNRNPH1,NF2,PDIA3,PGM1,PPIB,PSMA7,RPL12,RPL3,SERPINA1,SERPINB6,SF3A1,TAGLN2,YBX1	19
Cancer, Organismal Injury and Abnormalities, Reproductive System Disease	Prostate Cancer and Tumors	8.23E-04	AGR2,ANXA2,APOA1,APOA2,CCT2,CIRBP,EEF1B2,EPB41L3,FLNA,FLNC,HNRNPH1,KRT15,LGALS3,PGK1,SHMT2,TUBB4A,YBX1	17
Cell Cycle	arrest in G2 phase	8.25E-04	ANXA2,ELAVL1,FLNA,MTDH,NDRG1,SFN,UBQLN2	7

Inflammatory Response	inflammation of body cavity	8.44E-04	AGR2,ALB,ALDOA,ANXA5,APOA1,APOA2,CTSL,ENO1,IMPDH2,LGALS3,MYH9,NQO1,PDIA3,PRDX6,TGM2,TKT,YBX1	17
Infectious Disease	release of virus	8.72E-04	ANXA6,CHMP4B,FLNA,LMNA	4
Cellular Assembly and Organization, Cellular Function and Maintenance	organization of cytoskeleton	8.95E-04	ALDOA,BASP1,C1QBP,CALR,CANX,CAPNS1,CAPRIN1,CFL1,EHD1,EPB41L3,EZR,FLNA,FLNC,GSN,MAPRE1,MSN,MYH9,NDRG1,NF2,NQO1,PDIA3,PRKCSH,RAB10,TF,TGM2,TUBB4A,VIM,YBX1	28
Hereditary Disorder, Skeletal and Muscular Disorders	autosomal recessive myopathy	9.01E-04	AHNAK,CALR,CANX,HINT1,LMNA	5
RNA Post-Transcriptional Modification	deadenylation of mRNA	9.11E-04	PABPC1,SYNCRIP	2
Cell-To-Cell Signaling and Interaction, Cellular Function and Maintenance, Inflammatory Response	phagocytosis of bone marrow cell lines	9.11E-04	APOA1,APOA2	2
Connective Tissue Disorders, Metabolic Disease, Nutritional Disease, Skeletal and Muscular Disorders	vitamin D-resistant rickets	9.11E-04	HNRNPA2B1,HNRNPC	2
DNA Replication, Recombination, and Repair, Energy Production, Nucleic Acid Metabolism, Small Molecule Biochemistry	hydrolysis of ATP	9.35E-04	APOA1,EIF4B,HSPD1,TGM2	4
Cellular Function and Maintenance	engulfment of antigen presenting cells	9.37E-04	APOA1,APOA2,CALR,GSN,LGALS3,TGM2	6

Connective Tissue Disorders, Immunological Disease, Inflammatory Disease, Skeletal and Muscular Disorders	rheumatoid arthritis	9.40E-04	AHSG,ALB,ALDOA,APOA1,ATIC,CALR,DDX39B,EEF1G,ENO1,GSN,HSPD1,MAPRE1,PDIA3,PGK1,RAB10,RPS3,VIM	17
Cell Morphology, Cellular Function and Maintenance	transmembrane potential	9.46E-04	ANXA6,CLIC1,DLD,GSN,HSPD1,LDHA,NDRG1,TGM2,TPD52,YWHAE	10
RNA Post-Transcriptional Modification	splicing of primary transcript RNA	9.79E-04	AHNAK,HNRNPF,HNRNPH1	3
Inflammatory Response, Respiratory Disease	inflammation of lung	9.79E-04	ALB,ALDOA,ANXA5,ENO1,IMPDH2,LGALS3,MYH9,NQO1,PDIA3,PRDX6,TKT	11
Cancer, Organismal Injury and Abnormalities, Reproductive System Disease	cervical tumor	9.88E-04	ANXA2,ANXA5,ATIC,CTSL,DUT,ENO1,HSP90AB1,TUBB4A,YWHAE	9

Table S9. Disease and function analysis of mapped differentially expressed proteins between LNCaP and LNCaPRR cell lines

**Categories**	**Diseases or Functions Annotation**	**p-Value**	**Molecules**	**Molecules number**
Cellular Growth and Proliferation	proliferation of cells	4.23E-10	AGR2,AHNAK,AHSG,AK2,ALB,ALDOA,ANXA2,ANXA6,APOA1,ATIC,ATP5A1,BASP1,C1QBP,CALR,CAPNS1,CAPRIN1,CDC37,CFL1,CTSL,CYBA,DBI,DLD,DYNC1H1,EEF1B2,EEF1D,EIF4B,ELAVL1,ENO1,EPB41L3,EZR,FABP5,FGF6,FLNA,GNPNAT1,GSN,GSS,H2AFX,HINT1,HNRNPC,HNRNPF,HNRNPM,HNRNPR,HSPD1,LDHA,LGALS3,LMNA,MCM3,MTDH,MYH9,NAP1L1,NCL,NDRG1,NF2,NQO1,NUDC,PLIN3,PPIB,PRKCSH,PTBP1,RPS14,RPS9,SDHB,SFN,SHMT2,TF,TFAM,TGM2,TMPO,TPD52,TXLNA,TXNDC5,VIM,YBX1,YWHAG	74
Protein Synthesis	translation	9.45E-10	CALR,CAPRIN1,DHX9,EEF1B2,EIF4B,EIF4H,ELAVL1,NCL,PTBP1,RPS14,RPS3,RPS7,RPS9,SYNCRIP,TUFM,YBX1	16
Protein Synthesis	metabolism of protein	1.33E-09	APOA1,APOA2,CALR,CANX,CAPNS1,CAPRIN1,CDC37,CNDP2,CTSL,DHX9,DLD,EEF1B2,EIF4B,EIF4H,ELAVL1,FLNA,GSN,HSPD1,LAMP1,MYH9,NCL,NF2,PPIB,PTBP1,RPS14,RPS3,RPS7,RPS9,SYNCRIP,TGM2,TUFM,YBX1	32
Cell Death and Survival	cell death	1.66E-09	AGR2,ALB,ALDOA,ANXA2,ANXA4,ANXA5,APOA1,ATP5A1,BASP1,C1QBP,CALR,CANX,CAPNS1,CAPRIN1,CDC37,CFL1,CTSL,CYBA,DHX9,DUT,DYNC1H1,EEF1D,EHD1,EIF4B,ELAVL1,ENO1,EZR,FGF6,FLNA,GNPNAT1,GSN,GSS,H2AFX,HEBP2,HINT1,HNRNPC,HSD17B10,HSPD1,LAMP1,LDHA,LGALS3,LMNA,MSN,MTDH,MYH9,NCL,NDRG1,NF2,NQO1,PAFAH1B2,PLIN3,PPIB,PRDX6,RPS3,SDHA,SDHB,SF3A1,SFN,SH3BGRL3,TF,TFAM,TGM2,TPD52,TUFM,TXNDC5,VIM,YBX1,YWHAE,YWHAG	69
Cancer, Respiratory Disease	respiratory system tumor	4.54E-09	AGR2,AHNAK,AHNAK2,AHSG,AK2,ALDOA,ANXA2,APOA1,ATIC,CAPRIN1,CYBA,DHX9,EEF1B2,ENO1,EZR,FABP5,GSN,HSPD1,ITIH2,LDHA,MSN,MYH9,NCL,NDRG1,NF2,NQO1,PSMB4,SFN,SHMT2,TUBB4A,VIM,YWHAE	32
Protein Synthesis	translation of protein	6.25E-09	CALR,CAPRIN1,DHX9,EEF1B2,EIF4B,EIF4H,ELAVL1,NCL,PTBP1,RPS14,RPS3,RPS7,RPS9,SYNCRIP,YBX1	15
Protein Degradation, Protein Synthesis	stabilization of protein	2.08E-08	APOA1,APOA2,CALR,CANX,CDC37,FLNA,HSPD1,LAMP1,NF2,PPIB	10
Immunological Disease	allergy	3.30E-08	AHNAK,ALB,ALDOA,ANXA5,CFL1,CTSL,ENO1,FABP5,FLNA,GSN,HNRNPR,IDI1,KRT15,MSN,MYH9,PRDX6,TGM2,TKT	18

Cancer, Respiratory Disease	lung tumor	3.36E-08	AGR2,AHNAK,AHNAK2,AHSG,AK2,ALDOA,ANXA2,APOA1,ATIC,CAPRIN1,CYBA,DHX9,EEF1B2,ENO1,EZR,GSN,HSPD1,ITIH2,LDHA,MSN,MYH9,NCL,NDRG1,NF2,NQO1,PSMB4,SHMT2,TUBB4A,VIM,YWHAE	30
Cancer, Respiratory Disease	Lung Cancer and Tumors	3.89E-08	AHNAK,AHNAK2,AHSG,AK2,ALDOA,ANXA2,APOA1,ATIC,CAPRIN1,CYBA,DHX9,EEF1B2,ENO1,EZR,GSN,HSPD1,ITIH2,LDHA,MSN,MYH9,NCL,NF2,NQO1,PSMB4,SHMT2,TFAM,TUBB4A,VIM,YWHAE	29
Cell Death and Survival	apoptosis	4.64E-08	ALB,ALDOA,ANXA2,ANXA4,ANXA5,APOA1,BASP1,C1QBP,CALR,CANX,CAPNS1,CAPRIN1,CDC37,CFL1,CTSL,CYBA,DHX9,DUT,DYNC1H1,EEF1D,EHD1,EIF4B,ELAVL1,ENO1,EZR,FLNA,GNPNAT1,GSN,H2AFX,HEBP2,HINT1,HNRNPC,HSD17B10,HSPD1,LDHA,LGALS3,LMNA,MSN,MTDH,NCL,NDRG1,NF2,NQO1,PAFAH1B2,PRDX6,RPS3,SFN,SH3BGRL3,TF,TFAM,TGM2,TPD52,TXNDC5,VIM,YBX1,YWHAE	56
Cancer	malignant neoplasm of thorax	6.27E-08	AHNAK,AHNAK2,AHSG,AK2,ALDOA,ANXA2,APOA1,ATIC,CAPRIN1,CYBA,DHX9,EEF1B2,ENO1,EZR,GSN,H2AFX,HSPD1,ITIH2,LDHA,MSN,MYH9,NCL,NF2,NQO1,PSMB4,SHMT2,TUBB4A,VIM,YWHAE	29
Cancer, Respiratory Disease	lung cancer	1.20E-07	AHNAK,AHNAK2,AHSG,AK2,ALDOA,ANXA2,APOA1,ATIC,CAPRIN1,CYBA,DHX9,EEF1B2,ENO1,EZR,GSN,HSPD1,ITIH2,LDHA,MSN,MYH9,NCL,NF2,NQO1,PSMB4,SHMT2,TUBB4A,VIM,YWHAE	28
Hematological Disease, Immunological Disease, Inflammatory Disease, Inflammatory Response, Respiratory Disease	allergic pulmonary eosinophilia	1.43E-07	ALB,ALDOA,ENO1,MYH9,PRDX6,TKT	6
Cancer, Respiratory Disease	carcinoma in lung	2.37E-07	AHNAK,AHNAK2,AHSG,AK2,ALDOA,ANXA2,APOA1,ATIC,CAPRIN1,CYBA,DHX9,EEF1B2,ENO1,EZR,GSN,HSPD1,ITIH2,LDHA,MSN,MYH9,NF2,NQO1,TUBB4A,VIM,YWHAE	25
Cell Death and Survival	cell death of tumor cell lines	4.40E-07	AGR2,ALB,ANXA2,ATP5A1,CALR,CAPNS1,CYBA,DUT,DYNC1H1,EIF4B,ELAVL1,ENO1,EZR,FGF6,GSN,H2AFX,HINT1,HNRNPC,HSPD1,LAMP1,LGALS3,LMNA,MSN,MTDH,NCL,NDRG1,NQO1,RPS3,SF3A1,SFN,TF,TGM2,TPD52,TUFM,YBX1,YWHAE,YWHAG	37

RNA Post-Transcriptional Modification	splicing of RNA	4.64E-07	AHNAK,C1QBP,DDX39B,HNRNPF,HNRNPH1,HNRNPH3,HNRNPM,PTBP1,SF3A1,SYNCRIP	10
Post-Translational Modification	conformational modification of protein	6.37E-07	APOA2,CALR,CANX,ERP29,GSN,HSPD1,PFDN2,ST13	8
Nucleic Acid Metabolism, Small Molecule Biochemistry	metabolism of nucleoside triphosphate	8.19E-07	AK2,ALDOA,ATP5A1,ATP5D,ATP5H,ATP5O,DCTPP1,DDX39B,DHX9,HSPD1,MYH9,RHOBTB3,TFAM	13
Cell Death and Survival	necrosis	9.33E-07	AGR2,ALB,ALDOA,ANXA2,APOA1,ATP5A1,CALR,CAPNS1,CAPRIN1,CDC37,CYBA,DUT,DYNC1H1,EEF1D,EIF4B,ELAVL1,ENO1,EZR,FGF6,FLNA,GNPNAT1,GSN,H2AFX,HINT1,HNRNPC,HSD17B10,HSPD1,LAMP1,LDHA,LGALS3,LMNA,MSN,MTDH,NCL,NDRG1,NF2,NQO1,PRDX6,RPS3,SDHA,SDHB,SF3A1,SFN,TF,TFAM,TGM2,TPD52,TUFM,VIM,YBX1,YWHAE,YWHAG	52
Inflammatory Response	inflammation of organ	9.55E-07	AGR2,AHNAK,ALB,ALDOA,ANXA5,APOA1,APOA2,CALR,CFL1,CTSL,ELAVL1,ENO1,FABP5,FLNA,GSN,HNRNPR,IDI1,KRT15,LGALS3,MSN,MYH9,NQO1,PRDX6,SFN,TF,TGM2,TKT,TUBB4A,VIM,YBX1	30
Protein Synthesis	synthesis of protein	1.54E-06	CALR,CAPRIN1,DHX9,EEF1B2,EIF4B,EIF4H,ELAVL1,GSN,NCL,PTBP1,RPS14,RPS3,RPS7,RPS9,SYNCRIP,TUFM,YBX1	17
RNA Post-Transcriptional Modification	processing of RNA	1.73E-06	AHNAK,ALB,C1QBP,DDX39B,HNRNPF,HNRNPH1,HNRNPH3,HNRNPM,PTBP1,RPS7,SF3A1,SF3B4,SYNCRIP	13
Cancer	breast or ovarian cancer	2.23E-06	AGR2,ALDOA,APOA1,ATIC,ATP5A1,C1QBP,CNDP2,CYBA,DYNC1H1,EIF4B,ENO1,FABP5,FLNA,GSN,H2AFX,HSPD1,KRT15,LGALS3,LMNA,MTDH,MYH9,NCL,NQO1,PAFAH1B2,PGM1,PRKCSH,PSMA8,PSMB4,PTBP1,PTRF,RPL6,RPS3,SHMT2,TGM2,TPD52,TUBB4A,VIM	37
Dermatological Diseases and Conditions, Immunological Disease, Inflammatory Disease, Inflammatory	atopic dermatitis	3.01E-06	AHNAK,ANXA5,CFL1,CTSL,ENO1,FABP5,FLNA,GSN,HNRNPR,IDI1,KRT15,MSN	12

Response				
Cancer, Respiratory Disease	non small cell lung adenocarcinoma	3.19E-06	AHNAK,ALDOA,ATIC,CAPRIN1,CYBA,DHX9,EEF1B2,ENO1,EZR,ITIH2,LDHA,MSN,MYH9,NF2,NQO1,TUBB4A	16
Cancer, Respiratory Disease	non-small cell lung cancer	3.57E-06	AHNAK,AHNAK2,AK2,ALDOA,ANXA2,ATIC,CAPRIN1,CYBA,DHX9,EEF1B2,ENO1,EZR,GSN,HSPD1,ITIH2,LDHA,MSN,MYH9,NF2,NQO1,TUBB4A,VIM	22
Cancer	Breast Cancer and Tumors	4.15E-06	AGR2,ALDOA,APOA1,ATP5A1,C1QBP,CDC37,CNDP2,CYBA,DYNC1H1,EIF4B,ENO1,FABP5,FLNA,GSN,HSPD1,KRT15,LGALS3,LMNA,MTDH,MYH9,NCL,NQO1,PAFAH1B2,PGM1,PRKCSH,PSMB4,PTBP1,PTRF,RPL6,RPS3,SHMT2,TPD52,TUBB4A	33
Cancer, Endocrine System Disorders, Organismal Injury and Abnormalities	benign cold thyroid nodule	4.39E-06	ANXA5,APOA1,CALR,PRDX6	4
Post-Translational Modification, Protein Folding	folding of protein	5.67E-06	APOA2,CALR,CANX,ERP29,HSPD1,PFDN2,ST13	7
Cancer	benign neoplasia	7.62E-06	AK2,ALB,ALDOA,ANXA2,ANXA5,APOA1,CALR,CYBA,DUT,FABP5,HINT1,HSD17B10,KRT15,LGALS3,NDRG1,NF2,PRDX6,SF3A1,TGM2,TUBB4A	20
Cellular Function and Maintenance	engulfment of cells	7.92E-06	AHSG,ANXA5,APOA1,APOA2,CALR,EHD1,EZR,GSN,LGALS3,MYH9,NCL,RPS21,SF3B4,TGM2,VIM	15
DNA Replication, Recombination, and Repair, Nucleic Acid Metabolism, Small Molecule Biochemistry	catabolism of nucleoside triphosphate	8.36E-06	ATP5A1,ATP5D,ATP5H,ATP5O,DCTPP1,DDX39B,DHX9,MYH9,RHOBTB3	9

Protein Synthesis	polymerization of protein	8.37E-06	AHNAK,ANXA2,ANXA5,ANXA6,CHMP4B,DCTPP1,EHD1,GSN,HSD17B10,PRKCSH,SHMT2,ST13,TGM2	13
Cell Death and Survival	cell survival	8.82E-06	ALB,ANXA5,ATP5H,CALR,CYBA,DHX9,ELAVL1,EZR,FGF6,FLNA,H2AFX,HINT1,HSD17B10,HSPD1,LDHA,LGALS3,LMNA,MTDH,NDRG1,PFDN2,PPIB,PRDX6,PSMA7,PSMB4,SF3A1,SFN,SHMT2,TGM2,TXNDC5,UBQLN2,VIM,YBX1	32
Cancer	follicular adenoma	9.19E-06	ANXA5,APOA1,CALR,PRDX6	4
Dermatological Diseases and Conditions, Inflammatory Disease, Inflammatory Response	Dermatitis	1.03E-05	AHNAK,ANXA5,CALR,CFL1,CTSL,ENO1,FABP5,FLNA,GSN,HNRNPR,IDI1,KRT15,MSN,SFN,TUBB4A	15
Cancer, Organismal Injury and Abnormalities, Reproductive System Disease	breast cancer	1.03E-05	AGR2,ALDOA,APOA1,ATP5A1,C1QBP,CNDP2,CYBA,DYNC1H1,EIF4B,ENO1,FABP5,FLNA,GSN,HSPD1,KRT15,LGALS3,LMNA,MTDH,MYH9,NCL,NQO1,PAFAH1B2,PGM1,PRKCSH,PSMB4,PTBP1,PTRF,RPL6,RPS3,SHMT2,TPD52,TUBB4A	32
Cellular Function and Maintenance	internalization of cells	1.09E-05	APOA1,APOA2,EZR,LGALS3,NCL,SNAP29,TGM2,VIM	8
Cellular Assembly and Organization, Cellular Function and Maintenance	organization of actin cytoskeleton	1.17E-05	ALDOA,CALR,CFL1,EZR,FLNA,FLNC,GSN,MSN,MYH9,NF2,TF,TGM2	12
Energy Production, Nucleic Acid Metabolism, Small Molecule Biochemistry	metabolism of ATP	1.22E-05	AK2,ATP5A1,ATP5D,ATP5H,ATP5O,DDX39B,DHX9,MYH9,RHOBTB3	9
Gene Expression, Protein Synthesis	translation of mRNA	1.27E-05	CALR,CAPRIN1,EEF1B2,EIF4B,EIF4H,ELAVL1,RPS14,RPS9,SYNCRIP	9

Hematological Disease	blood protein disorder	1.43E-05	AGR2,AHNAK2,ALB,ANXA2,ANXA4,ANXA6,CTSL,FLNA,GSN,LAMP1,TF,VIM,YWHAE	13
Cell Death and Survival	cell death of lymphoma cell lines	1.65E-05	ALB,ANXA2,DUT,EZR,HNRNPC,LGALS3,MSN,NCL,TGM2,YWHAG	10
Cancer, Organismal Injury and Abnormalities, Reproductive System Disease	mammary tumor	1.85E-05	AGR2,ALDOA,APOA1,ATP5A1,C1QBP,CNDP2,CYBA,DYNC1H1,EIF4B,ENO1,FABP5,FLNA,GSN,HSPD1,KRT15,LGALS3,LMNA,MTDH,MYH9,NCL,NF2,NQO1,PAFAH1B2,PGM1,PRKCSH,PSMB4,PTBP1,PTRF,RPL6,RPS3,SHMT2,TPD52,TUBB4A	33
Endocrine System Disorders, Gastrointestinal Disease, Immunological Disease, Inflammatory Disease	autoimmune pancreatitis	2.05E-05	ALB,ANXA4,APOA1,CALR	4
Infectious Disease	Viral Infection	2.13E-05	ALB,ANXA2,ANXA5,ANXA6,CHMP4B,CTSL,DHX9,FLNA,HNRNPC,HNRNPF,HNRNPH1,HNRNPM,HSPD1,LGALS3,LMNA,NCL,NF2,PGM1,PLIN3,PPIB,PSMA7,RPL12,RPL3,RPS10,RPS14,RPS23,SERPINB6,SF3A1,SFN,ST13,TKT,TMPO,TUBB4A,YBX1	34
Cellular Function and Maintenance, Inflammatory Response	phagocytosis	2.17E-05	AHSG,ANXA5,APOA1,APOA2,CALR,EHD1,GSN,LGALS3,MSN,MYH9,TGM2,VIM	12
Cancer, Endocrine System Disorders	benign neoplasm of endocrine gland	2.20E-05	ANXA2,ANXA5,APOA1,CALR,DUT,LGALS3,PRDX6,TGM2,TUBB4A	9
Cell Death and Survival	apoptosis of tumor cell lines	2.29E-05	ALB,ANXA2,CALR,CAPNS1,DUT,DYNC1H1,EIF4B,ELAVL1,ENO1,EZR,GSN,H2AFX,HINT1,HNRNPC,HSPD1,LGALS3,MSN,MTDH,NCL,NDRG1,NQO1,RPS3,SFN,TF,TGM2,TPD52,YBX1,YWHAE	28
Cell Morphology,	cell spreading of	3.25E-05	EHD1,FLNA,FLNC,PTBP1,VIM	5

Connective Tissue Development and Function	fibroblast cell lines			
Immunological Disease	hypersensitive reaction	3.96E-05	AHNAK,ALB,ANXA5,CALR,CFL1,CTSL,ENO1,FABP5,FLNA,GSN,HNRNPR,IDI1,KRT15,MSN	14
Cell-To-Cell Signaling and Interaction, Cellular Function and Maintenance, Inflammatory Response	phagocytosis of cells	4.10E-05	AHSG,ANXA5,APOA1,APOA2,CALR,EHD1,GSN,LGALS3,MYH9,TGM2,VIM	11
Nucleic Acid Metabolism, Small Molecule Biochemistry	synthesis of ADP	4.22E-05	AK2,ATP5A1,ATP5D	3
Protein Degradation, Protein Synthesis	catabolism of protein	5.16E-05	APOA1,APOA2,CALR,CANX,CAPNS1,CDC37,CNDP2,CTSL,DLD,FLNA,GSN,HSPD1,LAMP1,MYH9,NF2,PPIB,TGM2	17
DNA Replication, Recombination, and Repair, Energy Production, Nucleic Acid Metabolism, Small Molecule Biochemistry	catabolism of ATP	5.32E-05	ATP5A1,ATP5D,ATP5H,ATP5O,DDX39B,DHX9,MYH9,RHOBTB3	8
Cell Morphology, Connective Tissue Development and Function	shape change of fibroblast cell lines	5.73E-05	EHD1,FLNA,FLNC,NF2,PTBP1,VIM	6

Cancer	adenoma	7.88E-05	ANXA2,ANXA5,APOA1,CALR,DUT,HINT1,KRT15,LGALS3,PRDX6,TGM2,TUBB4A	11
Cell Death and Survival	cell viability	7.92E-05	ALB,ANXA5,ATP5H,CALR,CYBA,DHX9,ELAVL1,EZR,FLNA,H2AFX,HINT1,HSD17B10,HSPD1,LDHA,LGALS3,LMNA,MTDH,NDRG1,PFDN2,PRDX6,PSMB4,SF3A1,SFN,SHMT2,TGM2,TXNDC5,UBQLN2,YBX1	28
Cancer, Endocrine System Disorders	thyroid adenoma	7.92E-05	ANXA5,APOA1,CALR,PRDX6	4
RNA Post-Transcriptional Modification	processing of mRNA	8.63E-05	ALB,C1QBP,DDX39B,HNRNPH3,HNRNPM,PTBP1,SF3A1,SF3B4	8
Cancer	metastasis	8.67E-05	AGR2,AHSG,ANXA5,APOA1,ATIC,C1QBP,EPB41L3,EZR,FABP5,FLNA,LGALS3,NDRG1,NF2,NQO1,TCEA1,TGM2,TUBB4A,VIM,YWHAE	19
Embryonic Development, Tissue Morphology	abnormal morphology of visceral endoderm	8.97E-05	DHX9,DLD,MYH9,NF2	4
Nucleic Acid Metabolism	metabolism of nucleic acid component or derivative	9.04E-05	AK2,ALDOA,APOA1,ATIC,ATP5A1,ATP5D,ATP5H,ATP5O,DBI,DCTPP1,DDX39B,DHX9,DUT,HSPD1,MYH9,NQO1,RHOBTB3,TFAM	18
Cell Morphology, Renal and Urological System Development and Function	shape change of kidney cell lines	9.08E-05	ANXA2,FLNA,FLNC,NF2,VIM	5
Cellular Movement	invasion of prostate cancer cell lines	9.60E-05	AGR2,EZR,FABP5,LGALS3,NDRG1,VIM	6
Organismal Survival	organismal death	9.73E-05	AGR2,APOA1,C1QBP,CALR,CANX,CAPNS1,CAPRIN1,CFL1,DBI,DHX9,DLD,DYNC1H1,EHD1,ELAVL1,EPB41L3,FLNA,FLNC,GNPNAT1,GSN,GSS,H2AFX,HNRNPC,LAMP1,LGALS3,LMNA,MSN,MTDH,MYH9,NDRG1,NF2,NQO1,PPIB,PSMB4,PTBP1,RPL6,SHMT2,TCEA1,TFAM,TGM2,TKT,VIM,YBX1,YWHAE	43

Cellular Movement	cell movement of tumor cell lines	9.74E-05	AGR2,ANXA2,C1QBP,CALR,CAPNS1,CTSL,EZR,FLNA,FLNC,LGALS3,MSN,MTDH,MYH9,NCL,NDRG1,NF2,SFN,TGM2,VIM,YBX1	20
Cellular Assembly and Organization	stabilization of actin filaments	9.91E-05	CFL1,FLNA,GSN	3
Cancer	advanced malignant tumor	1.25E-04	AGR2,AHSG,ANXA5,APOA1,ATIC,C1QBP,EPB41L3,EZR,FABP5,FLNA,H2AFX,LGALS3,NDRG1,NF2,NQO1,TCEA1,TGM2,TUBB4A,VIM,YWHAE	20
Cellular Movement	migration of breast cancer cell lines	1.40E-04	ANXA2,CTSL,FLNA,MYH9,NDRG1,NF2,SFN,TGM2,VIM	9
Cellular Movement	migration of cells	1.45E-04	AHSG,ALB,ALDOA,ANXA2,ANXA5,APOA1,C1QBP,CALR,CAPNS1,CFL1,CTSL,EHD1,EZR,FABP5,FGF6,FLNA,FLNC,GSN,HSPD1,LGALS3,LMNA,MCM3,MSN,MTDH,MYH9,NARS,NCL,NDRG1,NF2,NQO1,PPIB,SFN,TGM2,VIM,YBX1,YWHAE	36
Cellular Assembly and Organization, Cellular Function and Maintenance	organization of actin filaments	1.46E-04	ALDOA,CFL1,FLNA,GSN,MSN,TF	6
RNA Post-Transcriptional Modification	splicing of mRNA	1.46E-04	C1QBP,DDX39B,HNRNPH3,HNRNPM,PTBP1,SF3A1	6
Gastrointestinal Disease, Hepatic System Disease, Metabolic Disease	amyloidosis of liver	1.54E-04	APOA1,APOA2	2
Immunological Disease, Metabolic Disease	amyloidosis of spleen	1.54E-04	APOA1,APOA2	2
Lipid Metabolism, Molecular Transport, Small Molecule	depletion of glycosphingolipid	1.54E-04	APOA1,TFAM	2

Biochemistry				
Cellular Movement	initiation of migration of fibrosarcoma cell lines	1.54E-04	FLNA,FLNC	2
Cellular Assembly and Organization	linkage of actin cytoskeleton	1.54E-04	EZR,MSN	2
Lipid Metabolism, Small Molecule Biochemistry	recruitment of phospholipid	1.54E-04	APOA1,APOA2	2
Cellular Development, Cellular Growth and Proliferation	proliferation of tumor cell lines	1.57E-04	AHSG,ALDOA,ANXA2,ANXA6,C1QBP,CALR,CDC37,DYNC1H1,EEF1B2,EIF4B,ELAVL1,EPB41L3,EZR,FABP5,FGF6,FLNA,LGALS3,NCL,NDRG1,NF2,NQO1,PRKCSH,PTBP1,RPS14,SFN,TF,TGM2,TPD52,YBX1,YWHAG	30
Cell Death and Survival	apoptosis of lymphoma cell lines	1.64E-04	ALB,DUT,EZR,HNRNPC,LGALS3,MSN,NCL,TGM2	8
Cellular Movement	migration of tumor cell lines	1.65E-04	ANXA2,C1QBP,CAPNS1,CTSL,EZR,FLNA,FLNC,LGALS3,MSN,MTDH,MYH9,NCL,NDRG1,NF2,SFN,TGM2,VIM	17
Cell Death and Survival	cell death of colon cancer cell lines	1.78E-04	CYBA,GSN,HINT1,HSPD1,LGALS3,LMNA,NDRG1,SFN,TGM2,YWHAE	10
DNA Replication, Recombination, and Repair	metabolism of DNA	1.78E-04	CALR,DHX9,DUT,ENO1,HSD17B10,LMNA,NAP1L1,NCL,NF2,PPIB,PRDX6,TFAM,TMPO	13
Nucleic Acid Metabolism, Small Molecule Biochemistry	metabolism of nucleotide	1.87E-04	AK2,ALDOA,APOA1,ATP5A1,ATP5D,ATP5H,ATP5O,DCTPP1,DDX39B,DHX9,DUT,HSPD1,MYH9,NQO1,RHOBTB3,TFAM	16
Neurological Disease,	disorder of basal ganglia	2.13E-04	ANXA2,ATP5O,BASP1,CAPNS1,HINT1,LAMP1,LDHA,MTDH,NDRG1,PRDX6,RHOBTB3,RPL13,RPL3,SDHA,SDHB,TGM2,TPD52,TUBB4A,VIM	19

Psychological Disorders				
Cellular Function and Maintenance	engulfment of phagocytes	2.44E-04	ANXA5,APOA1,APOA2,CALR,GSN,LGALS3,TGM2	7
Dermatological Diseases and Conditions	psoriasis	2.60E-04	ANXA2,C1QBP,CALR,CFL1,CTSL,FABP5,GSN,H2AFX,KRT15,LGALS3,PTRF,SFN,SYNCRIP,TF,YWHAE	15
Cancer, Endocrine System Disorders, Organismal Injury and Abnormalities, Reproductive System Disease	ovarian cancer	2.61E-04	AGR2,APOA1,ATIC,ENO1,H2AFX,LMNA,PSMA8,PSMB4,SHMT2,TGM2,TPD52,TUBB4A,VIM	13
Carbohydrate Metabolism, Lipid Metabolism, Small Molecule Biochemistry	metabolism of phosphatidylcholine	2.80E-04	APOA1,APOA2,FABP5,PRDX6	4
Cellular Movement	cell movement	2.81E-04	AGR2,AHSG,ALB,ALDOA,ANXA2,ANXA5,APOA1,C1QBP,CALR,CAPNS1,CFL1,CTSL,EHD1,EZR,FABP5,FGF6,FLNA,FLNC,GSN,HSPD1,LGALS3,LMNA,MCM3,MSN,MTDH,MYH9,NARS,NCL,NDRG1,NF2,NQO1,PPIB,SFN,ST13,TGM2,VIM,YBX1,YWHAE	38
Organismal Injury and Abnormalities	nodule	2.98E-04	ANXA5,APOA1,CALR,PRDX6,TGM2	5
Cardiovascular Disease, Metabolic Disease, Organismal Injury and	cardiac amyloidosis	3.06E-04	APOA1,GSN	2

Abnormalities				
Cell-To-Cell Signaling and Interaction, Tissue Development	growth of focal adhesions	3.06E-04	FLNA,VIM	2
Cancer, Endocrine System Disorders	hereditary paraganglioma	3.06E-04	SDHA,SDHB	2
Developmental Disorder, Hereditary Disorder, Metabolic Disease	hereditary systemic amyloidosis	3.06E-04	APOA1,GSN	2
Energy Production	quantity of pyruvic acid	3.06E-04	ALDOA,NQO1	2
Developmental Disorder, Hereditary Disorder, Skeletal and Muscular Disorders	distal myopathy	3.25E-04	CALR,CANX,FLNC	3
Organismal Injury and Abnormalities, Skeletal and Muscular Disorders	neurogenic muscular atrophy	3.33E-04	DYNC1H1,HINT1,LMNA,NDRG1	4
Cellular Assembly and Organization, Cellular Function and Maintenance	organization of filaments	3.37E-04	ALDOA,ANXA2,CFL1,FLNA,GSN,MSN,TF,VIM	8

Protein Synthesis	homo-oligomerization of protein	3.61E-04	ANXA5,ANXA6,CHMP4B,EHD1,ST13,TGM2	6
Cancer, Hematological Disease, Immunological Disease	plasma cell dyscrasia	3.66E-04	AHNAK2,ANXA2,ANXA4,ANXA6,CTSL,FLNA,GSN,LAMP1,VIM,YWHAE	10
Tissue Morphology	abnormal morphology of ectoplacental cone	3.81E-04	DHX9,GNPNAT1,NF2	3
Neurological Disease	Movement Disorders	3.97E-04	ANXA2,ATP5O,BASP1,CANX,CAPNS1,EPB41L3,HINT1,LAMP1,LDHA,LMNA,MTDH,NDRG1,PRDX6,RHOBTB3,RPL13,RPL3,SDHA,SDHB,TFAM,TGM2,TPD52,TUBB4A,VIM	23
Nucleic Acid Metabolism, Small Molecule Biochemistry	biosynthesis of purine ribonucleotide	4.07E-04	AK2,ALDOA,ATP5A1,ATP5D,HSPD1,TFAM	6
Cardiovascular Disease	vascular disease	4.59E-04	AHSG,ALB,ANXA2,ANXA5,APOA1,APOA2,CTSL,EEF1B2,EIF4H,FABP5,FLNA,GSN,GSS,H2AFX,HEBP2,HSPD1,LGALS3,RCN2,TKT,TUBB4A,VIM	21
Cell Cycle	arrest in G2 phase	4.76E-04	ANXA2,ELAVL1,FLNA,MTDH,NDRG1,SFN,UBQLN2	7
Carbohydrate Metabolism	glycolysis	4.77E-04	ALDOA,C1QBP,CYBA,ENO1,LDHA,PGM1	6
Cancer, Endocrine System Disorders	neuroendocrine tumor	5.55E-04	ANXA2,DUT,HSPD1,LGALS3,SDHA,SDHB,TGM2,TUBB4A,YWHAE	9
Cellular Function and Maintenance	engulfment of antigen presenting cells	5.76E-04	APOA1,APOA2,CALR,GSN,LGALS3,TGM2	6
Cell Morphology, Renal and Urological System	cell spreading of kidney cell lines	5.82E-04	FLNA,FLNC,VIM	3

Development and Function				
Protein Synthesis	oligomerization of protein	5.90E-04	AHNAK,ANXA5,ANXA6,CHMP4B,EHD1,PRKCSH,ST13,TGM2	8
Hereditary Disorder, Skeletal and Muscular Disorders	autosomal recessive myopathy	5.93E-04	AHNAK,CALR,CANX,HINT1,LMNA	5
Infectious Disease	release of virus	6.16E-04	ANXA6,CHMP4B,FLNA,LMNA	4
DNA Replication, Recombination, and Repair, Energy Production, Nucleic Acid Metabolism, Small Molecule Biochemistry	hydrolysis of ATP	6.61E-04	APOA1,EIF4B,HSPD1,TGM2	4
Cancer, Gastrointestinal Disease	gastric carcinoma	7.03E-04	CANX,DDX39B,HINT1,HNRNPC,HNRNPH1,NQO1,UBQLN2	7
RNA Post-Transcriptional Modification	splicing of primary transcript RNA	7.48E-04	AHNAK,HNRNPF,HNRNPH1	3
Cell-To-Cell Signaling and Interaction, Cellular Function and Maintenance, Inflammatory Response	phagocytosis of bone marrow cell lines	7.57E-04	APOA1,APOA2	2
Cancer, Hematological	Waldenstrom's macroglobulinemi	7.88E-04	ANXA2,ANXA4,ANXA6,CTSL,GSN,LAMP1,YWHAE	7

Disease, Immunological Disease	a			
Hematological Disease	anemia	8.38E-04	AGR2,ALDOA,C1QBP,GSS,LGALS3,NQO1,RPS10,RPS7,TCEA1,TF	10
Cell-To-Cell Signaling and Interaction, Cellular Function and Maintenance, Inflammatory Response	phagocytosis of phagocytes	8.52E-04	ANXA5,APOA1,APOA2,GSN,LGALS3,TGM2	6
Cell Death and Survival	apoptosis of colon cancer cell lines	9.34E-04	GSN,HINT1,HSPD1,LGALS3,NDRG1,SFN,TGM2,YWHAE	8
Skeletal and Muscular Disorders	caveolinopathy	9.38E-04	AHNAK,CALR,CANX,FLNC,LMNA	5

Table S10. Intensity of expression of the representative proteins of top four interested pathways in PC-3 and PC-3RR animal xenografts by immunohistochemistry

**Cell line**	**Immunohistochemistry**[a]							
	P-Akt	P-mTOR	P-4EBP1	VEGF VG-1	VEGF R-2	MCT1	MCT4	CD147
**PC-3 tumor**	2	1	1	1	1	0	1	1
**PC-3RR tumor**	3	3	2	3	3	2	2	3

[a]Immunohistochemistry staining scores: 0=negative;1=weak,2=moderate,3=strong.

Table S11. Intensity of expression of ALDOA in PC-3 and PC-3RR animal xenografts by immunohistochemistry

Cell line	Immunohistochemistry[a]
	ALDOA
PC-3 tumor	3
PC-3RR tumor	1

[a]Immunofluorescence staining scores: 0=negative;1=weak,2=moderate,3=strong.

Table S12. Intensity of expression of different treatment in CaP-RR cells for AO/EB by immunofluorescence

**Cell line**	**immunofluorescence**[a]			
	SCR	6 Gy RT	ALDOA-KD	ALDOA-KD+6 Gy RT
**PC-3RR**	0	2	2	3
**LNCaPRR**	1	2	2	3

Notes: [a]Immunofluorescence staining scores: 0=negative;1=weak,2=moderate,3=strong. KD: knock down; RT: Radiotherapy; SCR-scramble control
